# Expanding the Range of Darobactin Derivatives by Amber Stop Codon Suppression To Introduce Non-canonical Amino Acids

**DOI:** 10.1021/acsomega.4c10307

**Published:** 2025-04-30

**Authors:** Jil-Christine Kramer, Zerlina G. Wuisan, Ute Mettal, Michael Marner, Till F. Schäberle

**Affiliations:** † Natural Product Research Department, Institute for Insect Biotechnology, Justus-Liebig-University Giessen, Ohlebergsweg 12, 35392 Giessen, Germany; ‡ Natural Product Department; Fraunhofer-Institute for Molecular Biology and Applied Ecology (IME), Ohlebergsweg 12, 35392 Giessen, Germany; § German Center for Infection Research (DZIF), Partner Site Giessen-Marburg-Langen, Ohlebergsweg 12, 35392 Giessen, Germany

## Abstract

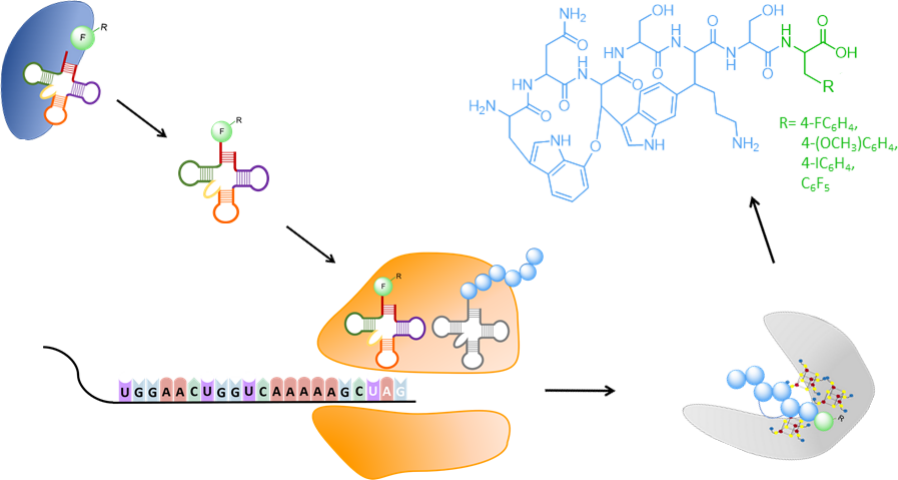

The ribosomally synthesized and post-translationally modified peptide (RiPP) darobactin A is a promising new antibiotic candidate with anti-Gram-negative activity inflicted by the inhibition of the novel target BamA. Genome mining revealed many putative darobactin producer strains, but a limited number of compound modification options. In this study, the amber stop codon suppression technique was used to integrate non-canonical amino acids into the bicyclic heptapeptide, creating new darobactin derivatives. The C-terminal phenylalanine was replaced by non-canonical phenylalanine derivatives with different substituents. Darobactin A F7F, featuring a fluorine atom in the para position of the C-terminal phenylalanine, was purified to enable structure validation by NMR. Activity assays revealed antimicrobial potency against selected Gram-negative strains comparable to darobactin A.

## Introduction

Darobactin A (DAR A) is a ribosomally synthesized and post-translationally modified peptide (RiPP) that consists of seven amino acids (i.e., WNWSKSF). It has a bicyclic structure originating from an ether bridge between W^1^ and W^3^, plus an aryl bridge between W^3^ and K^5^. The natural product was first discovered in *Photorhabdus* species and was observed to possess anti-Gram-negative activity based on a novel mode of action. The compound inhibits the functionality of BamA, an essential part of the outer membrane β-barrel assembly machinery (BAM) complex of Gram-negative bacteria. BamA catalyzes the folding and insertion of outer membrane (OM) proteins like lipoproteins or lipopolysaccharides, with transport, virulence or multidrug resistance function.^1−3^ BamA exists in two major conformations, the lateral-open and the lateral-closed state.^4−6^ As shown by Haysom *et al*., darobactin B (DAR B)^7^ stabilizes the lateral-closed state of BamA, preventing the release of OM proteins from the cytoplasm.^8^ Since OM proteins are essential for cell maintenance, BamA, the essential part of the BAM complex, represents an interesting drug target.^2^ During in vitro studies, DAR A and its derivatives showed promising activity against various Gram-negative bacteria, including multidrug-resistant clinical isolates.^1,9,10^ Furthermore, DAR A and B showed efficacy against resistant **Escherichia coli**, *Klebsiella pneumoniae*, and **Pseudomonas aeruginosa** in mouse models.^1,11^ The biosynthetic gene cluster (BGC) consists of the precursor peptide encoded by *darA*, the *darB*-*D* genes encoding an ABC transporter and the radical SAM (rSAM) enzyme encoded by *darE*.^1^ DarE was proven to catalyze both intramolecular ring closures.^12^ Heterologous expression studies showed that only *darA* and *darE* are essential for the expression and constitute the minimal BGC.^1,13^ In silico analysis of publicly available genome data showed that homologous BGCs are present in many strains from different genera, such as *Yersinia* or *Vibrio*.^1,14^ Variations of the seven core amino acids within the gene sequence of the respective *darA* of these BGCs indicate putative existence of natural analogues, and although several groups described the generation of new analogues by genetic engineering of heterologous producer strains, modifications beyond canonical amino acids are so far limited.^7,11,15,16^ To the best of our knowledge, the only enzyme that was experimentally investigated in this regard is DarH. The halogenase DarH was discovered in marine *Pseudoalteromonas luteoviolaceae* strains and was shown to catalyze bromination or iodination of the C-8 carbon of W^1^.^10^ Another successful attempt to introduce non-canonical amino acids (ncAAs) into the bicyclic heptapeptide was reported by Seyfert *et al*. In this study, feeding assays with non-canonical tryptophane analogues were performed. When feeding chlorinated tryptophane, either W^3^ or W^7^ was modified, and when feeding fluorinated tryptophan, either W^1^, W^7^, or both were fluorinated.^16^ Hence, the position of the modification could not be predicted due to the presence of multiple tryptophan residues in the bicyclic heptapeptide (positions 1, 3, and 7). Therefore, in this study, we adapted the amber stop codon suppression technique to conduct targeted introduction of ncAAs into DAR A to improve the drug-like properties of the compound.^17,18^ The system adapted in this study uses a pyrrolysyl-tRNA synthase- pair from *M. mazei*, which was specifically engineered by Wang *et al*. to accept phenylalanine derivatives with large substituents in the *para* position. Hence, the encoding triplet has to be replaced by the amber stop codon to be recognized by the . Heterologous expression of the modified gene in the presence of the pyrrolysyl-tRNA synthase- pair, should result in the incorporation of a ncAA at the respective position.^19^ Next to this system, there are also different orthogonal translation systems (OTS) derived from other organisms. These systems can not only be used to improve the biological activity of a peptide, but also to introduce spectroscopic probes, add ligands for several chemical reactions, add linkers, or obtain new-to-nature protein properties.^18^ In theory, the strain carrying the amber stop codon could be used to produce multiple derivatives of the desired peptide, since the pyrrolysyl-tRNA synthase can accept various ncAAs depending on which phenylalanine derivative is supplemented to the medium.^19^

## Results and Discussion

**Figure 1:**
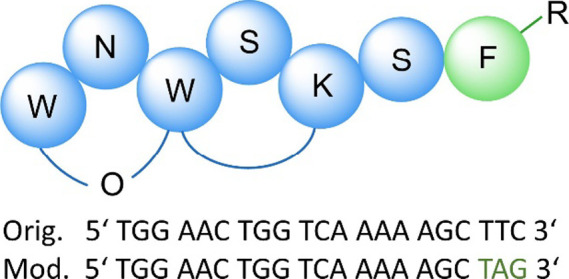
Scheme of DAR A F7-R and the DNA sequence of the DAR A core peptide before and after modification. Blue circles represent natural amino acids of DAR A, while the non-canonical phenylalanine (F) at position seven is displayed in green. The −R represents any desired phenylalanine substituent. The original (Orig.) sequence encoding the DAR A core peptide and the modified version (Mod., marked in green) carrying the amber stop codon are shown below.

In this study, the triplet encoding for the C-terminal phenylalanine F^7^ in the core peptide region of the native *darA* was mutated to the amber stop codon TAG (Figure 1). We coexpressed the modified *darA* plus *darE* on one plasmid and the pyrrolysyl-tRNA synthase- pair on a second plasmid in **E. coli** BL21.

**Figure 2:**
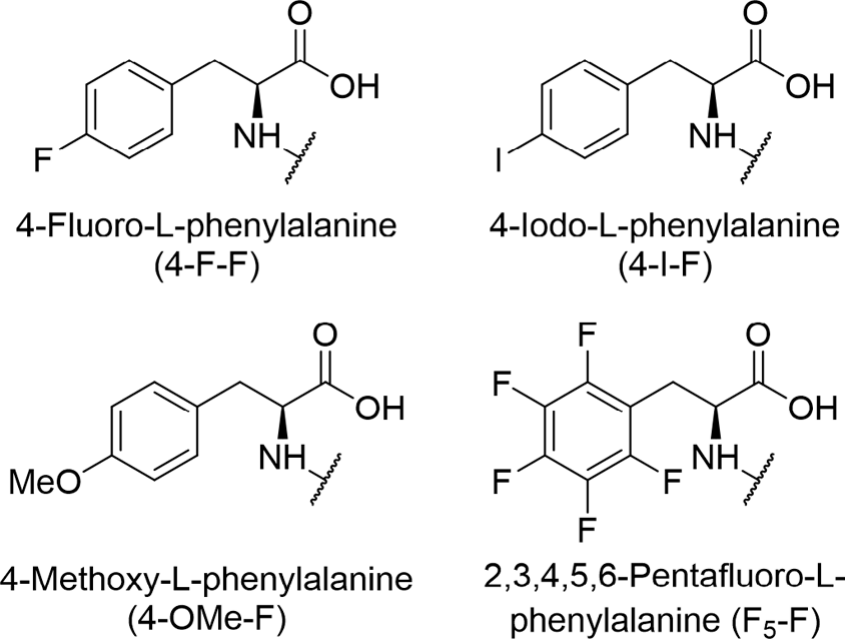
Structures of the non-canonical phenylalanine derivatives successfully integrated in DAR A.

The cultivation was done in minimal medium supplemented with the respective non-canonical phenylalanine (in the absence of canonical phenylalanine). This work resulted in the biosynthesis of new DAR A derivatives with an altered F^7^ residue (Figure 2).

**Figure 3:**
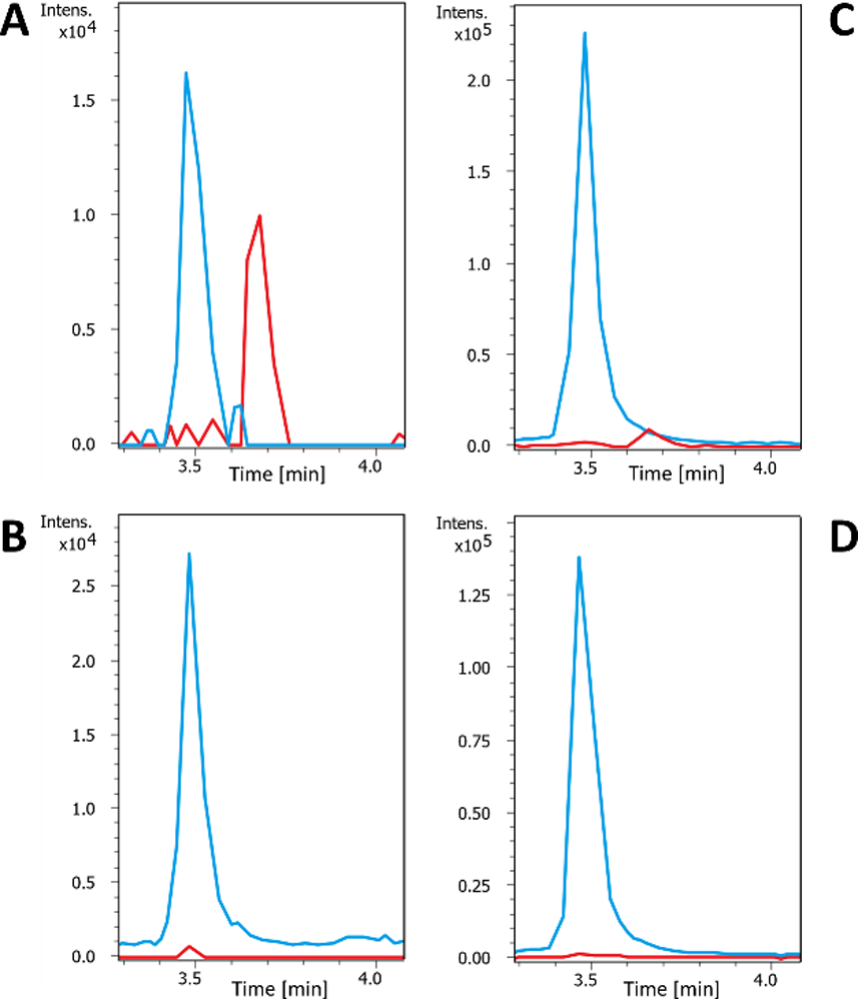
LCMS analysis of the heterologous expression of DAR A F7F and the control strain. Extracted ion chromatograms (EIC, calcd [M + 2H]^2+^ ± 0.01 Da) of DAR A F7F are shown in red (C_47_H_54_N_11_0_12_F; 492.7041 *m*/*z*) and the extracted ion chromatograms (EIC, calcd [M + 2H]^2+^ ± 0.01 Da) of DAR A are shown in blue (C_47_H_55_N_11_0_12_; 483.7089 *m*/*z*). The intensity (Intens.) is plotted on the *y*-axis and the retention time in minutes (min) is plotted on the *x*-axis. Samples are dissolved in 20:80 H_2_O/MeCN + 0.1% FA. (A) Analysis of the heterologous expression of **E. coli** BL21/pEVOL-pylT-N346A/C348A/pJK64 with 4–F-F addition. (B) Analysis of the heterologous expression with **E. coli** BL21/pEVOL-pylT-N346A/C348A/pJK64 without 4–F-F addition. (C) Analysis of the control strain **E. coli** BL21/pEVOL-pylT-N346A/C348A/pZW-ADC9 with 4–F-F addition. (D) Analysis of the control strain **E. coli** BL21/pEVOL-pylT-N346A/C348A/pZW-ADC9 without 4–F-F addition.

Initially, we cultivated the expression strain in the presence of 4-fluoro-l-phenylalanine (4–F-F) and subsequently analyzed the culture extract by UPLC-HRMS. Interestingly, we could detect a clear peak corresponding to the sum formula C_47_H_54_N_11_O_12_F [M+2H]^2+^ with 492.7056 *m*/*z* (calcd 492.7041 *m*/*z*) at a retention time of 3.7 min (Figures 3A, S1 and S2). This peak was not present in samples obtained from cultivations without 4–F-F (Figures 3B and S3). This compound was named darobactin A F7F (DAR A F7F), due to the fluorination of the phenylalanine at position seven (F^7^). In addition, a peak corresponding to the unmodified DAR A could be detected in all cultivations (sum formula C_47_H_55_N_11_0_12_ [M + 2H]^2+^ with 483.7101 *m*/*z* at a retention time of 3.5 min, Figure 3A,B, S3 and S4). When expressing the unmodified minimal BGC, DAR A was produced in the sample with and without 4–F-F, while DAR A F7F could only be detected when the phenylalanine derivative was added (Figures 3C,D, S5 and S6). Although the expression strain was cultivated in minimal medium, **E. coli** is able to synthesize phenylalanine via the shikimate pathway, because it is required for bacterial growth.^20^ The presence of both DAR A and DAR A F7F in the same sample suggests that the natural and the mutant aminoacyl-tRNA synthase (aaRS) accept both, 4–F-F and phenylalanine as substrates. The DAR A F7F to DAR A expression ratio is approximately 1:23 in the strain without modification of *darA* on the plasmid, but 1:1.6 in the strain carrying the plasmid with the amber stop codon mutation. The production rate was observed to shift even further towards the new derivative DAR A F7F (ratio ∼1:0.3) when cultivation was performed in a larger volumes (Figure S7).

### Structural Analysis

**Figure 4:**
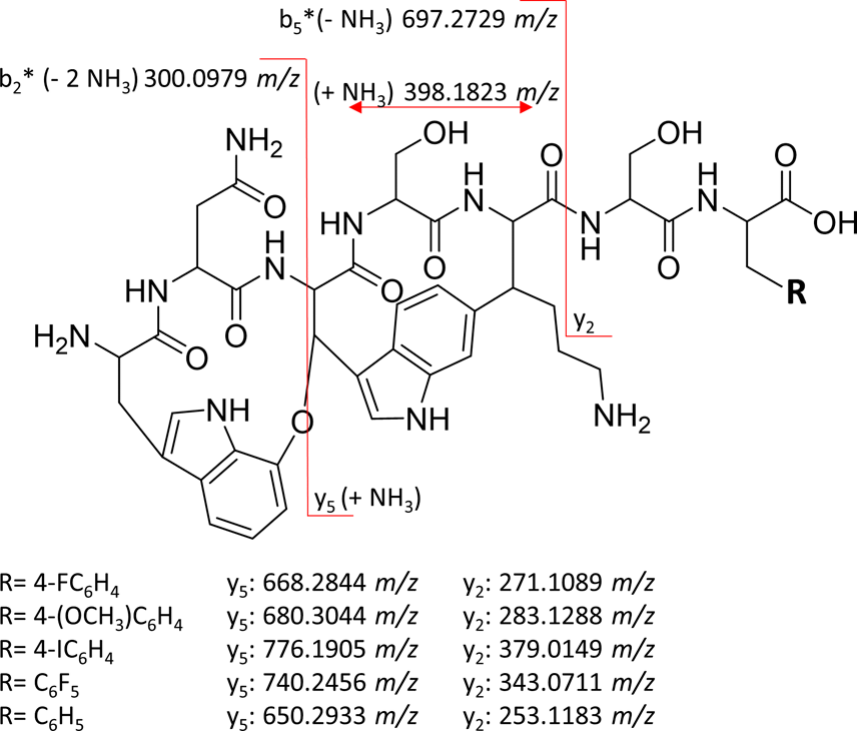
General structure of DAR A derivatives with signature fragment ions detectable in UPLC-HRMS/MS measurements. The mass/charge ratio of the y_2_ and y_5_ fragments depends on the residue −R and therefore on the substituent attached to the phenyl ring of phenylalanine F^7^.

HRMS/MS fragmentation of DAR A F7F revealed the same fragments as DAR A (Figures 4, S8 and S9): N-terminal b_2_* with 300.0979 *m*/*z* (W^1^–N^2^), the fragment containing the carbon–carbon bridge between W^3^ and K^5^ with 398.1823 *m*/*z* (W^3^–S^4^–K^5^), and the b_5_* W^1^–N^2^–W^3^–S^4^–K^5^ with 697.2729 *m*/*z*. For DAR A, C-terminal fragments y_2_ with 253.1183 *m*/*z* (S^6^–F^7^) and y_5_ with 650.2933 *m*/*z* (W^3^–S^4^–K^5^–S^6^–F^7^) were detected. On the other hand, for DAR A F7F, the y_2_ fragment was observed at 271.1086 *m*/*z* (S^6^–F_F_^7^, calcd 271.1089), while the y_5_ fragment was found at 668.2832 *m*/*z* (W^3^–S^4^–K^5^–S^6^–F_F_^7^, calcd 668.2844 *m*/*z*) (Figure 4). The observed mass differences between the two sets of signature fragments are indicative of the fluorine substituent, suggesting the successful heterologous expression of DAR A F7F. To allow further characterization, DAR A F7F was produced and purified on larger scale.

For this purpose, 30 L of culture was fermented and DAR A F7F was purified using an Amberlite XAD-16N column followed by ion exchange chromatography on SP Sepharose XL, flash chromatography, and HPLC, thereupon yielding a total amount of 1.2 mg.

**Figure 5:**
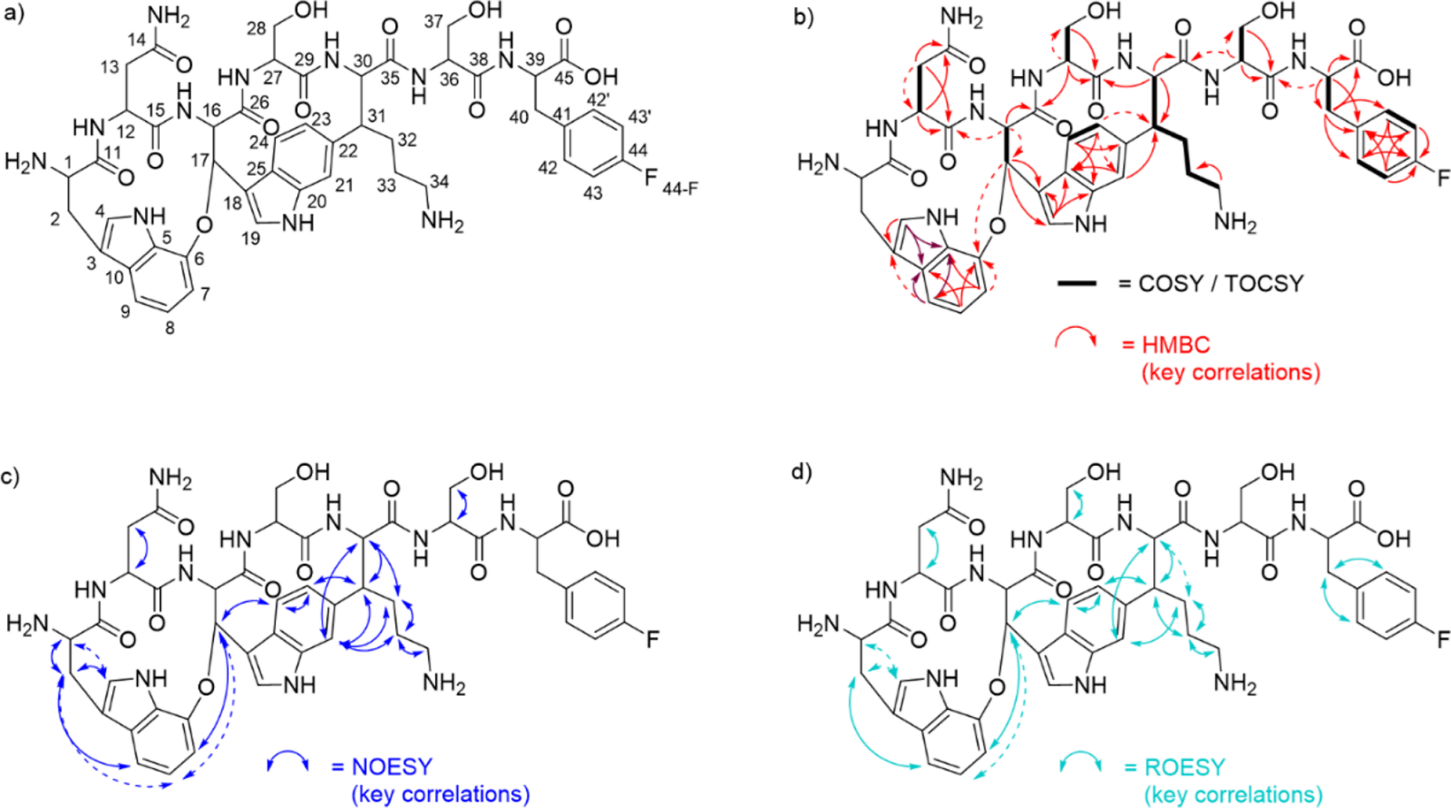
(a) Structure of DAR A F7F, including the atom numbering used for NMR structure elucidation. (b) COSY, TOCSY, and key HMBC correlations of DAR A F7F. (HMBC correlations to C-5 and C-10 could not be distinguished due to the proximity of the respective signals, indicated by dark red arrows.) (c) Key NOESY correlations of DAR A F7F. (d) Key ROESY correlations of DAR A F7F. Dashed arrows indicate weak correlation signals.

Confirmation of the structure hypothesis derived from the MS/MS analysis was established by 1- and 2-dimensional NMR analysis (for key correlations cf. Figure 5b–d). The core structure of DAR A F7F matched well with the reference data of DAR A, as apparent from the comparison of the corresponding ^1^H NMR spectra (Figures S21–S22). Furthermore, HMBC correlations between H-17 and C-6, H-21 and C-31, and H-23 and C-31 confirmed the characteristic ring closures (W^1^–W^3^ C–O–C ether bond and W^3^–K^5^ C–C bond) of the darobactin scaffold. The incorporation of 4–F-F at the C-terminal end of the molecule was verified by the HMBC correlation between H-39 and C-38. As expected, the 4–F-F residue shows additional line splitting due to coupling of the fluorine nucleus with protons H-42,42’ and H-43,43’ as well as with carbon atoms C-42,42’, C-43,43’, and C-44. Consequently, for each of the aforementioned carbon atoms, a doublet signal is detected. The observed ^13^C chemical shifts as well as the values of the C–F coupling constants (C-42,42’: 131.1 ppm, ^3^*J*_C,F_ = 8.2 Hz; C-43,43’: 115.1 ppm, ^2^*J*_C,F_ = 21.3 Hz; C-44:161.7 ppm, ^1^*J*_C,F_ = 241.5 Hz) are typical for monofluorinated aromatic rings and match well with literature data.^21−23^ Likewise, the observed ^19^F NMR shift of −117.94 ppm is in good agreement with the value reported for 4–F-F.^24^ The multiplicity observed in the ^19^F NMR spectrum also shows resemblance to literature data for 4–F-F,^25^ even though the resolution is not sufficient to determine coupling constants.

### Activity Assay

The antimicrobial activity of purified DAR A F7F was investigated by determination of the minimum inhibitory concentrations (MIC) against a panel of Gram-negative and Gram-positive control strains (Tables 1 and S3). In general, the potency and the antimicrobial spectrum of DAR A F7F are similar to the parent compound DAR A, while DAR B showed stronger activity against **E. coli**, **K. pneumoniae*,* and **P. aeruginosa** strains. Initially, we tested the new compound against **E. coli** MG1655 bamA6ΔbamB, an engineered strain which is highly susceptible to BamA inhibitors,^26^ and detected intriguing activity (0.125 μg/mL). In further assays, DAR A F7F inhibited the growth of **K. pneumoniae** and **E. coli** strains at 8–4 μg/mL (including mcr-1 positive clinical isolate NRZ14408). The potency is comparable to the parent compound DAR A (one dilution step difference), while the reference compound DAR B inhibited the same **E. coli** and **K. pneumoniae** strains at 2–0.5 μg/mL. We could not observe any meaningful growth inhibitory effects of DAR A F7F and DAR A against **P. aeruginosa** ATCC27853, PA103, and the clinical isolate **P. aeruginosa** EXT111762. In contrast, the moderately virulent laboratory strain PAO1^27^ and the efflux deficient variant PAO750^28^ were inhibited by the new and the parent compound at 8 μg/mL (DAR A F7F) and 4–2 μg/mL (DAR A). Similarly, the activity of DAR A F7F was identical against **E. coli** ATCC25922 and the ΔTolC mutant (8–4 μg/mL), indicating that drug-efflux might not be of primary concern during further optimization of darobactin-type compounds. No activity was observed against *S. aureus* and **A. baumannii** ATCC19606 in the selected concentration range.

**Table 1:** MIC values of DAR A, DAR B and DAR A F7F (F7F) against **Escherichia coli** (Ec), **Pseudomonas aeruginosa** (Pa), **Klebsiella pneumoniae** (Kp), **Acinetobacter baumannii** (Ab), and *Staphylococcus aureus* (Sa)^a^

		MIC μg/mL		
organism and test strain		DAR B	DAR A	F7F
*Ec*	MG1655 bamA6ΔbamB	n.d.	<0.03	0.125
	ATCC25922	1–0.5	n.d.	8–4
	ATCC25922 ΔTolC	0.5–0.25	n.d.	8–4
	NRZ14408 mcr-1	1–0.5^7^	4^7^	8
*Pa*	PAO1	2–1^9^	4–2^7^	8
	PAO750	0.5	4	8
	PA103	8^11^	n.d.	64
	ATCC27853	8 ^9^	>64	>64
	EXT111762	2–1^9^	>64	64
*Ab*	ATCC19606	32^7^	>64	>64
*Kp*	DSM30104	2–1^7^	4	8
	ATCC700603	2–1^11^	n.d.	16–8
*Sa*	ATCC33592	>64	>64	>64

a Experiments were done in triplicate, and values are given in μg/mL.

### Heterologous Expression with Further Phenylalanine Derivatives

Next, we investigated whether further novel DAR A derivatives can be produced using the amber stop codon technique. Hence, the heterologous production strain was cultivated in minimal media supplemented with different phenylalanine analogues, for which the literature indicated good integration efficiencies. Although it has to be kept in mind that variations in the literature data can be observed and that Young *et al*. used a different OTS.^19,29^ We decided to use 4-chloro-l-phenylalanine (4–Cl-F), 4-trifluoromethyl-l-phenylalanine (4-CF_3_–F), 4-azido-l-phenylalanine (4-N_3_–F), 4-nitro-l-phenylalanine (4-NO_2_–F), 4-methoxy-l-phenylalanine (4-OMe-F), 4-bromo-l-phenylalanine (4–Br-F), 4-iodo-l-phenylalanine (4–I-F), 3,4-dihydroxy-l-phenylalanine (3,4-OH-F) and 2,3,4,5,6-pentafluoro-l-phenylalanine (F_5_–F) as respective supplements.

**Figure 6:**
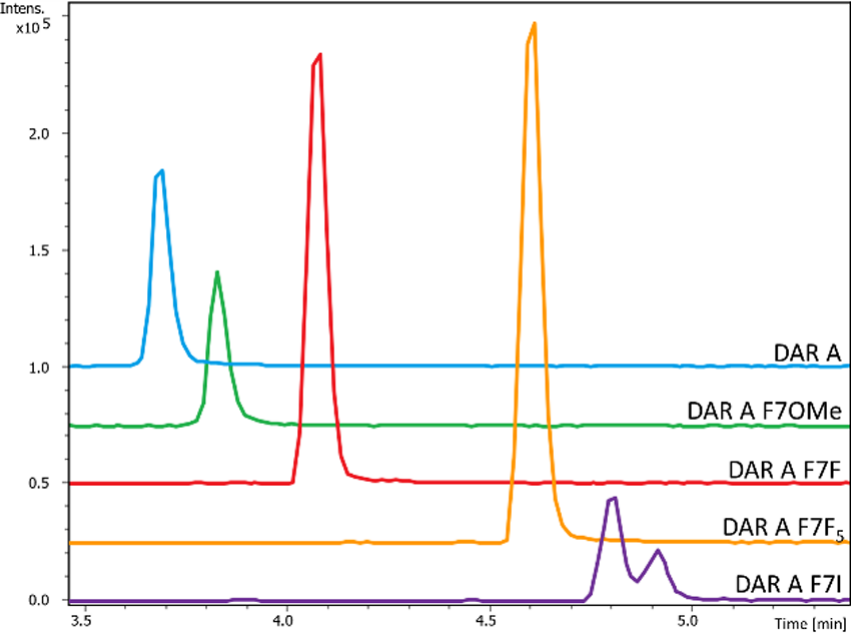
Extracted ion chromatograms (EIC, calcd [M + 2H]^2+^ ± 0.01 Da) of DAR A F7I in purple (C_47_H_54_N_11_O_12_I; 546.6572 *m*/*z*), DAR A F7F_5_ in yellow (C_47_H_50_N_11_O_12_F_5_; 528.6853 *m*/*z*), DAR A F7F in red (C_47_H_54_N_11_O_12_F; 492.7041 *m*/*z*), DAR A F7OMe in green (C_48_H_57_N_11_O_13_; 498.7141 *m*/*z*) and DAR A in blue (C_47_H_55_N_11_O_12_; 483.7089 *m*/*z*) from the heterologous expression of **E. coli** BL21/pEVOL-pylT-N346A/C348A/pJK64 with the respective non-canonical phenylalanine derivatives. Samples were dissolved in 50:50 H_2_O/MeOH. The intensity (Intens.) is plotted on the *y*-axis and the retention time in minutes (min) on the *x*-axis.

In samples generated by heterologous expression in the presence of 4-OMe-F, 4–I–F, and F_5_–F (Figure 2), we detected by UPLC-HRMS peaks matching the expected mass to charge ratios of the respective DAR A derivatives at retention times between 3.7 and 4.9 min (Figure 6). In all samples, the characteristic fragments of DAR A were detected, except fragment b_5_*, which was only present for the DAR A F7F and darobactin A F7F_5_ (DAR A F7F_5_) derivatives. However, due to the modification of the phenylalanine in position seven, fragments y_2_ and y_5_ differ from the fragmentation signature of the native compound. When cultivating in the presence of 4-OMe-F the signals at 283.1296 *m*/*z* and 680.3050 *m*/*z* correspond to the calculated y_2_ and y_5_ fragments of darobactin A F7OMe (DAR A F7OMe) of calculated 283.1288 *m*/*z* (y_2_, S^6^–F_OMe_^7^) and 680.3044 *m*/*z* (y_5,_ W^3^–S^4^–K^5^–S^6^–F_OMe_^7^), indicating the successful heterologous expression of this derivative (Figures 4 and S12–S14). Similar results were achieved when cultivating in the presence of 4–I-F. Mass to charge ratios of 379.0146 *m*/*z* and 776.1906 *m*/*z* corresponding to the y_2_ (S^6^–F_I_^7^, calcd 379.0149 *m*/*z*) and y_5_ (W^3^–S^4^–K^5^–S^6^–F_I_^7^, calcd 776.1905 *m*/*z*) fragments of darobactin A F7I (DAR A F7I) could be detected (Figure 4 and S15–S17). For the heterologous expression with F_5_–F, the y_2_ fragment of 343.0720 *m*/*z* (S^6^–F_F5_^7^, calcd 343.0711 *m*/*z*) and the y_5_ fragment of 740.2463 *m*/*z* (W^3^–S^4^–K^5^–S^6^–F_F5_^7^, calcd 740.2456 *m*/*z*) were present, demonstrating the production of DAR A F7F_5_ (Figure 4 and S18–S20).

The heterologous expression supplemented with either 4–Cl-F or 4-CF_3_–F, 4-N_3_–F, 4-NO_2_–F, 4–Br–F, or 3,4-OH-F did not lead to the detection of the expected derivative. Also, the newly engineered DAR A derivatives exhibit varying production levels (Figure 6). One reason could be different integration rates of the phenylalanine derivatives, which was already observed for other RiPPs in the literature as well as shown for DAR A in this study. For example, only for Wang *et al*. postulated low yields of brominated phenylalanine is integration, while other studies indicated good efficiencies.^19,29,30^ In our study described herein, the attempts to include 4-BR-F into DAR A were unscuccessful.^19^ This indicates that integration efficiencies may be specific for each RiPP product. Another possibility could be a reduced interaction rate of the altered linear heptapeptide with the rSAM enzyme to introduce the two rings.^12,19,29^ The linear heptapeptides might have been produced but subsequently degraded by unspecific proteases, because the intramolecular ring closures are not introduced to stabilize the compound. Since the mechanism of how the rings are introduced is not understood in detail, it can only be hypothesized that some of the newly introduced phenylalanine residues might have disturbed interactions with the enzyme.^12^

In this study, we successfully adapted the amber stop codon suppression for targeted introduction of ncAAs in DAR A. We were able to detect four new derivatives, DAR A F7F, DAR A F7OMe, DAR A F7I, and DAR A F7F_5_. The first one, DAR A F7F, was isolated and characterized by NMR. In MIC studies, the compound showed similar activities compared to DAR A. In general, the method developed in this study provides a solid tool for the biotechnological modification of darobactins.

## Material and Methods

### Plasmid Construction

The plasmids were constructed by a self-prepared isothermal assembly reaction.^31^ The plasmid pJK63, carrying the DAR A precursor peptide, the gene for the rSAM enzyme, both from *Photorhabdus khanii* DSM3369, and the gene for the tRNA with the CUA anticodon,^19^ were generated from pZW-ADC9^13^ and pEVOL-pylT-N346A/C348A.^19^ The expression vector pJK64 was cloned by amplifying pJK63 to exchange the phenylalanine triplet of the core peptide with the triplet TAG. Primer sequences and further information are provided in Tables S1 and S2. To generate the production strain, **E. coli** BL21 was transformed with the plasmids pJK64 and pEVOL-pylT-N346A/C348A.^31^

### Heterologous Expression of the DAR A Derivatives

For the heterologous expression of target derivatives, a preculture of the production strain **E. coli** BL21+ pJK64+ pEVOL-pylT-N346A/C348A was prepared in LB_Kan+Ca_ and used to inoculate DAR49*_Kan+Ca_. The culture was incubated at 37 °C and 200 rpm until an optical density (OD_600_) of 1.0, cooled for 15 min at 4 °C and the production was induced by adding 1 mM IPTG (isopropyl-β-d-thiogalactopyranoside), 0.2% arabinose, and 2 mM of the respective phenylalanine analogue (dissolved in 1 M NaOH just before use). Incubation was performed for 3 days at 30 °C and 200 rpm.

### UPLC-HRMS and HRMS/MS Sample Preparation and Measurement

UPLC-HRMS samples were prepared by using C_18_ stage tips or C_18_ SPE columns. For the C_18_ stage tip purification, the column was prepared as described previously.^32^ The stage tip was washed with 200 mL of 100% MeOH and afterward equilibrated using 200 mL of 95:5 H_2_O/acetonitrile (MeCN) + 0.1% formic acid (FA) by centrifugation at 3000 rpm and 4 °C for ∼4 min. Cleared supernatant of the cultivation (400–500 μL) was applied to the stage tip by repeating the centrifugation step twice. To remove salts, the C_18_ matrix was washed with 30 mL 95:5 H_2_O/MeCN + 0.1% FA. Afterward, the sample was eluted twice using 30 mL 20:80 H_2_O/MeCN + 0.1% FA for each elution. For the C_18_ SPE extraction, a SPE column, CHROMABOND C18 ec f, 100 μm, 6 mL/1000 mg (Macherey- Nagel, Düren, Germany) was washed twice with 6 mL MeOH and afterward twice with 6 mL ddH_2_O. The column was loaded with 50 mL culture supernatant (1 mL/min) and subsequently washed with 6 mL ddH_2_O. Afterward, the column was left under vacuum for 15 min to dry completely. The product was eluted with three times 6 mL 50:50 H_2_O/MeOH + 0.1% FA, followed by 6 mL MeOH + 0.1% FA, three times 6 mL 20:80 H_2_O/MeCN + 0.1% FA, and 6 mL MeCN + 0.1% FA. The samples were dried and redissolved in 100 μL 50:50 H_2_O/MeOH. UPLC-HRMS measurements were carried out with an Agilent Infinity 1290 UPLC system coupled to a DAD detector and a micrOTOFQ II mass spectrometer (Bruker Daltonics, Bremen, Germany) with an electrospray ionization source. Measurements with high accuracy were done on a UPLC system of the same type coupled to DAD and ELSD detectors and a maXis II ESI-qTOF-UHRMS (Bruker Daltonics, Bremen, Germany). In both cases, the stationary phase of the UPLC system was an Acquity UPLC BEH C18, 130 Å, 1.7 μm (2.1 mm × 100 mm) column and an Acquity UPLC BEH C18, 130 Å, 1.7 μm VanGuard Pre-Column (2.1 mm × 5 mm; Waters, Eschborn, Germany). The following gradient was used for the LC system coupled to micrOTOFQ II: 0 min: 95% A; 0.80 min: 95% A; 18.70 min: 4.75% A; 18.80 min: 0% A; 23.00 min: 0% A; 23.10 min: 95% A; 25.00 min: 95% A (A: H_2_O, 0.1% FA; B: MeCN, 0.1% FA). For the LC system coupled to the maXis II ESI-qTOF-UHRMS instrument, a gradient of 0 min: 95% A; 0.30 min: 95% A; 18.00 min: 4.75% A; 18.10 min: 0% A; 22.50 min: 0% A; 22.60 min: 95% A; 25.00 min: 95% A (A: H_2_O, 0.1% FA; B: MeCN, 0.1% FA) was used. A flow rate of 600 μL/min and a column oven temperature of 45 °C were used for both systems. The internal standard for mass spectra calibration was a 10 mM sodium formate solution in H_2_O/*^i^*PrOH (1:1). A sample volume of 5 μL was injected. For MS/MS fragmentation analysis, auto-MSMS settings were used. The recorded spectra were analyzed using Compass DataAnalysis version 4.2 and 5.3 (Bruker).

### Purification of the DAR A F7F

DAR A F7F was isolated according to a modified protocol from Imai et al.^1^*E. coli* BL21+ pJK64 + pEVOL-pylT-N346A/C348A was cultivated as described above, and the culture broth was lyophilized after 3 days of cultivation. The compound was extracted from the lyophilized culture broth using 50:50 H_2_O/MeOH. The concentrated extract was incubated with 10% v/v Amberlite XAD-16N resin (20–60 mesh, Sigma Life Science [Merck KGaA], Darmstadt, Germany) and the resin was subsequently washed with two column volumes (CV) of H_2_O + 0.1% FA. The compound was eluted with 3 CV 50:50 H_2_O/MeOH + 0.1% FA, and the MeOH was removed using a rotary evaporator. Afterward, the elution fraction was loaded onto a SP Sepharose XL strong ion exchange column (220 mL bed volume, GE Healthcare, Uppsala, Sweden) and washed with 10 CV H_2_O+ 0.1% FA. Elution was done with 50 mM NH_4_OAc pH adjusted to 5, 7, 9, and 11 using 10 CV each. The compound containing elution fraction with pH 7 was further purified by reversed-phase flash chromatography using a Puriflash 4125 chromatography system (Interchim, Montlucon, France) with a puriFlash C18-AQ 30 μm F0120 flash column (Interchim) as follows: 0–20 min: 5% D; 20–60 min: 5–100% D; 60–90 min: 100% D (C: H_2_O, 0.1% FA, D: MeOH, 0.1% FA), with a flow rate of 30 mL/min. DAR A F7F containing fractions were combined and dried in a GeneVac centrifugal concentrator (SPScientific, Ipswich, UK). Dry weight was determined, and fractions were subjected to HPLC (Shimadzu Deutschland GmbH, Duisburg, Germany) purification with a NUCLEODUR C18 Gravity-SB 3 μm (Macherey-Nagel, Düren, Germany) using a gradient:

0–5 min: 5% B; 5–7 min: 5–25% B; 7–27 min: 25–50% B; 27–30 min: 50–100% B (A: H_2_O, 0.1% FA; B: MeCN, 0.1% FA). The flow rate was set to 2 mL/min, and the column oven temperature was maintained at 40 °C.

### NMR Spectroscopy

NMR spectra (^1^H, ^13^C, ^19^F, COSY, ^1^H,^1^H-TOCSY, NOESY, ROESY, HSQC, HSQC-TOCSY, and HMBC) were recorded at 298 K on an Avance Neo 700 MHz spectrometer (^1^H: 700.28 MHz, ^13^C: 176.09 MHz, ^19^F: 658.92 MHz; Bruker BioSpin GmbH, Rheinstetten, Germany) equipped with a 5 mm CryoProbe Prodigy TCI (^1^H, ^15^N, ^13^C Z-GRD). All measurements were carried out using D_2_O as a solvent. Chemical shifts are given in ppm. ^1^H spectra were referenced to the residual solvent signal (δ = 4.79 ppm).^33^ For ^13^C measurements, 3-(trimethylsilyl)propionic-2,2,3,3-d_4_ acid sodium salt was used as the external standard. For ^19^F measurements, α,α,α-trifluorotoluene served as an external standard. For a better resolution of the correlation signals, HSQC and HMBC spectra were acquired using nonuniform sampling (NUS). HSQC-TOCSY, ^1^H,^1^H-TOCSY, NOESY, and ROESY spectra were measured with H_2_O suppression. For ^1^H, ^13^C DEPTQ-135, ^19^F, COSY, HSQC (with NUS sampling), and HMBC experiments, a concentrated sample of DAR A F7F was used, while HSQC (without NUS sampling), HSQC-TOCSY, ^1^H,^1^H-TOCSY, NOESY, and ROESY experiments were acquired for a dilute sample. Analysis of NMR spectra was achieved using TopSpin 3.6.0 (Bruker BioSpin GmbH, Rheinstetten, Germany).

### Antimicrobial Activity of DAR A F7F

The antimicrobial activity of DAR A F7F was interrogated by determining the minimum inhibitory concentration (MIC) against the following test panel: *E. coli* MG1655 bamA6_bamB, *E. coli* ATCC35218, *E. coli* ATCC25922, *E. coli* ATCC25922 ΔTolC, *E. coli* NRZ14408 mcr-1, *Pseudomonas aeruginosa* PAO1, , *P. aeruginosa* PAO750, *P. aeruginosa* PA103, *P. aeruginosa* ATCC27853, *P. aeruginosa* EXT111762, *A. baumannii* ATCC19606, *K. pneumoniae* DSM30104, *K. pneumoniae* ATCC700603, and *S. aureus* ATCC33592. The antibiogram and the resistance determinants of the clinical isolates *E. coli* NRZ14408 and *P. aeruginosa* EXT111762 can be found in our previous studies.^10,11^

Briefly, the assays were conducted by using the microbroth dilution method in round-bottom 96-well plates. Overnight cultures of the test strains were adjusted to McFarland Standard of 1.0 and subsequently diluted to 5× 10^5^ cells mL^–1^ in cation-adjusted Mueller Hinton 2 broth. Darobactins were screened in 12 concentrations ranging from 64 to 0.03 μg mL^–1^ in triplicate. Ceftazidime, ciprofloxacin, and gentamicin were used as standards on each assay plate. Bacterial suspension without supplemented standard antibiotics or darobactin was used as a negative control. After incubation (18 h, 180 rpm, 37 °C, 85% relative humidity), cell growth was determined by measuring the turbidity using a microplate spectrophotometer at 600 nm. The MIC was defined as the minimum concentration at which at least 85% growth inhibition relative to the negative control was measured. Relative inhibition (h) was calculated according to *h* % = 100 * [1 – (AU sample – AU Low)/(AU High – AU Low)]. AU: absorption unit; Low: medium blank; High: negative control of maximal growth. Results were compared to reference darobactins and are summarized in Table S3.

## Supporting Information Available

Primer sequences, plasmid descriptions, antimicrobial activity assay data, and UPLC-HRMS/MS and NMR data (PDF)
