# Supplementary material for: Expanding the Range of Darobactin Derivatives by Amber Stop Codon Suppression To Introduce Non-canonical Amino Acids
Source: ACS Omega. 2025 Apr 30;10(18):18356–63. doi: 10.1021/acsomega.4c10307 (PMC12079272; doi:10.1021/acsomega.4c10307)
Supplement: Supplementary file 1 — ao4c10307_si_001.pdf [file ao4c10307_si_001.pdf]

SI

Expanding the range of darobactin derivatives by amber stop codon suppression to introduce non-canonical amino acids

Jil-Christine Kramer [1], Zerlina G. Wuisan [1], Ute Mettal [1], Michael Marner [1,2], Till F. Schäberle [1, 2, 3*]

[1]Institute for Insect Biotechnology with Focus on Natural Product Research; Justus-Liebig-University Giessen, Ohlebergsweg 12, 35392, Giessen, Germany

[2]Natural Product Department; Fraunhofer-Institute for Molecular Biology and Applied Ecology (IME), Ohlebergsweg 12, 35392 Giessen, Germany

[3]German Center for Infection Research (DZIF); Partner Site Giessen-Marburg-Langen, Ohlebergsweg 12, 35392 Giessen, Germany

[*] Correspondence: Till.F.Schaeberle@agrar.uni-giessen.de (T. F. S.)

Table of content

Plasmids and primers 3

LCMS spectra of darobactin A F7F and darobactin A 3

UV chromatograms of darobactin A F7F 9

LCMS spectra of darobactin A F7OMe and darobactin A 10

LCMS spectra of darobactin A F7I and darobactin A 12

LCMS spectra of darobactin A F7F_5 and darobactin A 14

In vitro activity 16

NMR data of darobactin A F7F 17

Plasmid sequences 75

References 79

Plasmids and primers

Table S1: Primers used in this study.

Primer Name	Sequence 5'-3'
**JCK223_pylT_Gib_adc9_fw**	CTTTAATAAGGAGATATACCCCGTGTGCTTCTCAAATGCCTG
**JCK224_pylT_Gib_adc9_rv**	TTTCTGTTCGACTTAAGCACATGCAAAAAAGCCTGCTCGTTG
**JCK225_adc9_pylT_Gib_fw**	AGGCTTTTTTGCATGTGCTTAAGTCGAACAGAAA
**JCK226_adc9_pylT_Gib_rv**	TGAGAAGCACACGGGGTATATCTCCTTATTAAAG
**JCK217_TAG_adc9_fw**	GGTCAAAAAGCTAGCAGGAAATTTAAAGCTTATC
**JCK218_TAG_adc9_rv**	TTAAATTTCCTGCTAGCTTTTTGACCAGTTCCAGG

Table S2: Plasmids used in this study.

Plasmid Name	Genes	Origin
**pZW-ADC9**	pRSF.deut with $darA_{Pho}$ and $darE_{Pho}$	Wuisan et al.[1]
**pEVOL-pylT-N346A/C348A**	Pyrrolysyl-tRNA synthetase/pyrrolysyl -tRNA pair	Wang et al. [2]<br>Addgene #127411
**pJK63**	pZW-ADC9 with pyrrolysyl -tRNA	This Study
**pJK64**	pJK63 with TAG codon exchange	This Study

LCMS spectra of darobactin A F7F and darobactin A

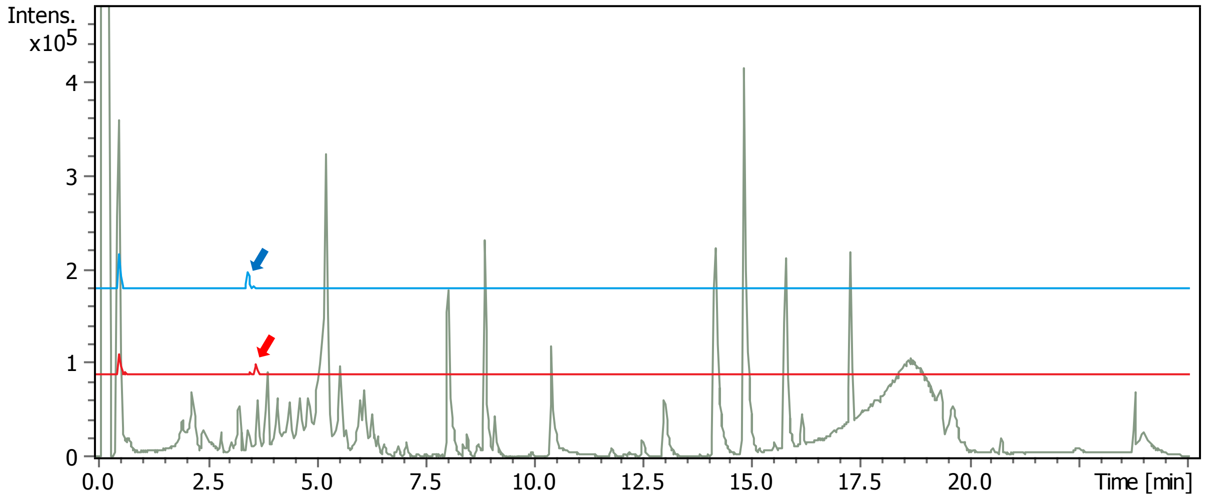

Figure S1: Extracted ion chromatogram (EIC, calcd. $[M + 2H]^{2+}$ ±0.01 Da) of darobactin A F7F in red ($C_{47}H_{54}N_{11}O_{12}F$; 492.7041 *m/z*), extracted ion chromatogram (EIC, calcd. $[M + 2H]^{2+}$ ±0.01 Da) of darobactin A in blue ($C_{47}H_{55}N_{11}O_{12}$; 483.7089 *m/z*) and the base peak chromatogram (BPC) in grey of the heterologous expression of darobactin A F7F by *E. coli* BL21/ pEVOL-pylT-N346A/C348A/ pJK64. Samples were dissolved in 20:80 H_2O/MeCN + 0.1% FA. The red arrow indicates darobactin A F7F at a retention time of 3.7 min and the blue arrow darobactin A at a retention time of 3.5 min.

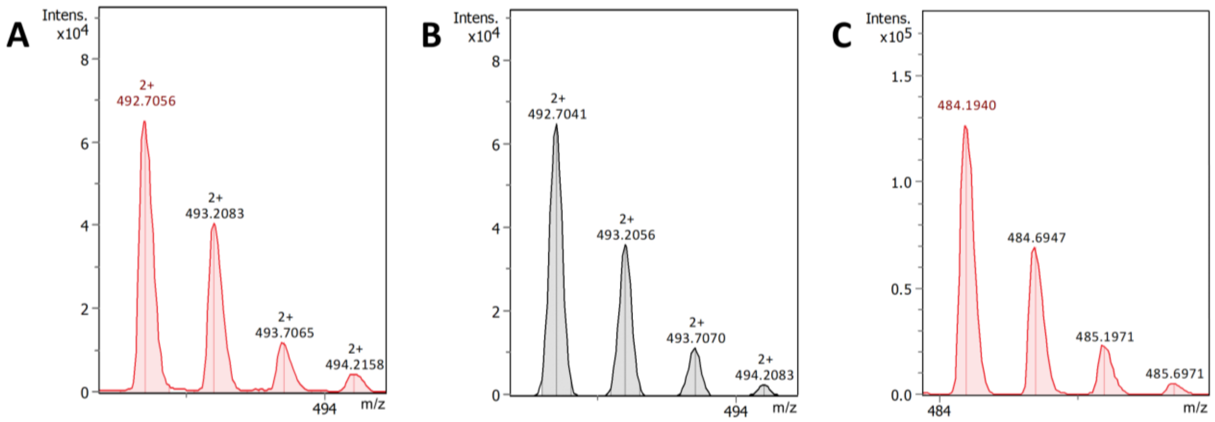

Figure S2: Recorded mass spectrum of **A:** darobactin A F7F ($C_{47}H_{54}N_{11}O_{12}F$) $[M+2H]^{2+}$, **B:** simulated mass spectrum of darobactin A F7F ($C_{47}H_{54}N_{11}O_{12}F$) $[M+2H]^{2+}$ calculated by Compass DataAnalysis and recorded mass spectrum of **C:** darobactin A F7F ($C_{47}H_{54}N_{11}O_{12}F$) $[M-NH_3+2H]^{2+}$.

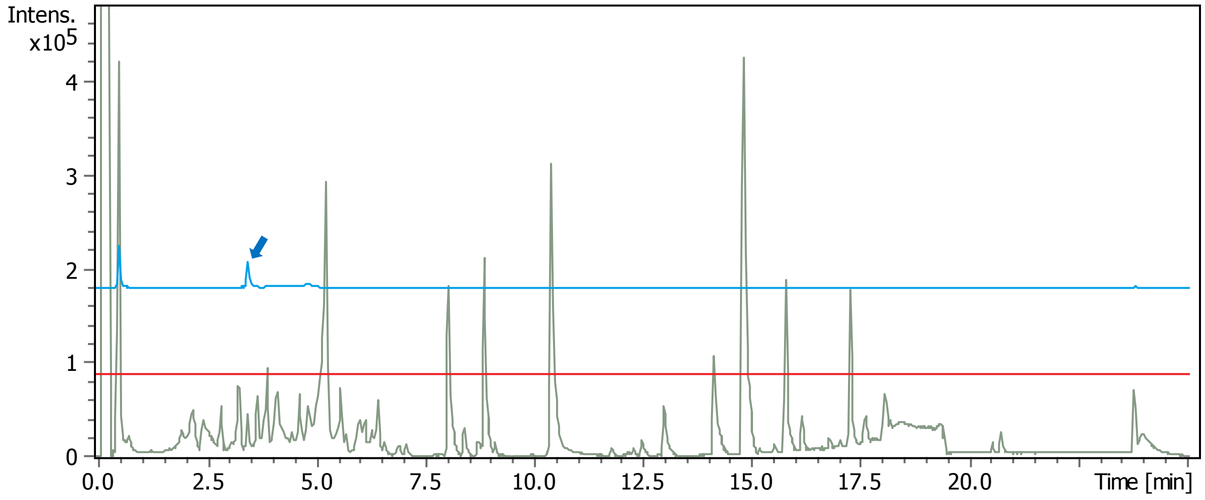

Figure S3: Extracted ion chromatogram (EIC, calcd. $[M + 2H]^{2+}$ ±0.01 Da) of darobactin A F7F in red ($C_{47}H_{54}N_{11}O_{12}F$; 492.7041 *m/z*), extracted ion chromatogram (EIC, calcd. $[M + 2H]^{2+}$ ±0.01 Da) of darobactin A in blue ($C_{47}H_{55}N_{11}O_{12}$; 483.7089 *m/z*) and the base peak chromatogram (BPC) in grey of the heterologous expression of darobactin A F7F by *E. coli* BL21/ pEVOL-pylT-N346A/C348A/ pJK64 without adding 4-fluoro-L-phenylalanine. Samples were dissolved in 20:80 H_2O/MeCN + 0.1% FA. The blue arrow indicates darobactin A at a retention time of 3.5 min.

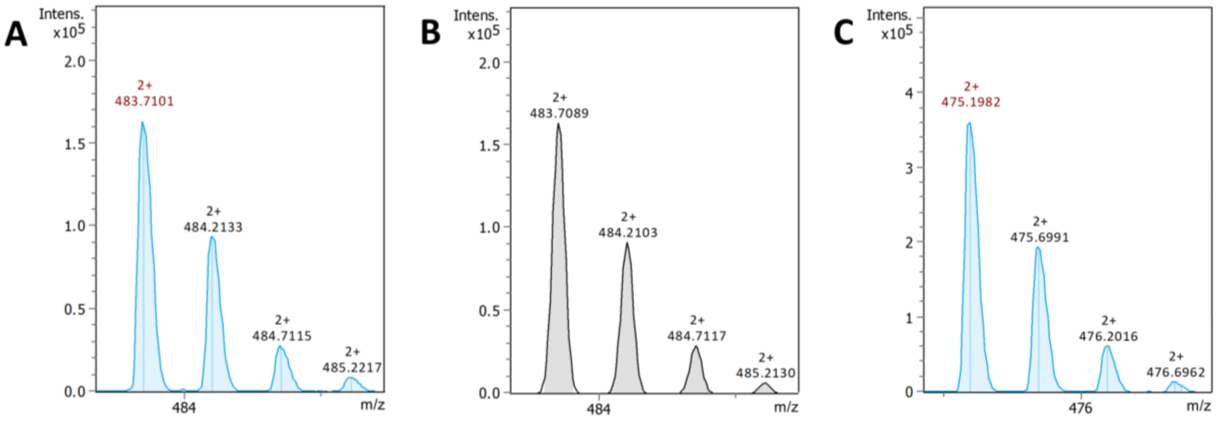

Figure S4: Recorded mass spectrum of **A:** darobactin A ($C_{47}H_{55}N_{11}O_{12}$) $[M+2H]^{2+}$, **B:** simulated mass spectrum of darobactin A ($C_{47}H_{55}N_{11}O_{12}$) $[M+2H]^{2+}$ calculated by Compass DataAnalysis and recorded mass spectrum of **C:** darobactin A ($C_{47}H_{55}N_{11}O_{12}$) $[M-NH_3+2H]^{2+}$.

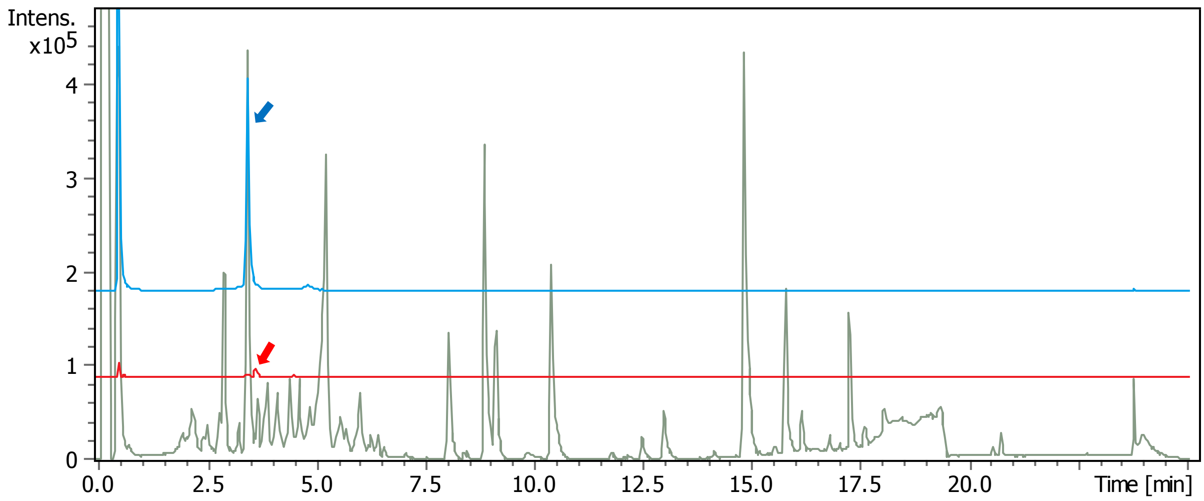

Figure S5: Extracted ion chromatogram (EIC, calcd. $[M + 2H]^{2+}$ ±0.01 Da) of darobactin A F7F in red ($C_{47}H_{54}N_{11}O_{12}F$; 492.7041 *m/z*), extracted ion chromatogram (EIC, calcd. $[M + 2H]^{2+}$ ±0.01 Da) of darobactin A in blue ($C_{47}H_{55}N_{11}O_{12}$; 483.7089 *m/z*) and the base peak chromatogram (BPC) in grey of the control from the heterologous expression of darobactin A F7F by *E. coli* BL21/ pEVOL-pylT-N346A/C348A/ pZW-ADC9. Samples were dissolved in 20:80 H_2O/MeCN + 0.1% FA. The blue arrow indicates darobactin A at a retention time of 3.5 min and the red arrow indicates darobactin A F7F at a retention time of 3.7 min.

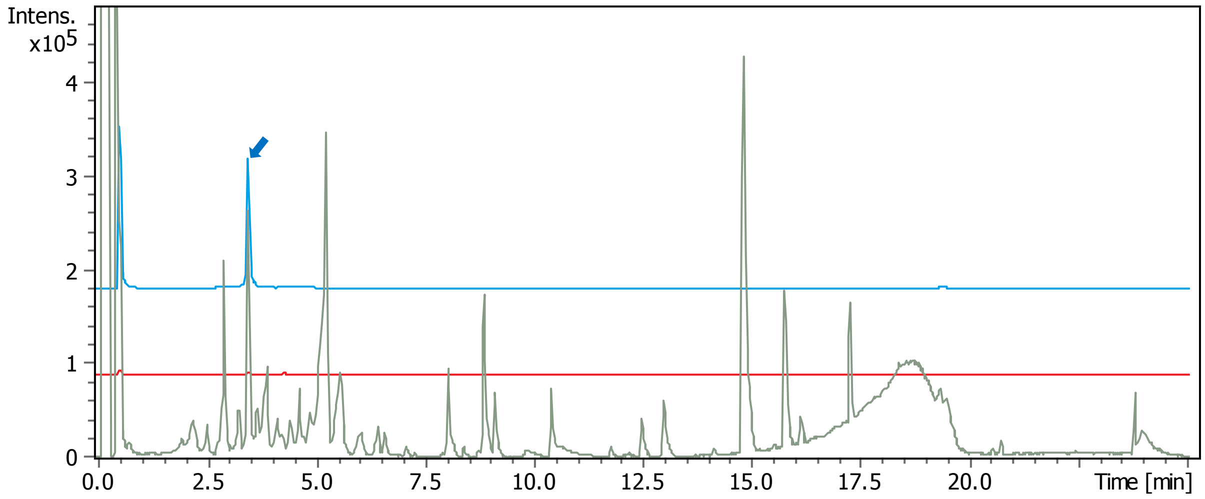

Figure S6: Extracted ion chromatogram (EIC, calcd. $[M + 2H]^{2+}$ ±0.01 Da) of darobactin A F7F in red ($C_{47}H_{54}N_{11}O_{12}F$; 492.7041 *m/z*), extracted ion chromatogram (EIC, calcd. $[M + 2H]^{2+}$ ±0.01 Da) of darobactin A in blue ($C_{47}H_{55}N_{11}O_{12}$; 483.7089 *m/z*) and the base peak chromatogram (BPC) in grey of the control from the heterologous expression of darobactin A F7F by *E. coli* BL21/ pEVOL-pylT-N346A/C348A/ pZW-ADC9 without 4-fluoro-L-phenylalanine. Samples were dissolved in 20:80 H_2O/MeCN + 0.1% FA. The blue arrow indicates darobactin A at a retention time of 3.5 min.

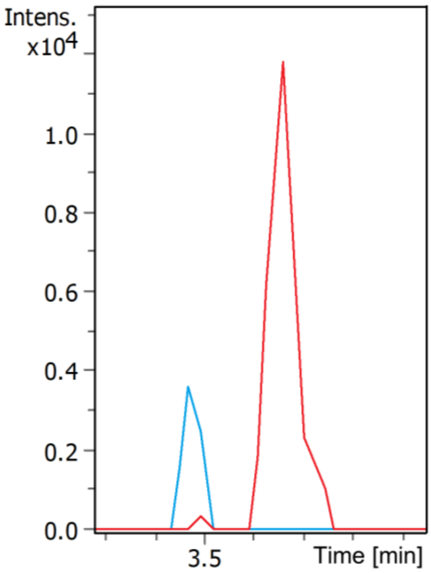

Figure S7: Extracted ion chromatogram (EIC, calcd. $[M + 2H]^{2+}$ ±0.01 Da) of darobactin A F7F in red ($C_{47}H_{54}N_{11}O_{12}F$; 492.7041 *m/z*) and extracted ion chromatogram (EIC, calcd. $[M + 2H]^{2+}$ ±0.01 Da) of darobactin A in blue ($C_{47}H_{55}N_{11}O_{12}$; 483.7089 *m/z*) of the heterologous expression of darobactin A F7F by *E. coli* BL21/ pEVOL-pylT-N346A/C348A/ pJK64 in 1 L medium. Samples were dissolved in 20:80 H_2O/MeCN + 0.1% FA.

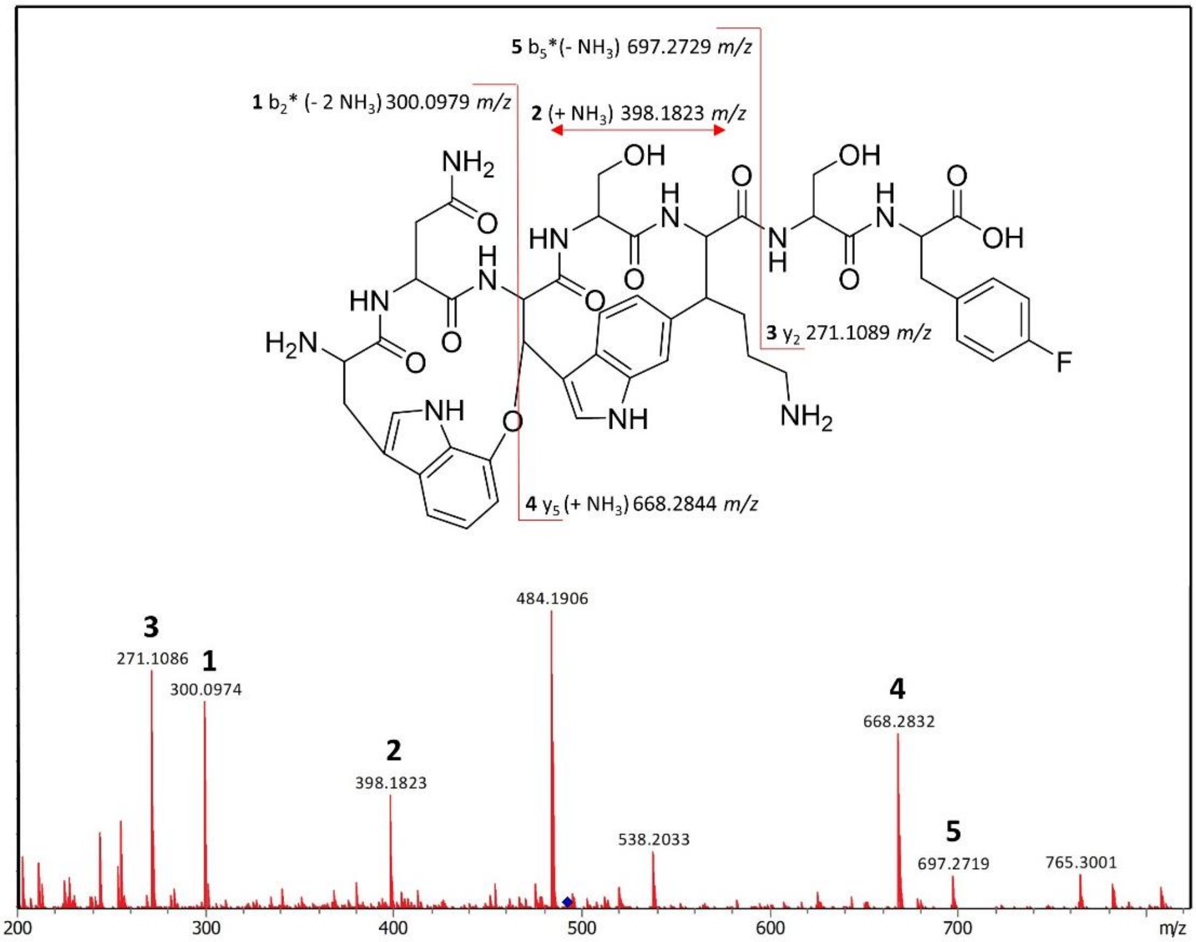

Figure S8: MS/MS spectrum and fragmentation pattern of darobactin A F7F. Identified fragments are numbered accordingly.

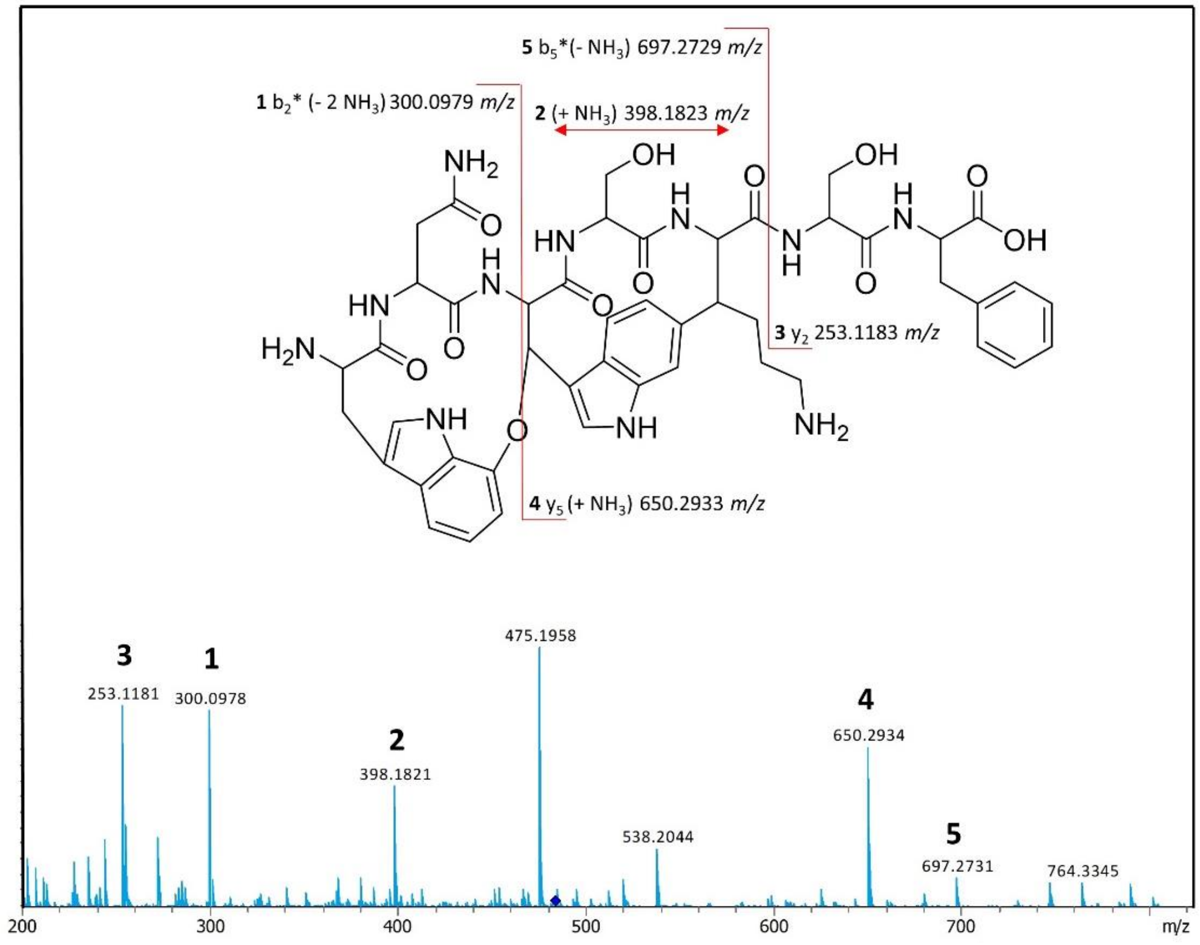

Figure S9: MS/MS spectrum and fragmentation pattern of darobactin A. Identified fragments are numbered accordingly.

UV chromatograms of darobactin A F7F

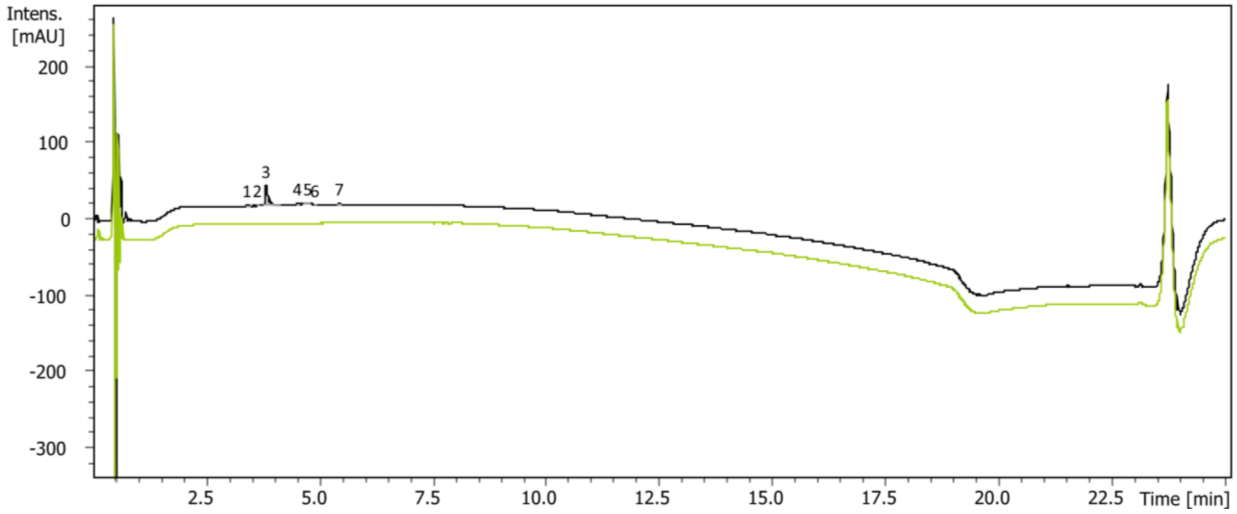

Figure S10: UV trace of the UHPLC-HRMS chromatogram of purified darobactin A F7F in black, as well as the corresponding blank chromatogram at 210 nm in green. The sample was dissolved in the HPLC purification eluent system (H_2O/MeCN mixture + 0.1% FA) and 5 uL were injected to a BEH C18 column using a 95-4.75% A in 18 min gradient (for details see "UPLC-HRMS and HRMS/MS sample preparation and measurement" in the materials and methods section).

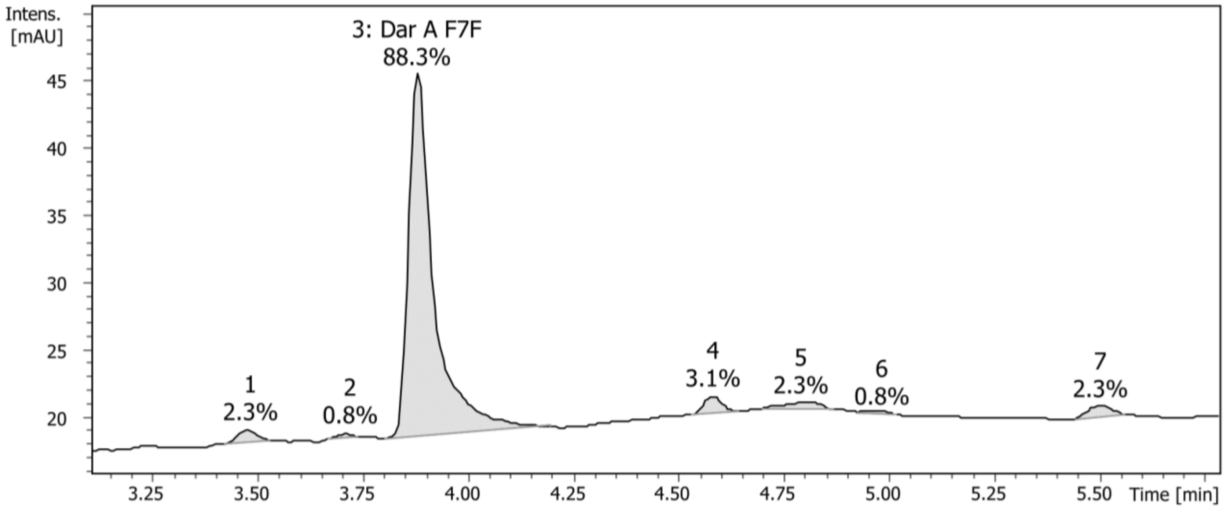

Figure S11: UV trace of the UHPLC-HRMS chromatogram of purified darobactin A F7F (Dar A F7F) at 210 nm zoomed to a retention time of 3-6 min with assigned relative purities. The sample was dissolved in the HPLC purification eluent system (H_2O/MeCN mixture + 0.1% FA) and 5 uL were injected to a BEH C18 column using a 95-4.75% A in 18 min gradient (for details see "UPLC-HRMS and HRMS/MS sample preparation and measurement" in the materials and methods section).

LCMS spectra of darobactin A F7OMe and darobactin A

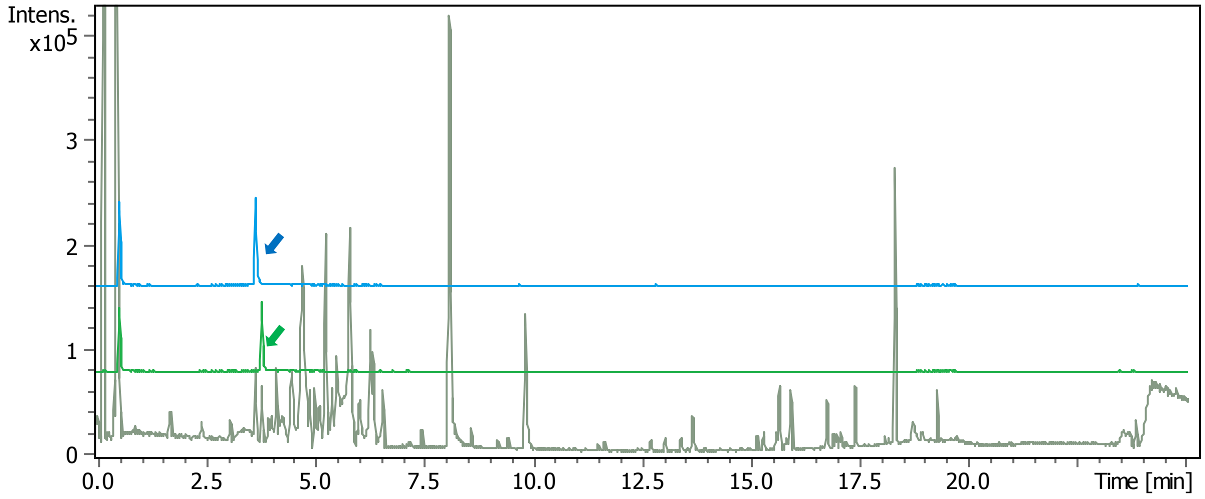

Figure S12: Extracted ion chromatogram (EIC, calcd. $[M + 2H]^{2+}$ ±0.01 Da) of darobactin A F7OMe in green ($C_{48}H_{57}N_{11}O_{13}$; 498.7141 *m/z*), extracted ion chromatogram (EIC, calcd. $[M + 2H]^{2+}$ ±0.01 Da) of darobactin A in blue ($C_{47}H_{55}N_{11}O_{12}$; 483.7089 *m/z*) and the base peak chromatogram (BPC) in grey of the heterologous expression of darobactin A F7OMe by *E. coli* BL21/ pEVOL-pylT-N346A/C348A/ pJK64. Samples were dissolved in 20:80 H_2O/MeCN + 0.1% FA. The blue arrow indicates darobactin A at a retention time of 3.5 min and the green arrow darobactin A F7OMe at 3.7 min.

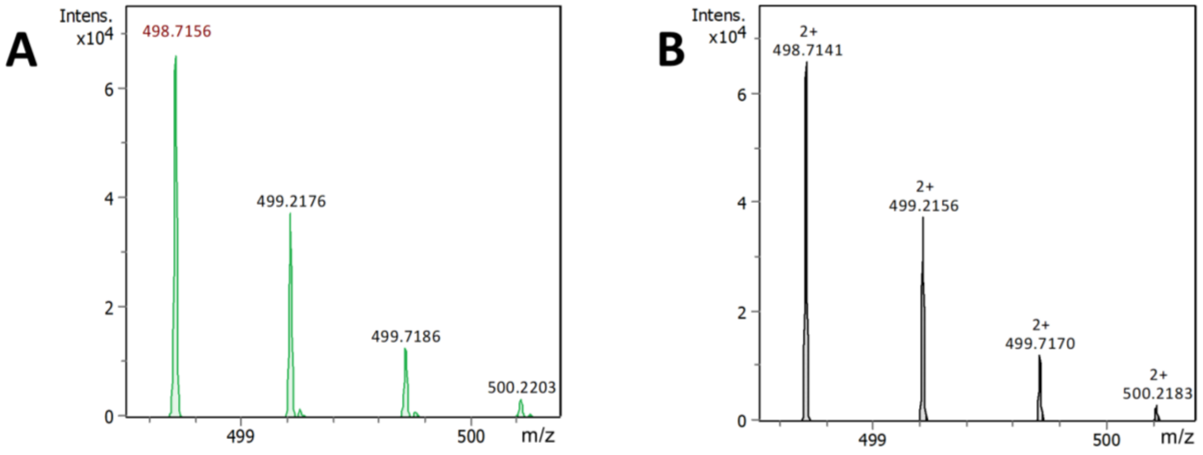

Figure S13: Recorded mass spectrum of **A:** darobactin A F7OMe ($C_{48}H_{57}N_{11}O_{13}$) $[M+2H]^{2+}$ and **B:** simulated mass spectrum of darobactin A F7OMe ($C_{48}H_{57}N_{11}O_{13}$) $[M+2H]^{2+}$ calculated by Compass DataAnalysis.

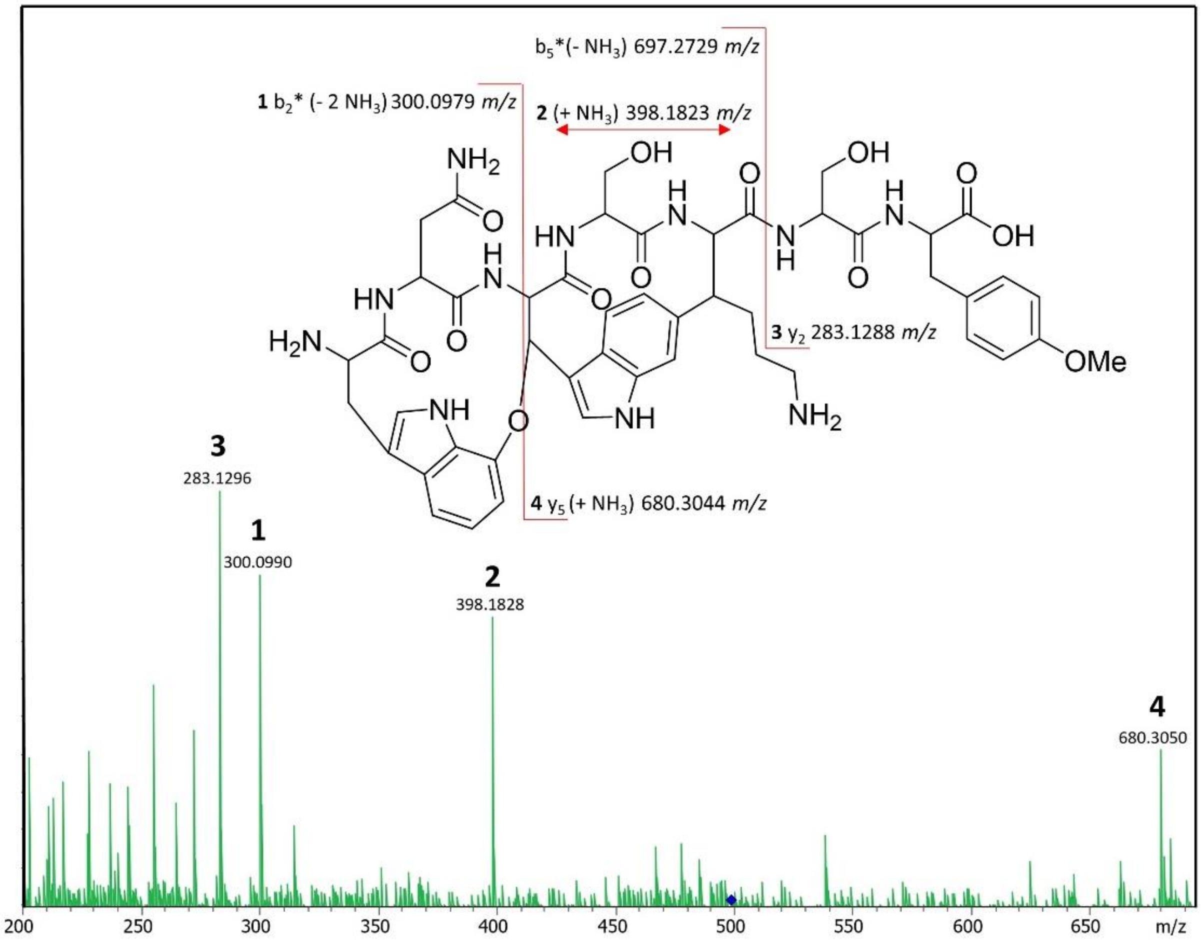

Figure S14: MS/MS spectrum and fragmentation pattern of darobactin A F7OMe. Identified fragments are numbered accordingly.

LCMS spectra of darobactin A F7I and darobactin A

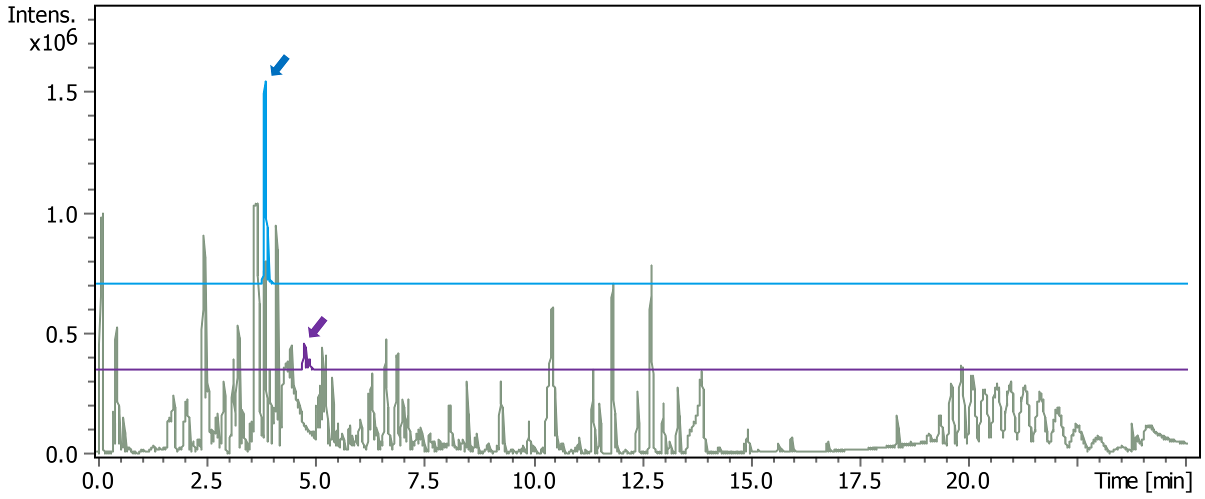

Figure S15: Extracted ion chromatogram (EIC, calcd. $[M + 2H]^{2+}$ ±0.01 Da) of darobactin A F7I in purple ($C_{47}H_{54}N_{11}0_{12}I$; 546.6572 *m/z*), extracted ion chromatogram (EIC, calcd. $[M + 2H]^{2+}$ ±0.01 Da) of darobactin A in blue ($C_{47}H_{55}N_{11}0_{12}$; 483.7089 *m/z*) and the base peak chromatogram (BPC) in grey of the heterologous expression of darobactin A F7I by *E. coli* BL21/ pEVOL-pylT-N346A/C348A/ pJK64. Samples were dissolved in 50:50 H_2O/MeOH. The blue arrow indicates darobactin A at a retention time of 3.9 min and the purple arrow darobactin A F7I with a double peak from 4.8- 4.9 min.

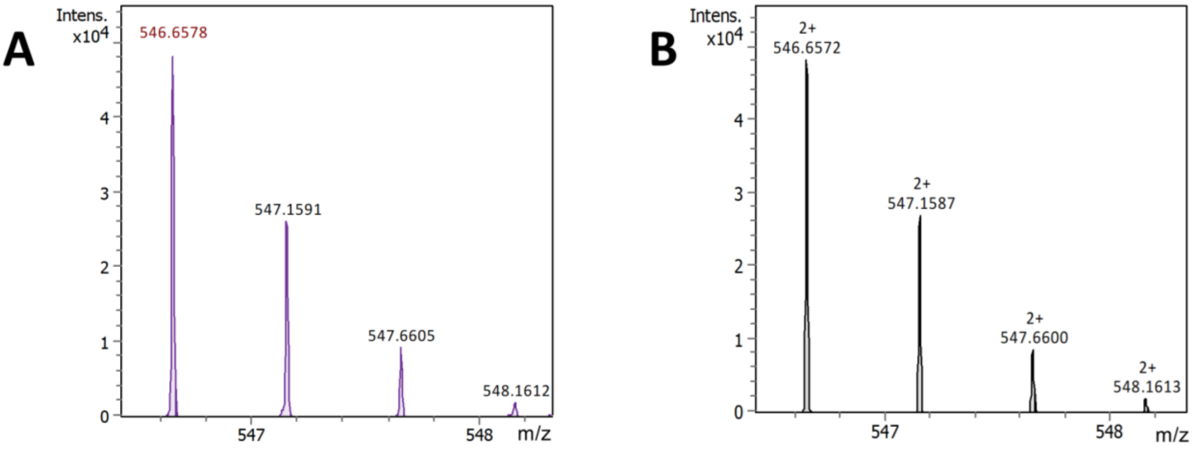

Figure S16: Recorded mass spectrum of **A:** darobactin A F7I ($C_{47}H_{54}N_{11}0_{12}I$) $[M+2H]^{2+}$ and **B:** simulated mass spectrum of darobactin A F7I ($C_{47}H_{54}N_{11}0_{12}I$) $[M+2H]^{2+}$ calculated by Compass DataAnalysis.

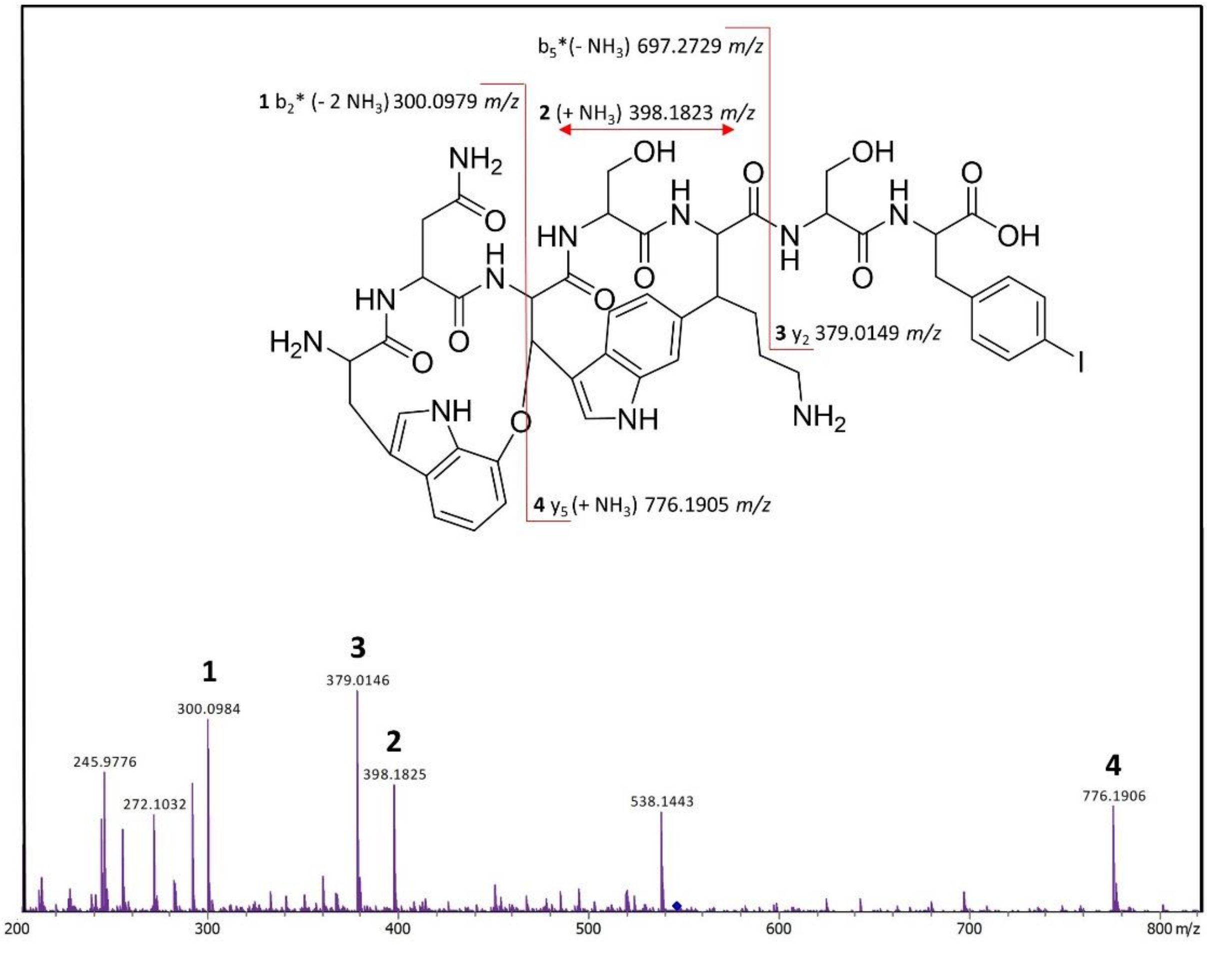

Figure S17: MS/MS spectrum and fragmentation pattern of darobactin A F7I. Identified fragments are numbered accordingly.

LCMS spectra of darobactin A F7F_5 and darobactin A

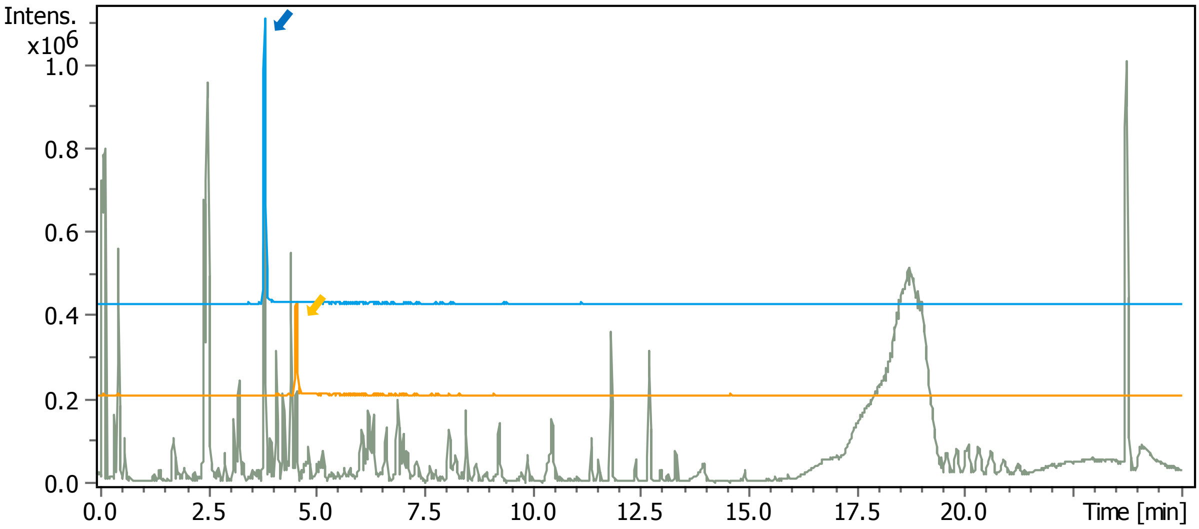

Figure S18: Extracted ion chromatogram (EIC, calcd. $[M + 2H]^{2+}$ ±0.01 Da) of darobactin A F7F_5 in yellow ($C_{47}H_{50}N_{11}O_{12}F_5$; 528.6853 *m/z*), extracted ion chromatogram (EIC, calcd. $[M + 2H]^{2+}$ ±0.01 Da) of darobactin A in blue ($C_{47}H_{55}N_{11}O_{12}$; 483.7089 *m/z*) and the base peak chromatogram (BPC) in grey of the heterologous expression of darobactin A F7F_5 by *E. coli* BL21/ pEVOL-pylT-N346A/C348A/ pJK64. Samples were dissolved in 50:50 H_2O/MeOH. The blue arrow indicates darobactin A at a retention time of 3.9 min and the yellow arrow darobactin A F7F_5 at 4.6 min.

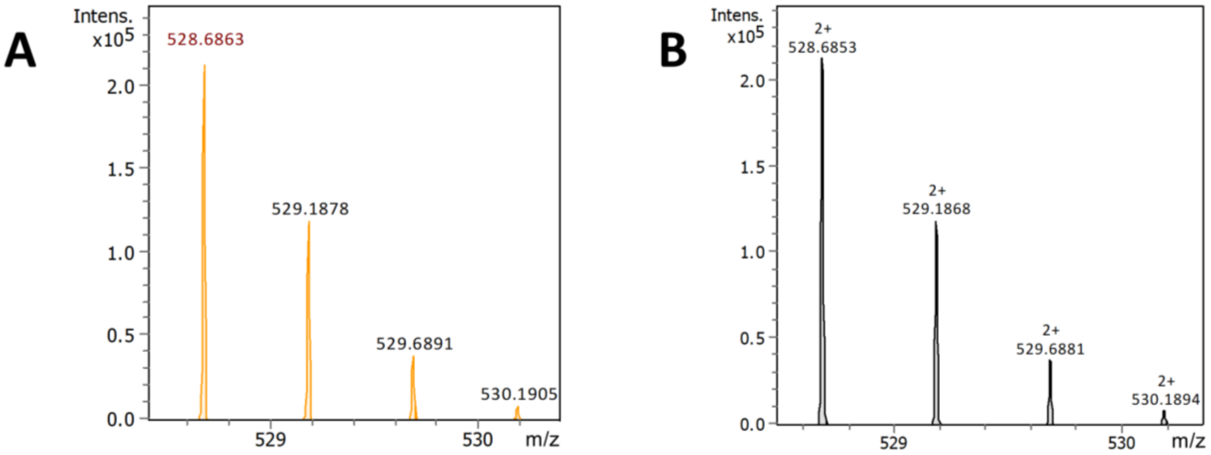

Figure S19: Recorded mass spectrum of **A:** darobactin A F7F_5 ($C_{47}H_{50}N_{11}O_{12}F_5$) $[M+2H]^{2+}$ and **B:** simulated mass spectrum of darobactin A F7F_5 ($C_{47}H_{54}N_{11}O_{12}F_5$) $[M+2H]^{2+}$ calculated by Compass DataAnalysis.

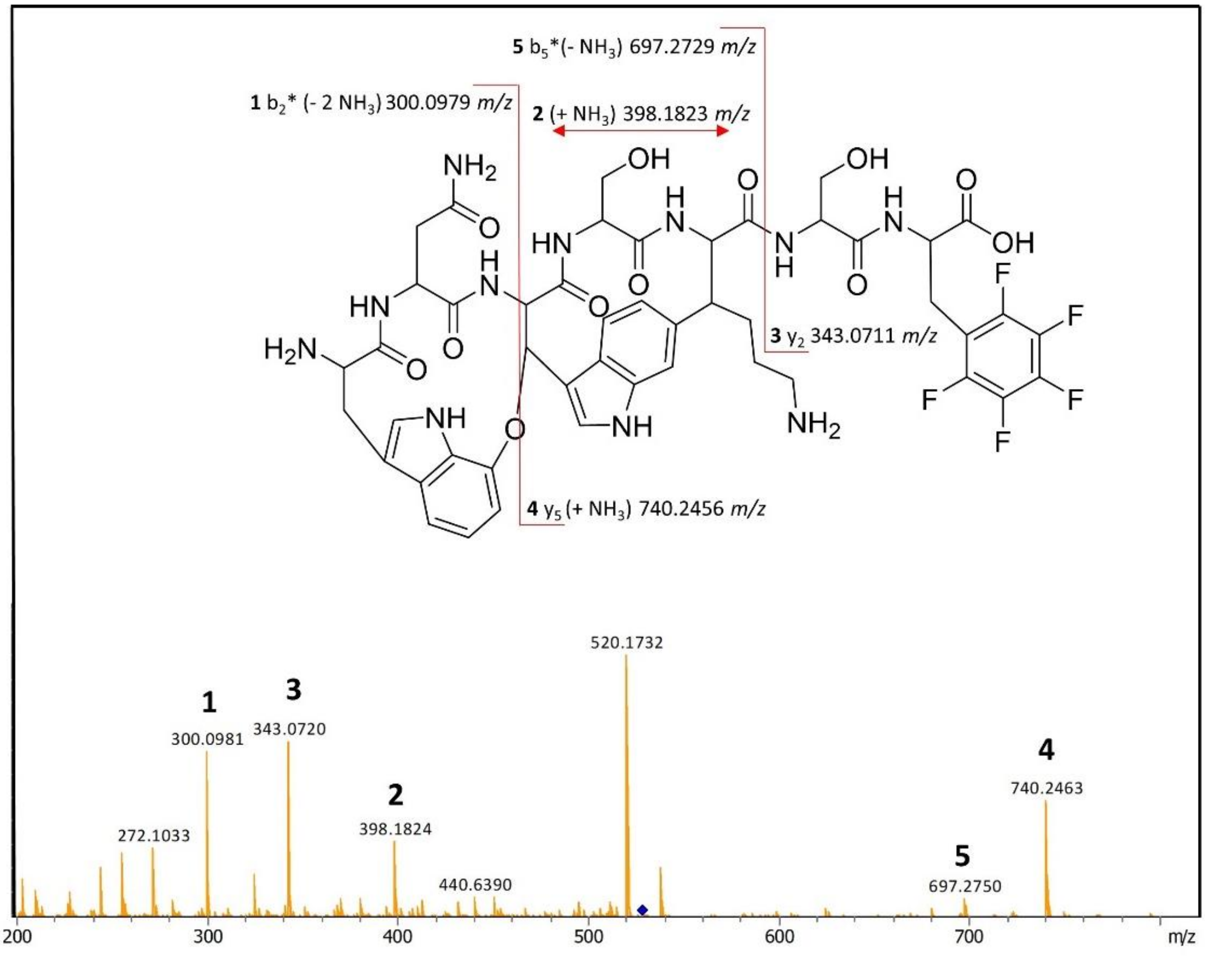

Figure S20: MS/MS spectrum and fragmentation pattern of darobactin A F7F$_5$. Identified fragments are numbered accordingly.

In vitro activity

Table S3: Results of the activity assessment of darobactin A F7F (DAR F7F) in comparison to darobactin B (DAR B) and darobactin A (DAR A) as well as standard antibiotics ceftazidime (CTZ), ciprofloxacin (CIP) and gentamicin (GEN) against Escherichia coli (Ec), Pseudomonas aeruginosa (Pa), Acinetobacter baumannii (Ab), Klebsiella pneumoniae (Kp) and Staphylococcus aureus (Sa). Minimum inhibitory concentration (MIC) is given µg/mL.

			MIC µg/mL					
			**DAR B**	**DAR A**	**DAR F7F**	CTZ	CIP	GEN
Ec	**MG1655**	**bamA6ΔbamB**	n.d.	< 0.03	**0.125**	0.125	0.008	64
Ec	**ATCC25922**		1-0.5	n.d.	**8-4**	0.25	0.008	0.5
Ec	**ATCC25922**	**ΔTolC**	0.5-0.25	n.d.	**8-4**	0.25	0.002	0.25
Ec	**NRZ14408**	**mcr-1**	1- 0.5 [2]	4 [2]	**8**	32-16	>0.5	>64
Pa	**PAO1**		2-1[1]	4 - 2 [2]	**8**	2	0.25	2
Pa	**PAO750**	**efflux deficient**	0.5	n.d.	**8**	1	0.004	0.125
Pa	**PA103**		8 [3]	n.d.	**64**	8	0.06	2
Pa	**ATCC27853**		8 [1]	> 64	**> 64**	4	0.125	2
Pa	**EXT111762**		2-1 [1]	> 64	**64**	16	0.5	0.5
Ab	**ATCC19606**		32 [2]	> 64	**> 64**	16	>0.5	32-16
Kp	**DSM30104**		2-1 [2]	4	**8**	0.06	0.06 - 0.03	0,125 - 0.06
Kp	**ATCC700603**		2-1 [3]	n.d.	**16 - 8**	> 64	> 0.5	64
Sa	**ATCC33592**		>64	>64	**>64**	>64	0.5	0.5

[1] Microbiology Spectrum 2023, 11 (1), e04437-22.
[2] Microbiology Spectrum 2021, 9 (3), e01535-21.
[3] bioRxiv 2024.05.30.596569.

NMR data of darobactin A F7F

Table S4: ^{1}H (700 MHz), ^{13}C (176.1 MHz, DEPTQ-135), and ^{19}F (658.9 MHz) NMR data of darobactin A F7F (D_2O; δ in ppm) alongside correlation data from HMBC, COSY, TOCSY, and NOESY experiments. For ^{13}C measurements 3-(trimethylsilyl)propionic-2,2,3,3-d_4 acid sodium salt (TSPA) was used as external standard. For ^{19}F measurements α,α,α-trifluorotoluene served as an external standard. For ^{1}H, ^{13}C DEPTQ-135, ^{19}F, COSY, HSQC, and HMBC experiments a concentrated sample of darobactin A F7F was used, while HSQC-TOCSY, ^{1}H,^{1}H-TOCSY, NOESY, and ROESY experiments were acquired for a dilute sample. The following abbreviations are used in this table: mult.: multiplicity, int.: integral, obs.: obscured, nd: not detected. For atom numbering, cf. Figure 4a.

Amino acid	Position	δ_C [ppm], C-type, mult. ($J_{C,F}$ in Hz)	δ_H [ppm][a], mult. ($J_{H,H}$ in Hz), int.	δ_F [ppm], mult. ($J_{F,H}$ in Hz)	HMBC[b] correlation to position	COSY[b], [c] correlation to position	HSQC-TOCSY[b], [d] correlation to position	^{1}H,^{1}H-TOCSY[b], [c] correlation to position	NOESY[b] correlation to position	ROESY[b] correlation to position
Trp (N-terminal)	1	54.7[e]	4.09, dd (11.5, 8.4)[f]				(2)	(2)	(2), (4)	(4)
	2	26.4[e]	3.61,[e] obs.<br>3.37,[e] obs.					(1)	(1), 4, 9, (8)	(4), (9)
	3	108.6,[g] C_q								
	4	124.6, CH, s	7.41, s, 1H		3, 5/10				(1), 2	(1), (2)
	5	129.04 / 128.99, C_q, s								
	6	145.1, C_q, s								
	7	108.8, CH, s	7.31, m, obs., 1H[h]		5, 9		9		17	17
	8	120.1, CH, s	7.25, t (7.7), 1H		6, 10				(2), 17	(17)
	9	113.8, CH, s	7.30, m, obs., 1H[h]		(7), 5/10		7		2	(2)
	10	129.04 / 128.99, C_q, s								
	11	nd								
Asn	12	50.7, CH, s	3.39, t (6.8), 1H		15	13	13	13	13	13
	13	39.0, CH_2, s	2.22, dd (18.1, 7.0), 2H		(12), 14, 15	12	12	12	12	12
	14	173.8, C_q, s								
	15	168.5, C_q, s								
Trp	16	63.3, CH, s	4.74, obs., 1H[i]		(15), 26	17	17	17		
	17	76.8, CH, s	6.25, d (8.9), 1H		(6), 18, 19	16	16	16	7, (8), 24	7, (8), 24
	18	111.7, C_q, s								
	19	124.4, CH, s	7.92, s, 1H		18, 20, 25					
	20	137.1, C_q, s								
	21	110.6, CH, s	7.54, s, 1H		25, 31			(23)	30, 31, 32, 33	30, 32
	22	133.1,[g] C_q								
	23	125.0, CH, s	7.03, d (8.3), 1H		(21), 25	24	24	(21), 24	24, (27), 31, (33)	24, (27), 31
	24	117.3, CH, s	7.51, d (8.3), 1 H		20, 22	23	23	23	17, 23, (27), (31)	17, 23, (27)
	25	124.9, C_q, s								
	26	167.9, C_q, s								
Ser	27	54.0, CH, s	4.00, t (6.3), 1H[j]		26, (28), 29	28	28	28	(23), (24)	(23), (24), 28
	28	61.9, CH_2, s	3.28, dd (11.8, 6.7), 1H<br>3.19, dd (11.6, 6.6), 1H		29	27	27	27		27

Amino acid	Position	δ_C [ppm], C-type, mult. ($J_{C,F}$ in Hz)	δ_H [ppm][a], mult. ($J_{H,H}$ in Hz), int.	δ_F [ppm], mult. ($J_{F,H}$ in Hz)	HMBC[b] correlation to position	COSY[b], [c] correlation to position	HSQC-TOCSY[b], [d] correlation to position	^{1}H,^{1}H-TOCSY[b], [c] correlation to position	NOESY[b] correlation to position	ROESY[b] correlation to position
	29	168.1, C_q, s								
Lys	30	60.2, CH, s	4.31, d (10.6), 1H		29, 31, 35	31	31, 32	(31), 32, 33, 34	21, 31, 32, 33	21, 31, (32)
	31	48.2, CH, s	3.12, obs., 1H			30, 32	30	(30), 32, 33	21, 23, (24), 30, (32), 33	23, 30, 33
	32	25.8, CH_2, s	2.13, m, obs., 1H[k]<br>1.80, m, obs., 1H[l]			31, 33	(30), (34)	30, 31, 33, 34	21, 30, (31), 33	21, (30), 33
	33	25.5, CH_2, s	1.97, m, obs., 1H[m]<br>1.81, m, obs., 1H[l]			32, 34	(30), 32, 34	30, 31, 32, 34	21, (23), 30, 31, 32, 34	31, 32, 34
	34	39.4, CH_2, s	3.07, t (7.0), 2H		33	33	30, 32, 33	30, 32, 33	33	33
	35	171.8, C_q, s								
Ser	36	55.8, CH, s	4.50, t (5.5), 1H		(35), (37), 38	37	37	37	37	
	37	61.1, CH_2, s	3.86, d (5.6), 2H[n]		38	36	36	36	36	
	38	170.3, C_q, s								
F-Phe (C-terminal)	39	56.42, CH, s	4.52, dd (6.6, 5.6), 1H		(38), (40), 41, 45	40	40	40		
	40	36.8, CH_2, s	3.23, dd (14.0, 5.0), 1H<br>3.09, obs., 1H		39, 41, 42/42’, 45	39	39	39		42, 42’
	41	133.3,[g] C_q								
	42, 42’	131.1, CH, d (8.2)	7.34, dd (8.2, 5.7), 2H		40, 42/42’, 44	43, 43’	(42/42’), 43, 43’	43, 43’		40
	43, 43’	115.1, CH, d (21.3)	7.19, t (8.7), 2H		41, 44	42, 42’	42, 42’, (43/43’)	42, 42’		
	44	161.7, C_q, d (241.5)								
	44-F			-117.94, m[o]						
	45	177.2, C_q, s								

[a] Unless stated otherwise, the ^{1}H shifts of multiplets and obscured signals were extracted from the HSQC spectrum (700 MHz, 176.1 MHz). [b] Brackets indicate weak correlation signals. [c] Geminal coupling ($^2J_{H,H}$) is not displayed. [d] $^1J_{C,H}$ coupling is not displayed. [e] The chemical shift of was extracted from the HSQC-TOCSY spectrum (700 MHz, 176.1 MHz) as H-2 was obscured by the glycerol peak (contaminant)/ the signal of H-12, and C-2 could not be observed in the DEPTQ-135 experiment. Therefore, the signals of corresponding to H-2 / C-2 could only be observed for the dilute sample. [f] The ^{1}H NMR signal of H-1 could only be observed for the dilute sample. [g] The ^{13}C shift was extracted from the HMBC spectrum (700 MHz, 176.1 MHz). [h] The multiplicity of H-7 and H-9 is denoted as m because the true multiplicity is not resolved due to overlap of both signals. The signal for H-7 and H-9 is observed as a pseudo-triplet having an integral of 2H. Therefore, for each of these positions an integral of 1H was assigned. [i] The observed integral for this signal was 2H due to overlap of the proton signal for H-16 with the H_2O signal. The expected integral for H-16 is 1H, and a CH group can be inferred from the signal phase of the DEPTQ-135 and HSQC experiments. [j] The observed integral for this signal was 2H due to overlap of the proton signal for H-27 with an impurity. The expected integral for H-27 is 1H, and a CH group can be inferred from the signal phase of the DEPTQ-135 and HSQC experiments. [k] The observed integral for this signal was 3H due to overlap of the proton signal for H-32a with an impurity. The expected integral for H-32a is 1H. [l] The observed integral for this signal was 3H due to overlap of the proton signals for H-32b, and H-33b with an impurity. For each of these positions an integral of 1H was assigned. [m] The observed integral for this signal was 2H due to overlap of the proton signal for H-33a with the acetic acid signal (contaminant). The expected integral for H-33a is 1H. [n] The observed integral for this signal was 1H, but a CH_2 group can be inferred from the signal phase of the DEPTQ-135 and HSQC experiments. This might indicate that even though a doublet is observed, the true multiplicity of the signal is more complex and obscured by the neighboring glycerol signal (contaminant). Thus, an integral of 2H was assigned for H-37. [o] The observed multiplicity matches with literature data[3] for 4-fluorophenylalanine, even though the resolution is not sufficient to determine coupling constants.

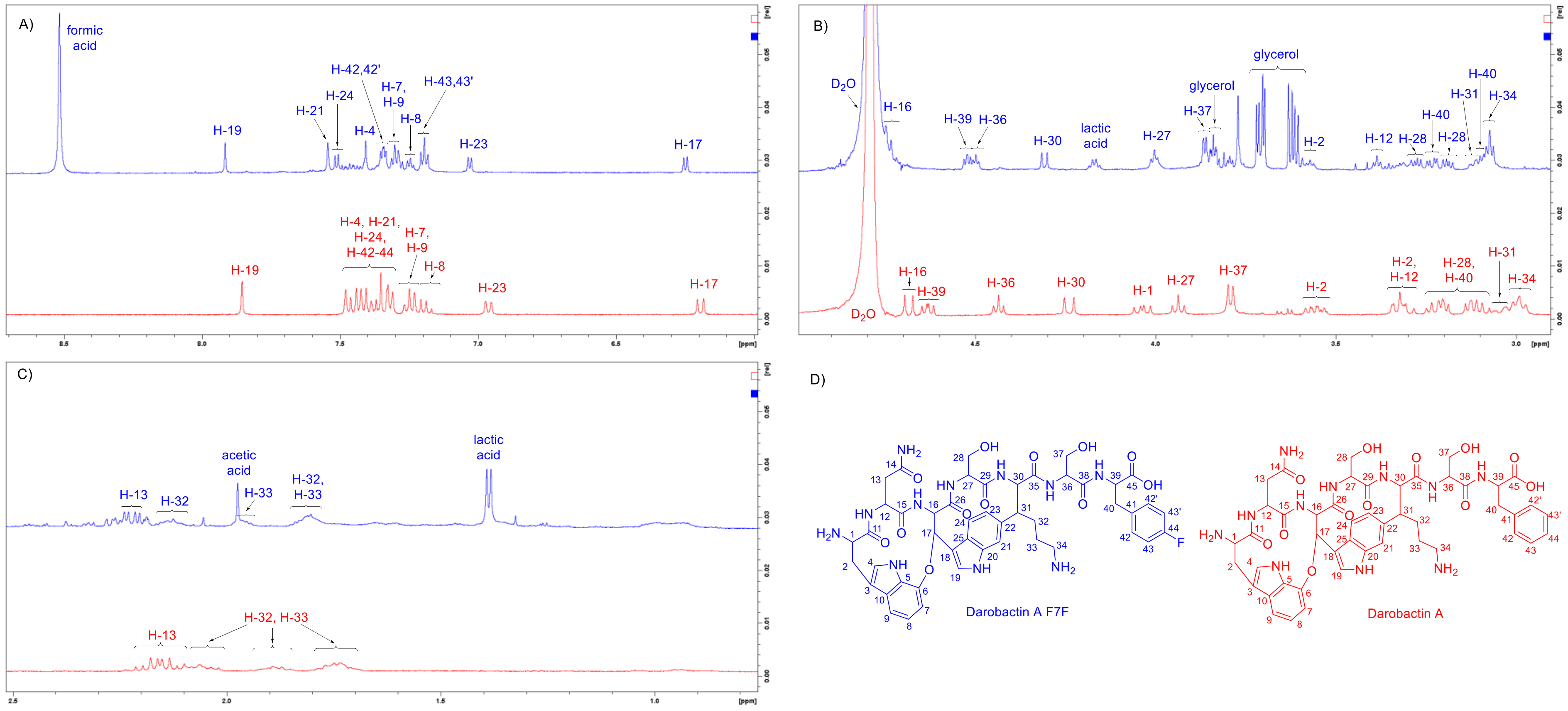

Figure S21: Comparison of the ^{1}H-NMR spectra of darobactin A F7F (blue) and darobactin A (red). Both spectra were measured in D_2O. Spectra of darobactin A F7F were recorded at 700 MHz, while spectra of darobactin A were recorded at 400 MHz.

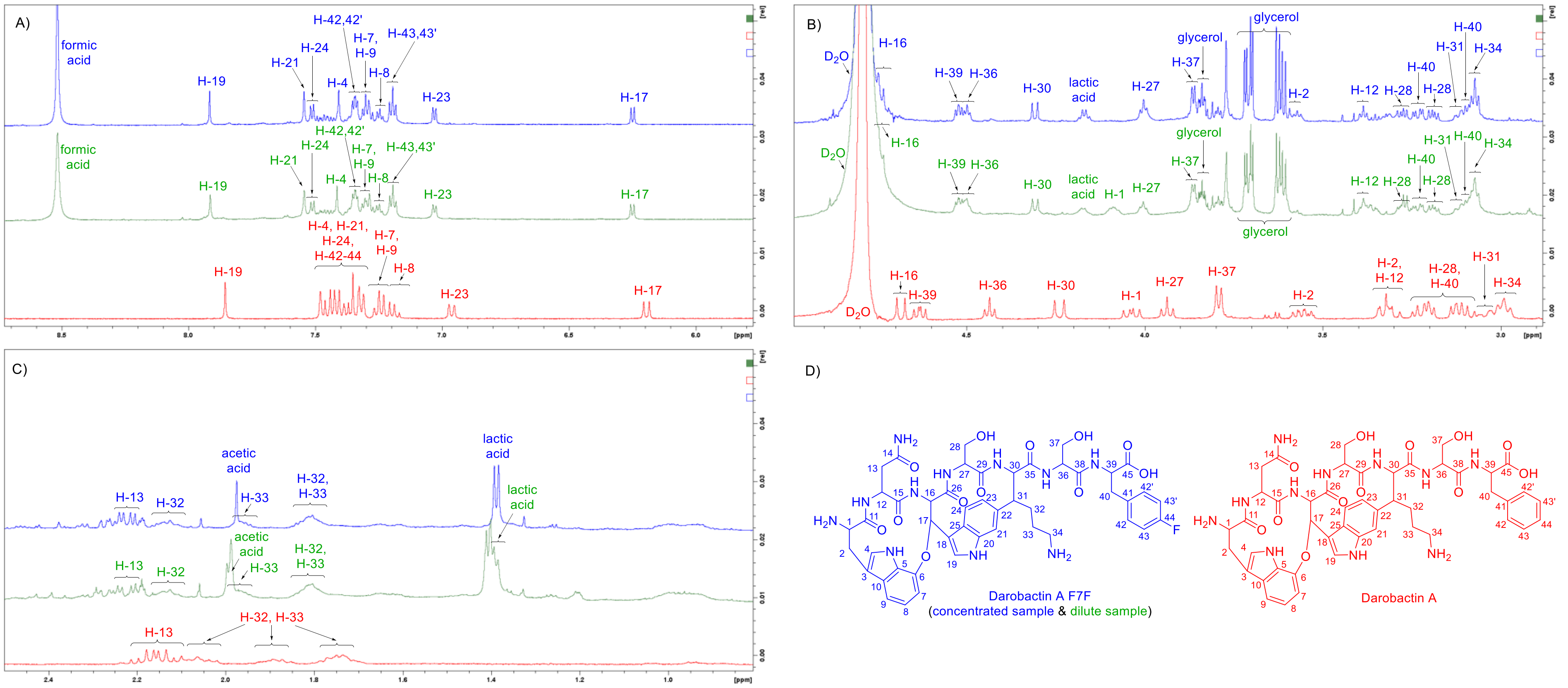

Figure S22: Comparison of the ^{1}H-NMR spectra of darobactin A F7F (blue: concentrated sample; green: dilute sample) and darobactin A (red). All spectra were measured in D_2O. Spectra of darobactin A F7F were recorded at 700 MHz, while spectra of darobactin A were recorded at 400 MHz.

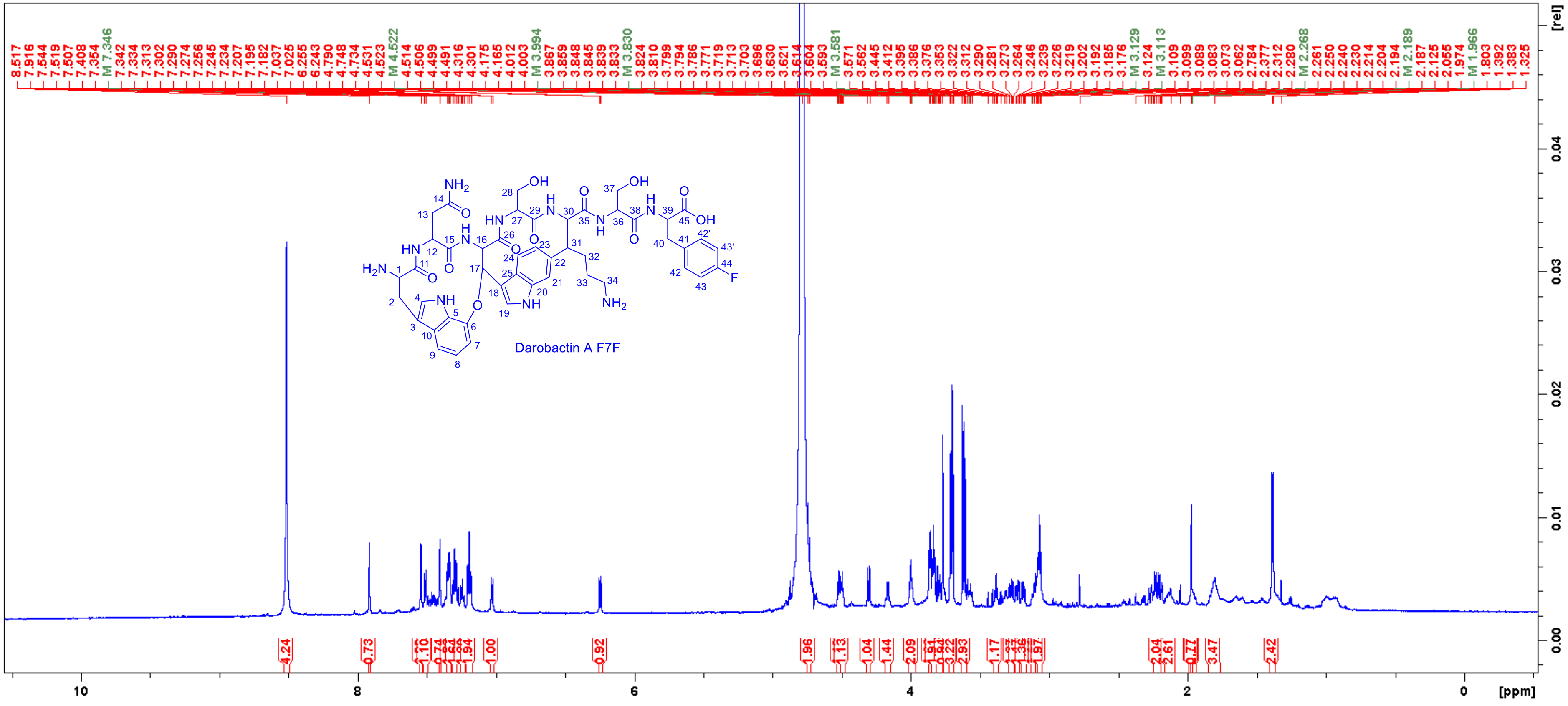

Figure S23: ^{1}H-NMR spectrum of darobactin A F7F (concentrated sample, D_2O, 700 MHz).

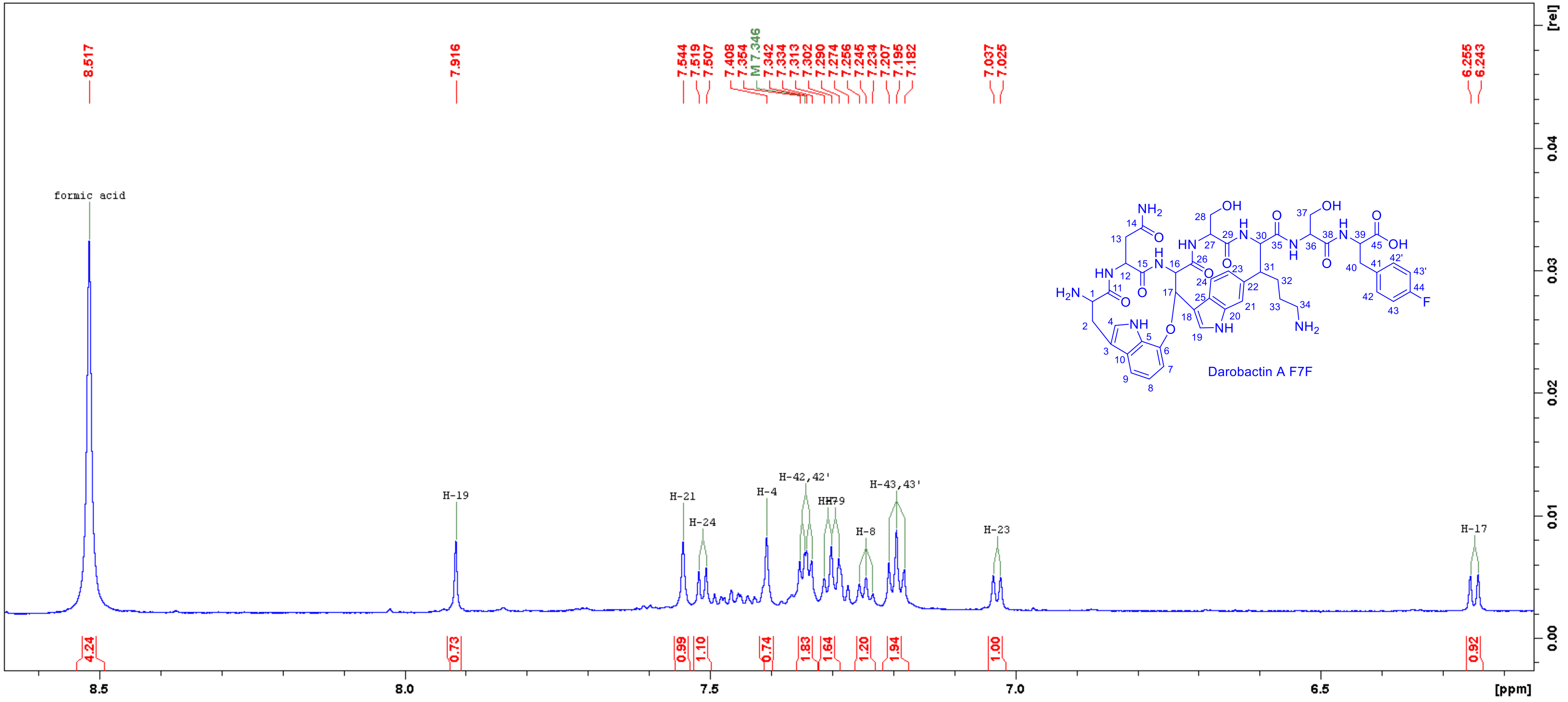

Figure S24: ^{1}H-NMR spectrum of darobactin A F7F (concentrated sample, D_2O, 700 MHz). Close-up in the range of 8.6– 6.2 ppm.

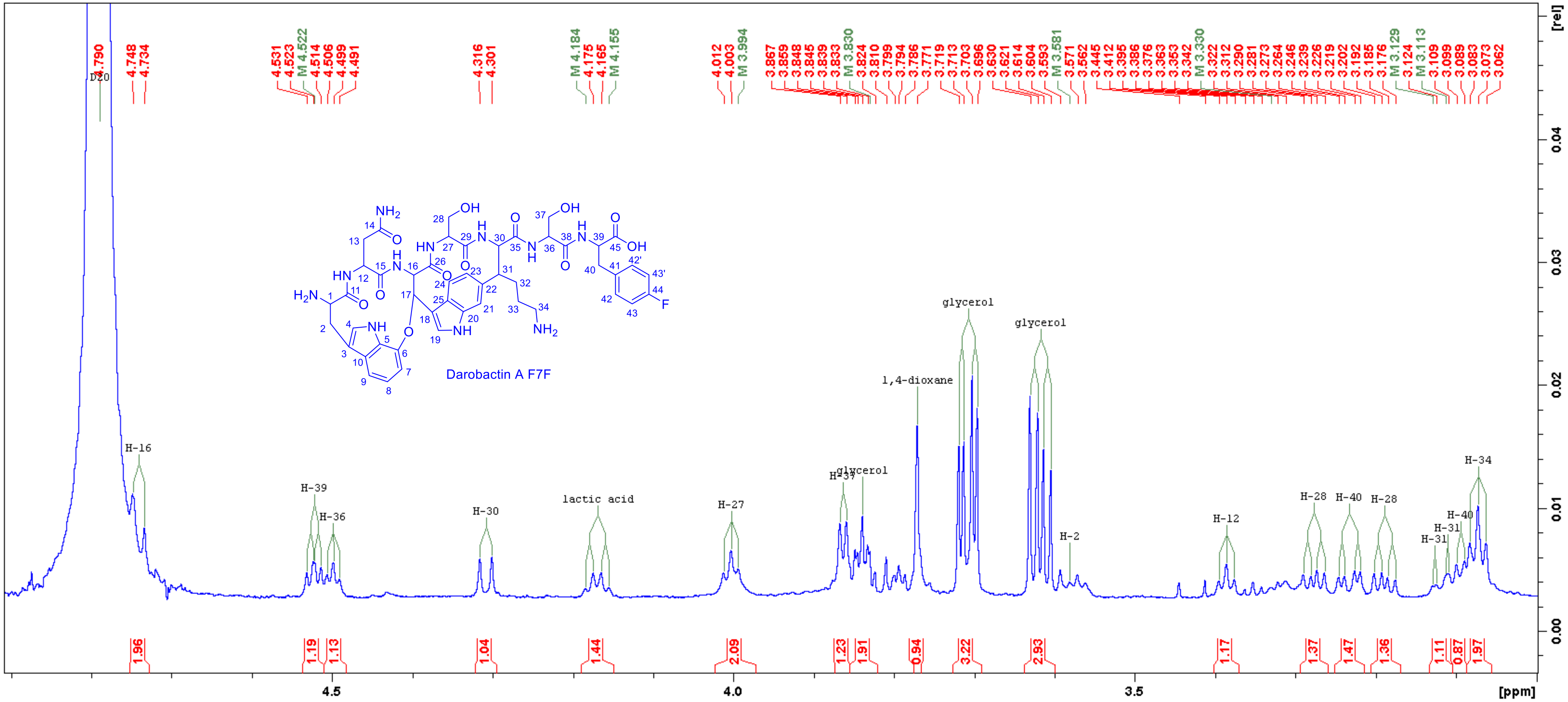

Figure S25: ^{1}H-NMR spectrum of darobactin A F7F (concentrated sample, D_2O, 700 MHz). Close-up in the range of 4.9– 3.0 ppm.

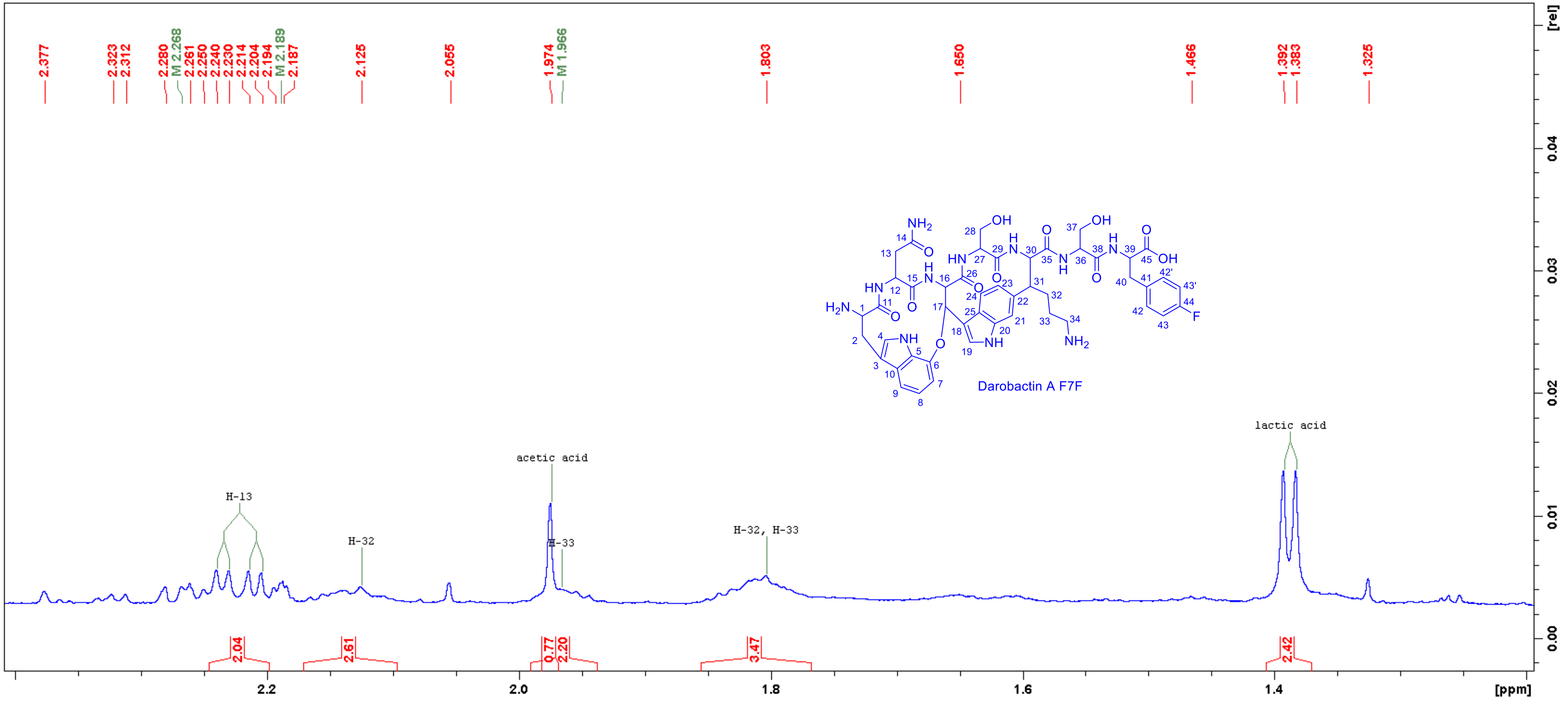

Figure S26: ^{1}H-NMR spectrum of darobactin A F7F (concentrated sample, D_2O, 700 MHz). Close-up in the range of 2.4 – 1.2 ppm.

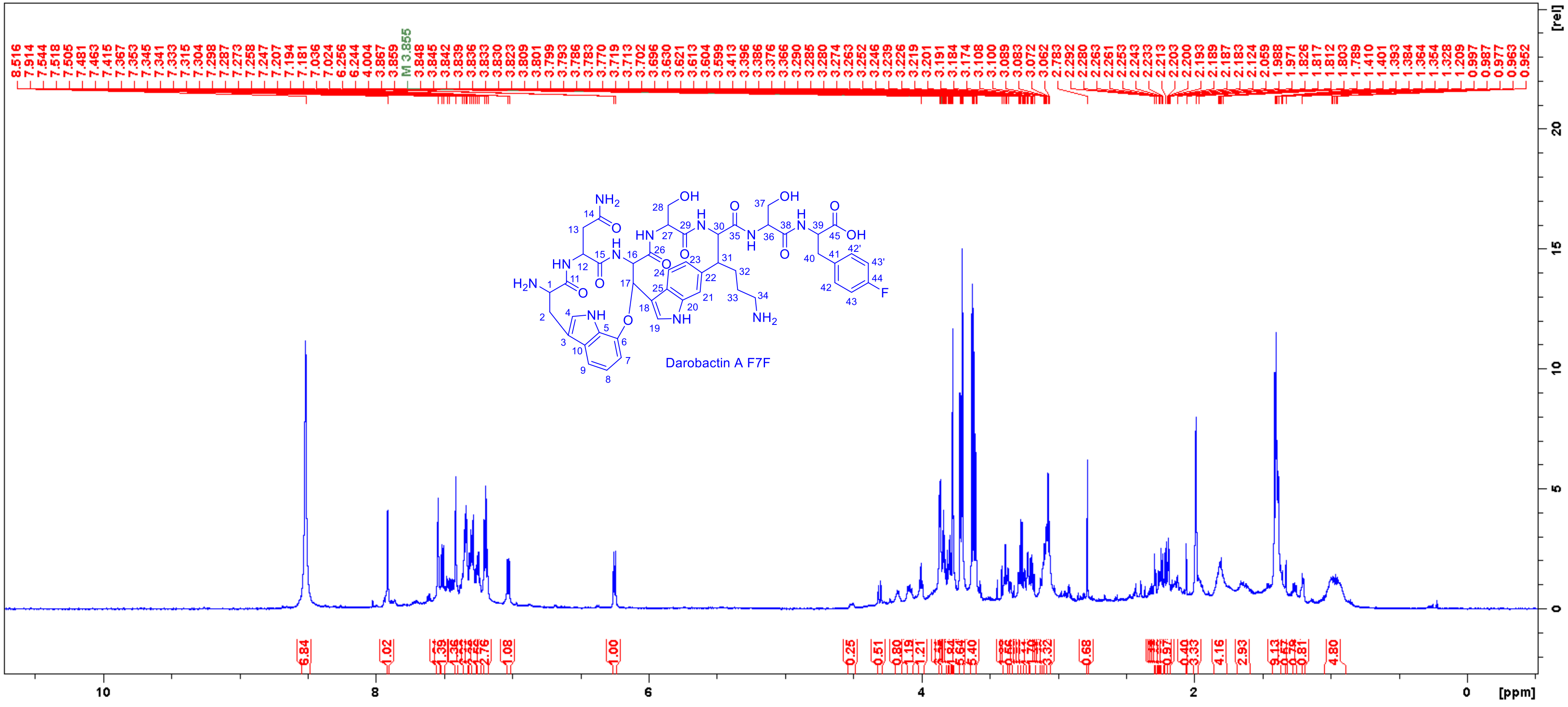

Figure S27: ^{1}H-NMR spectrum of darobactin A F7F (dilute sample, D_2O, 700 MHz, measurement with H_2O suppression.

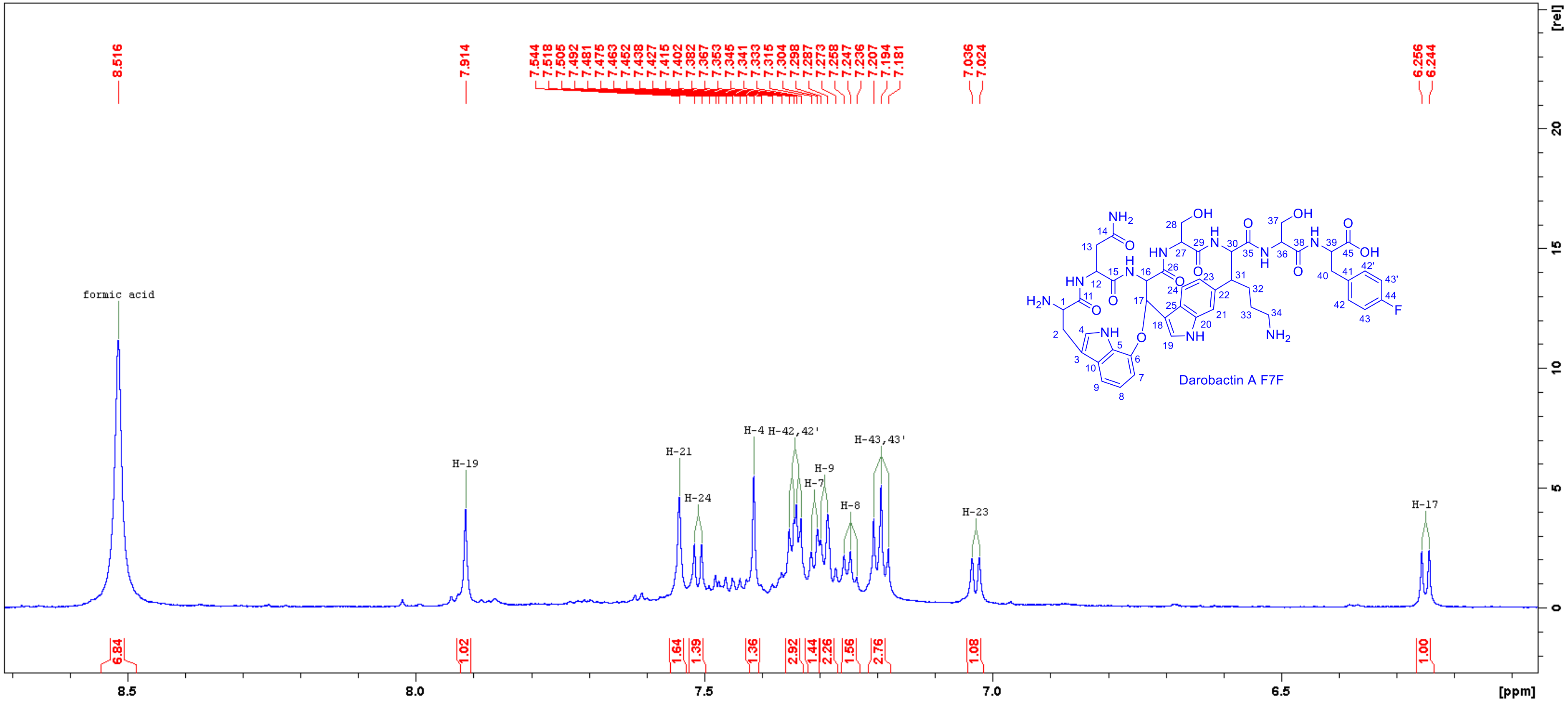

Figure S28: ^{1}H-NMR spectrum of darobactin A F7F (dilute sample, D_2O, 700 MHz, measurement with H_2O suppression). Close-up in the range of 8.7– 6.1 ppm.

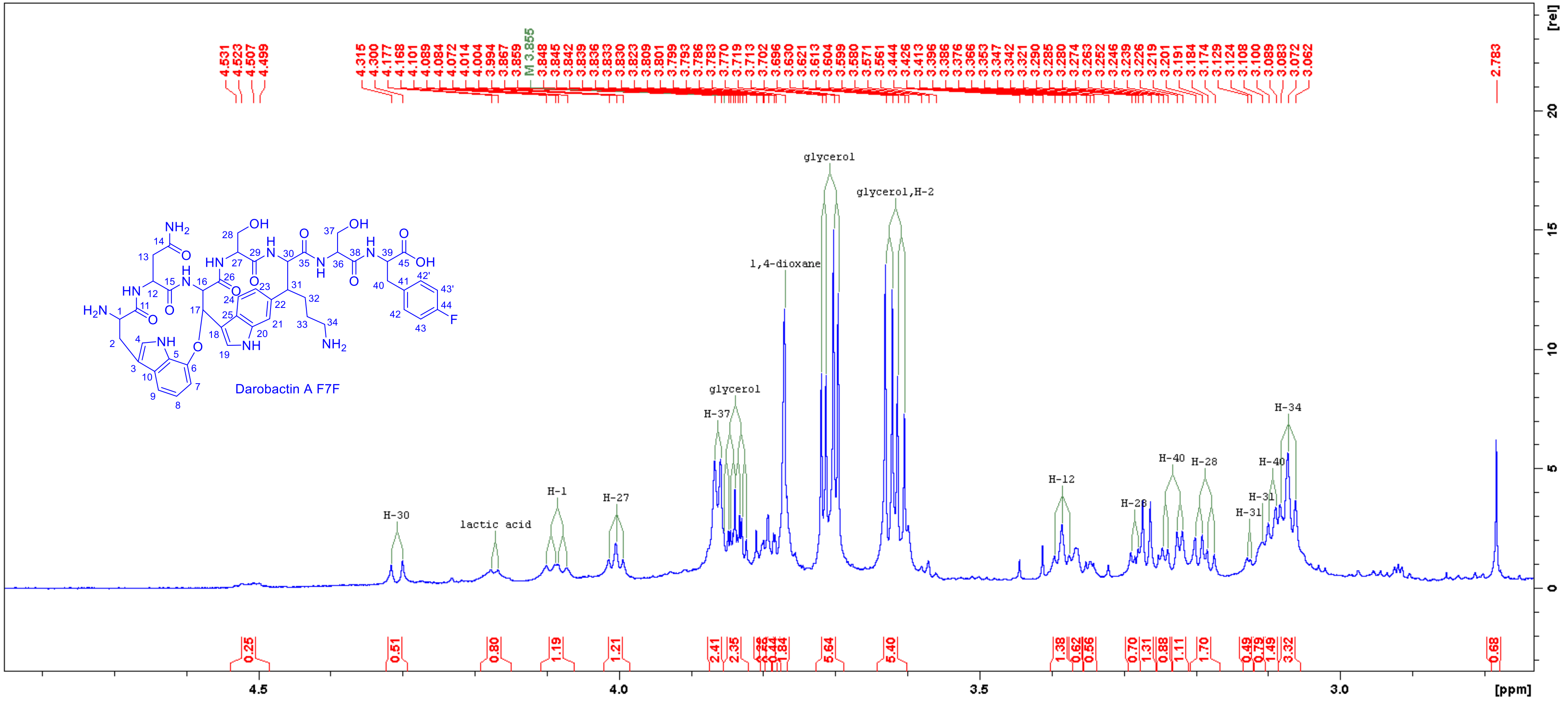

Figure S29: ^{1}H-NMR spectrum of darobactin A F7F (dilute sample, D_2O, 700 MHz, measurement with H_2O suppression). Close-up in the range of 4.8– 2.8 ppm.

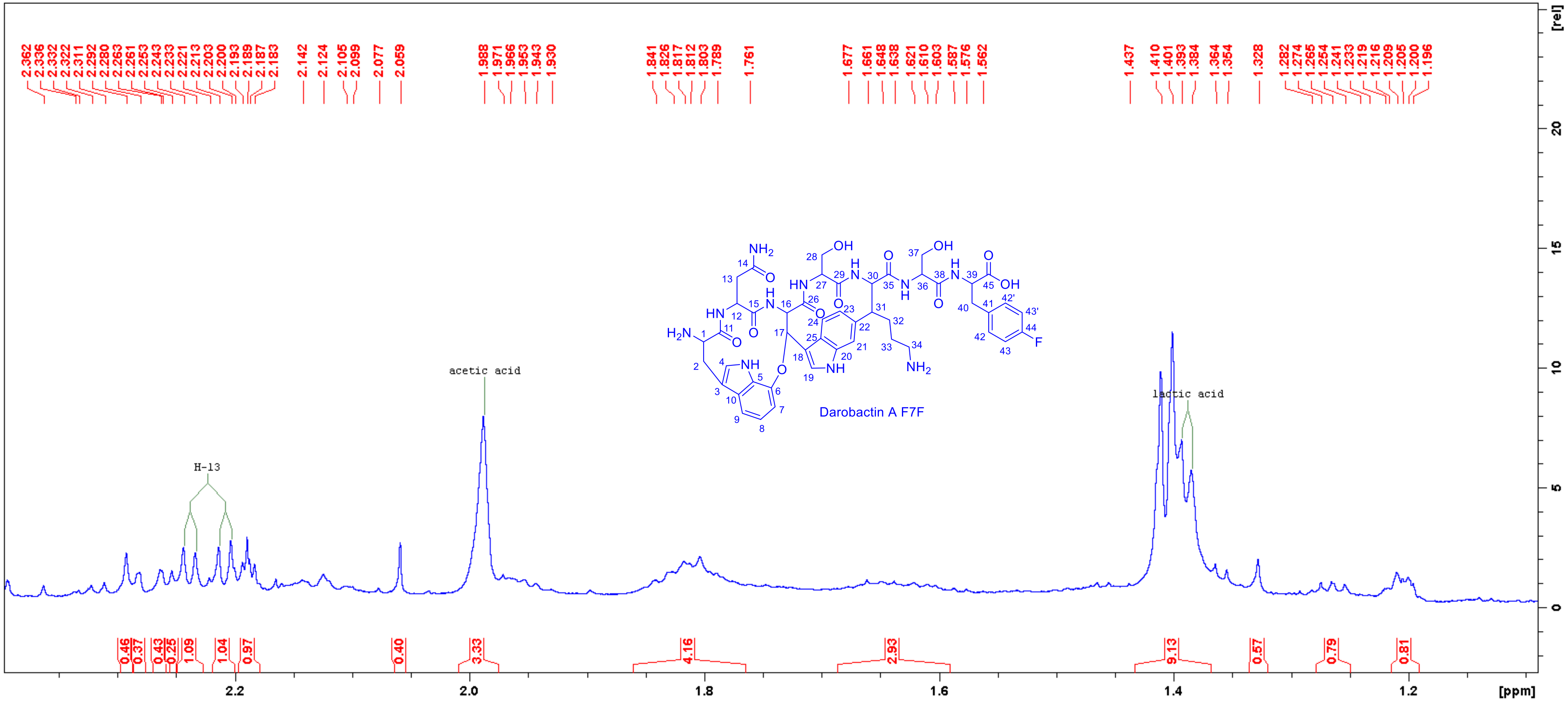

Figure S30: ^{1}H-NMR spectrum of darobactin A F7F (dilute sample, D_2O, 700 MHz, measurement with H_2O suppression). Close-up in the range of 2.25– 1.10 ppm.

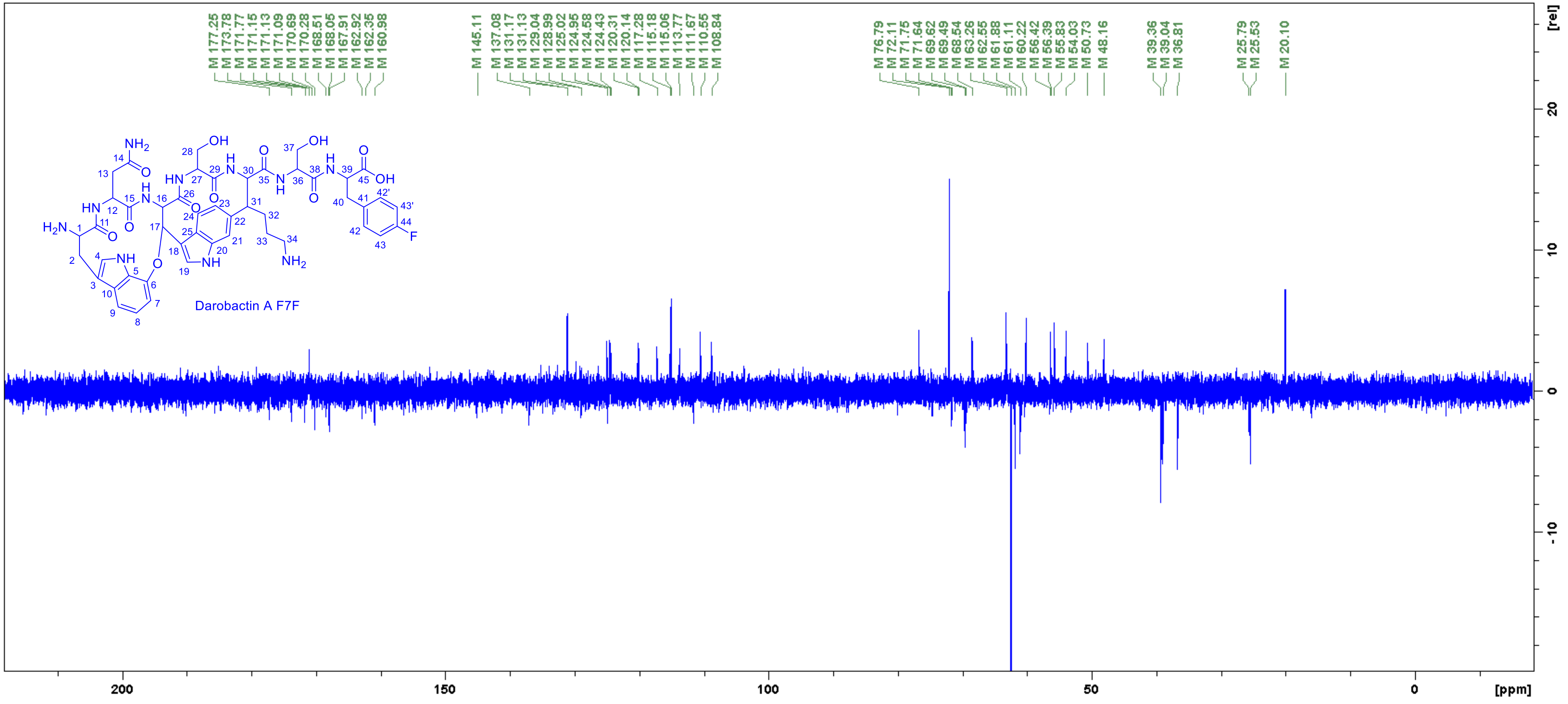

Figure S31: ^{13}C-NMR spectrum of darobactin A F7F (DEPTQ-135, D_2O, 176.1 MHz). For the measurement 3-(trimethylsilyl)propionic-2,2,3,3-d_4 acid sodium salt (TSPA) was used as external standard.

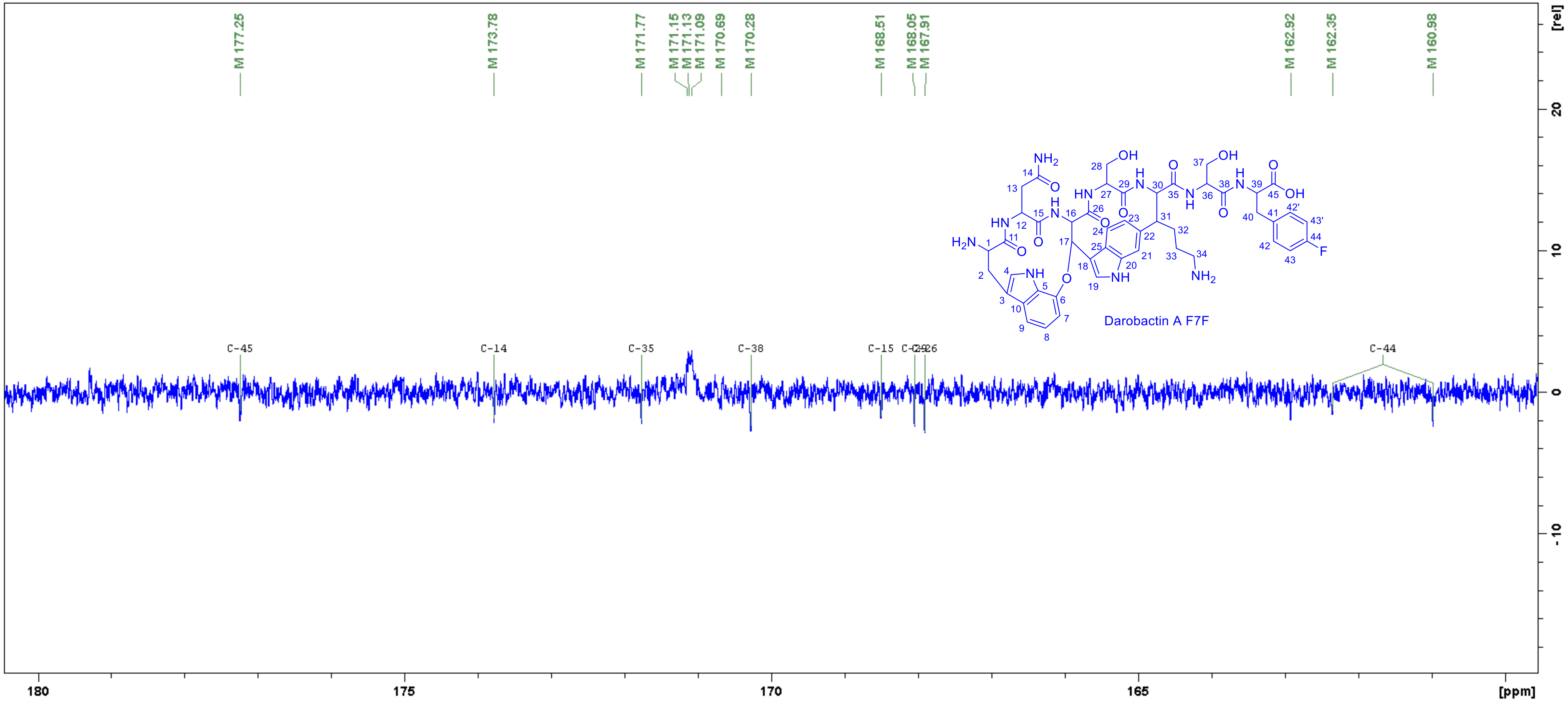

Figure S32: ^{13}C-NMR spectrum of darobactin A F7F (DEPTQ-135, D_2O, 176.1 MHz). For the measurement 3-(trimethylsilyl)propionic-2,2,3,3-d$_4$ acid sodium salt (TSPA) was used as external standard. Close-up in the range of 180.0– 160.0 ppm.

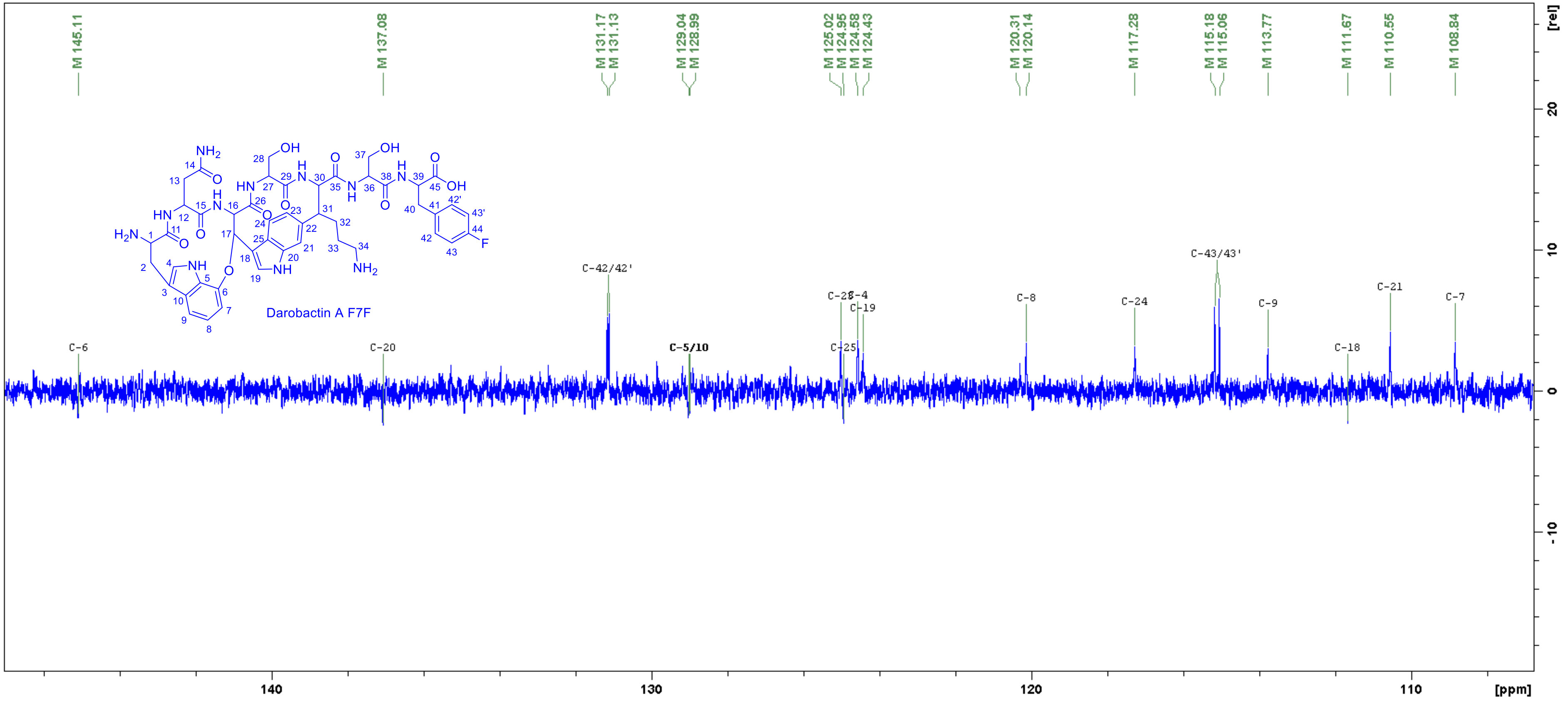

Figure S33: ^{13}C-NMR spectrum of darobactin A F7F (DEPTQ-135, D_2O, 176.1 MHz). For the measurement 3-(trimethylsilyl)propionic-2,2,3,3-d_4 acid sodium salt (TSPA) was used as external standard. Close-up in the range of 146.0– 108.0 ppm.

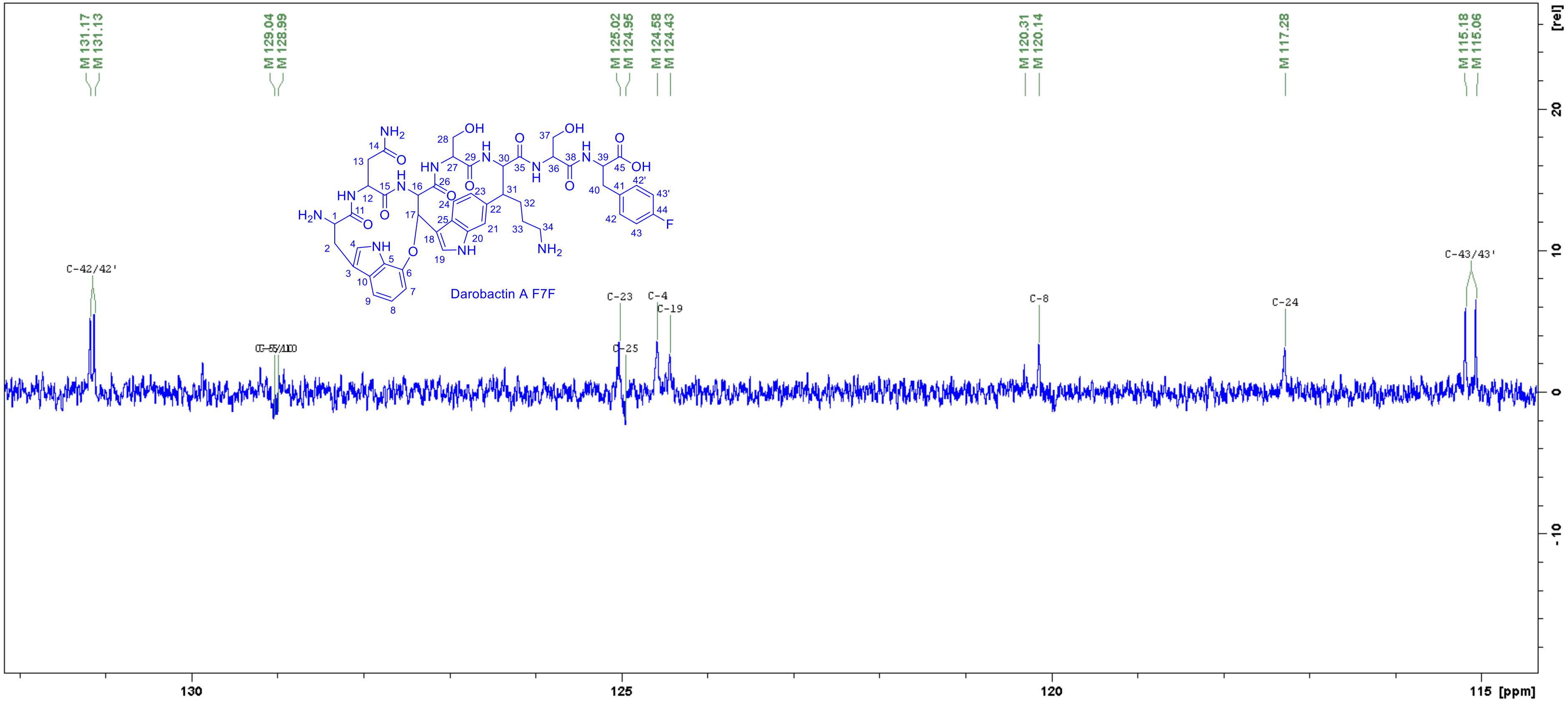

Figure S34: ^{13}C-NMR spectrum of darobactin A F7F (DEPTQ-135, D_2O, 176.1 MHz). For the measurement 3-(trimethylsilyl)propionic-2,2,3,3-d_4 acid sodium salt (TSPA) was used as external standard. Close-up in the range of 132.0– 115.0 ppm.

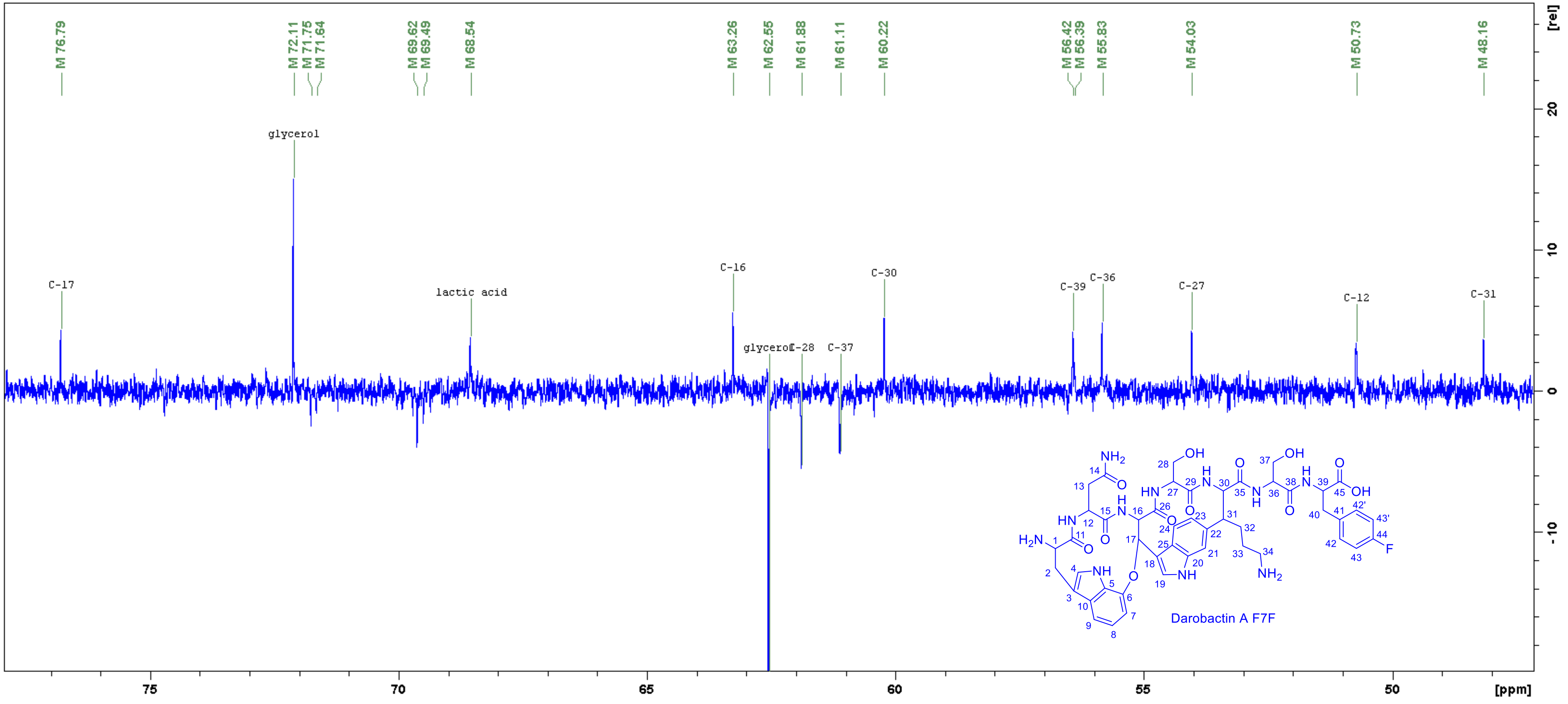

Figure S35: ^{13}C-NMR spectrum of darobactin A F7F (DEPTQ-135, D_2O, 176.1 MHz). For the measurement 3-(trimethylsilyl)propionic-2,2,3,3-d_4 acid sodium salt (TSPA) was used as external standard. Close-up in the range of 77.0– 48.0 ppm.

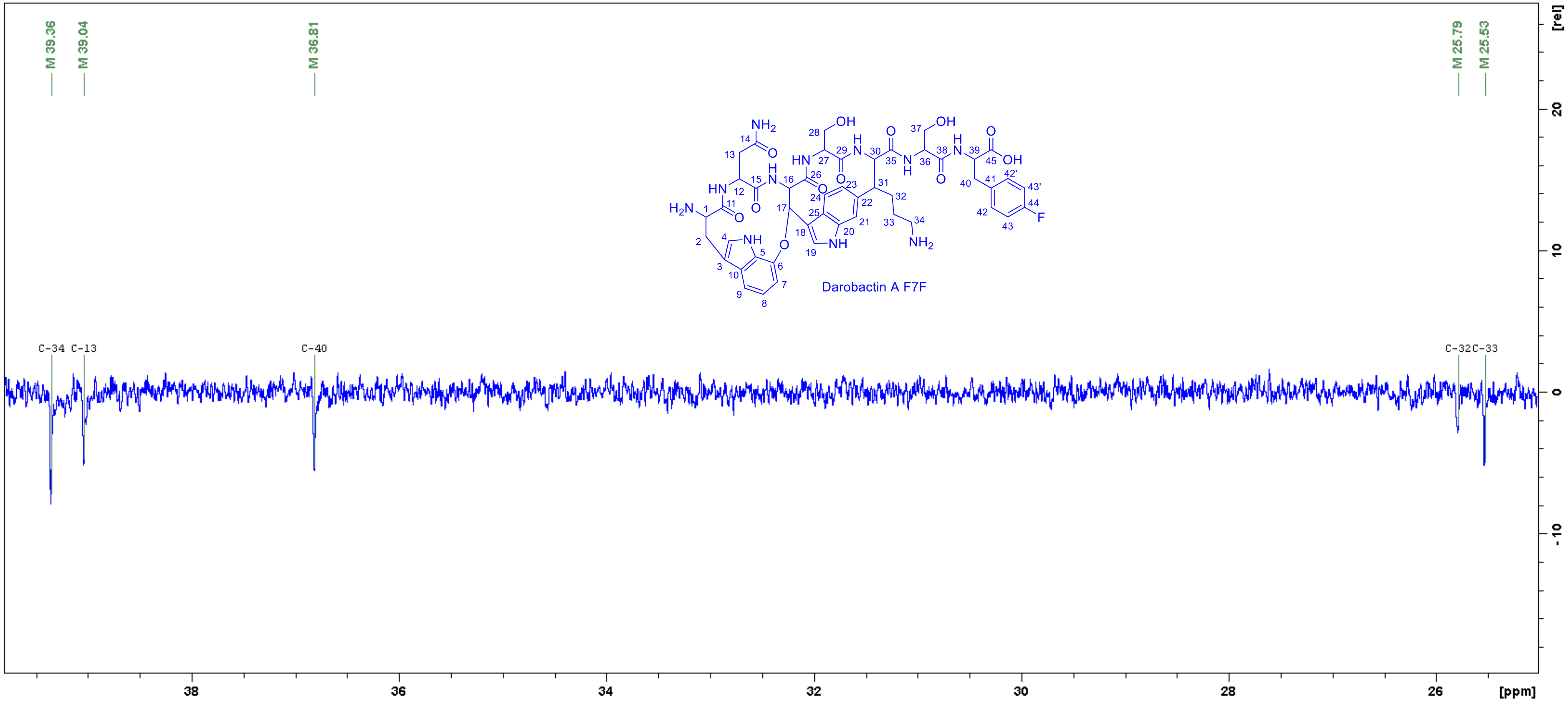

Figure S36: ^{13}C-NMR spectrum of darobactin A F7F (DEPTQ-135, D_2O, 176.1 MHz). For the measurement 3-(trimethylsilyl)propionic-2,2,3,3-d_4 acid sodium salt (TSPA) was used as external standard. Close-up in the range of 39.5– 25.5 ppm.

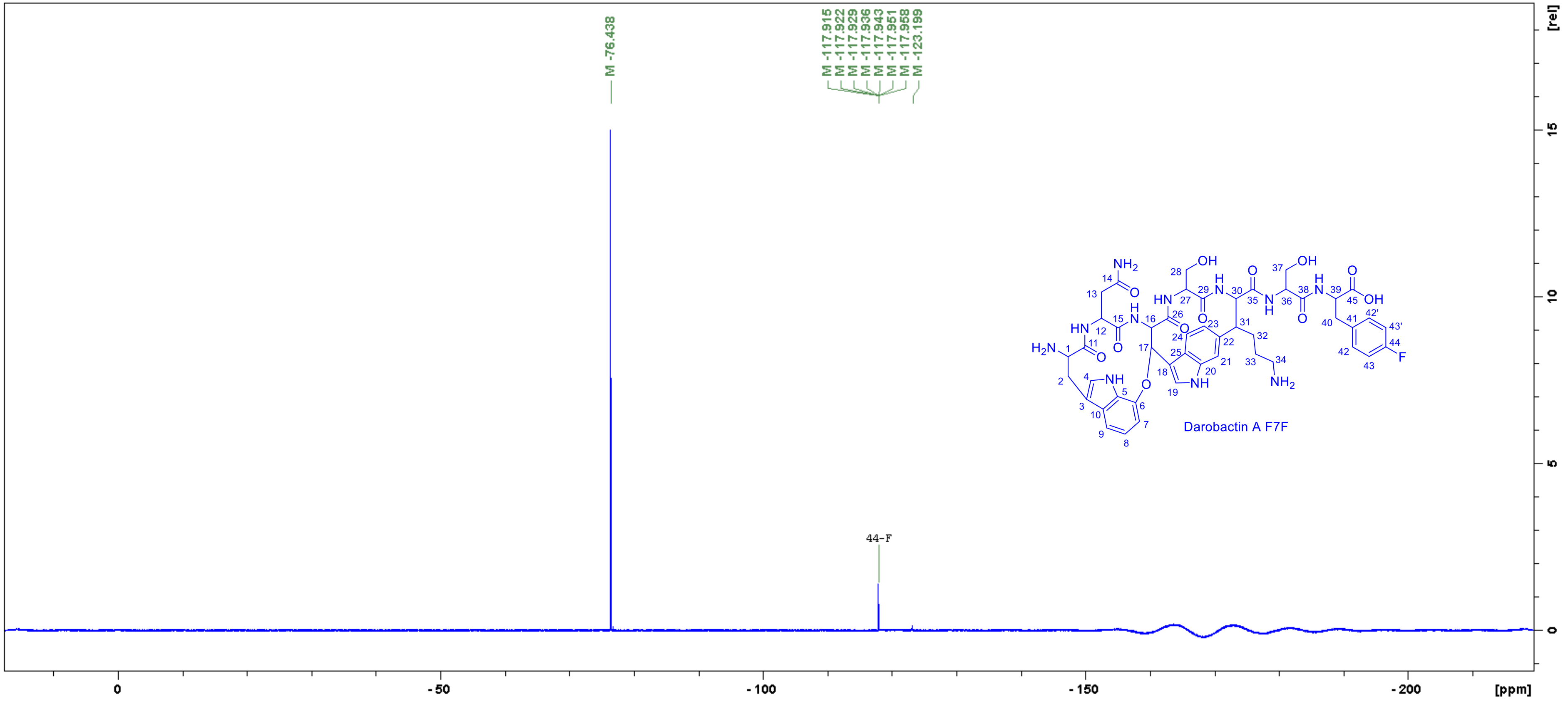

Figure S37: ^{19}F-NMR {/} spectrum of darobactin A F7F (D_2O, 658.9 MHz). For ^{19}F measurements α,α,α-trifluorotoluene served as an external standard. The uneven baseline between (-150) – (-200) ppm is an artifact caused by the employed NMR probe, which does not have a separate channel for detecting ^{19}F.

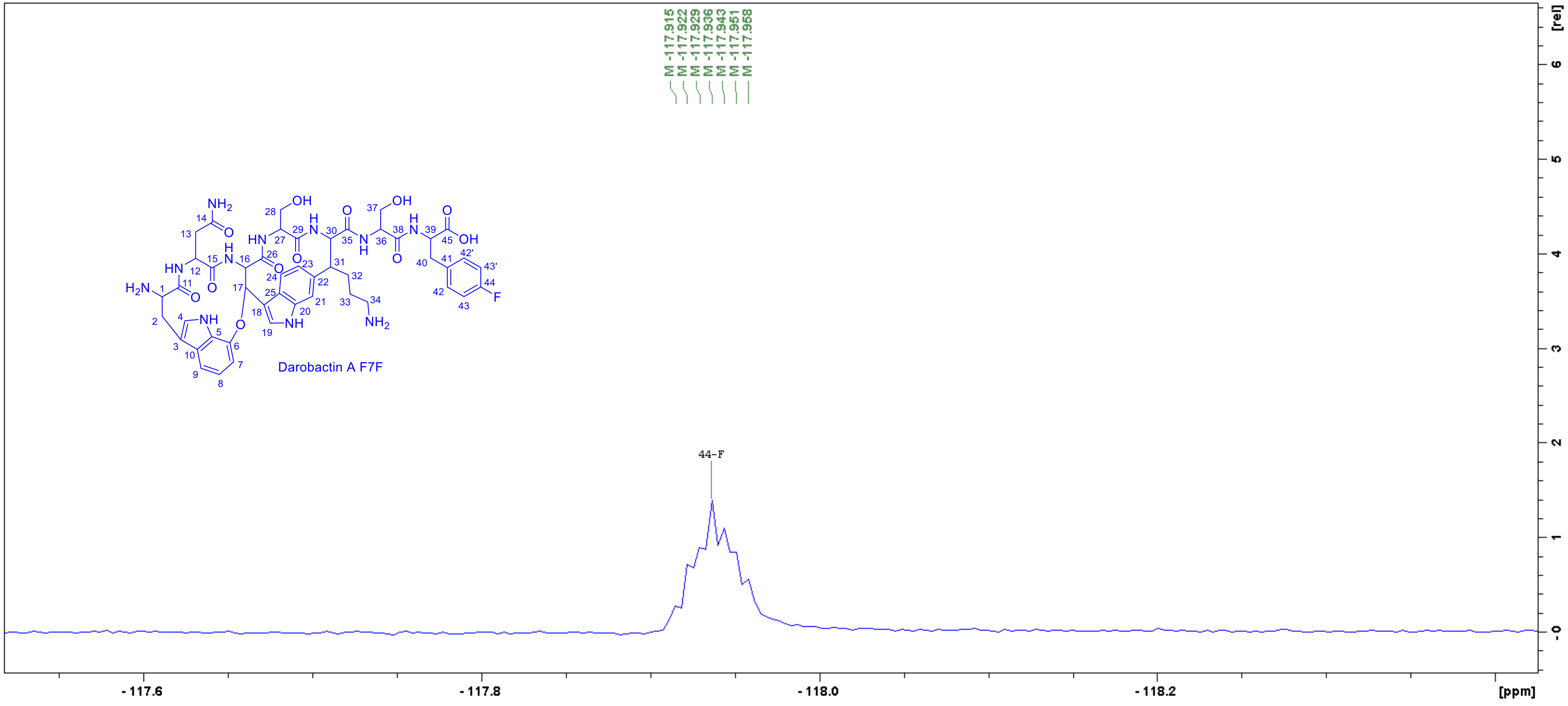

Figure S38: ^{19}F-NMR {/} spectrum of darobactin A F7F (D_2O, 658.9 MHz). For ^{19}F measurements α,α,α-trifluorotoluene served as an external standard. Close-up in the range of (-117.5) – (-118.4) ppm.

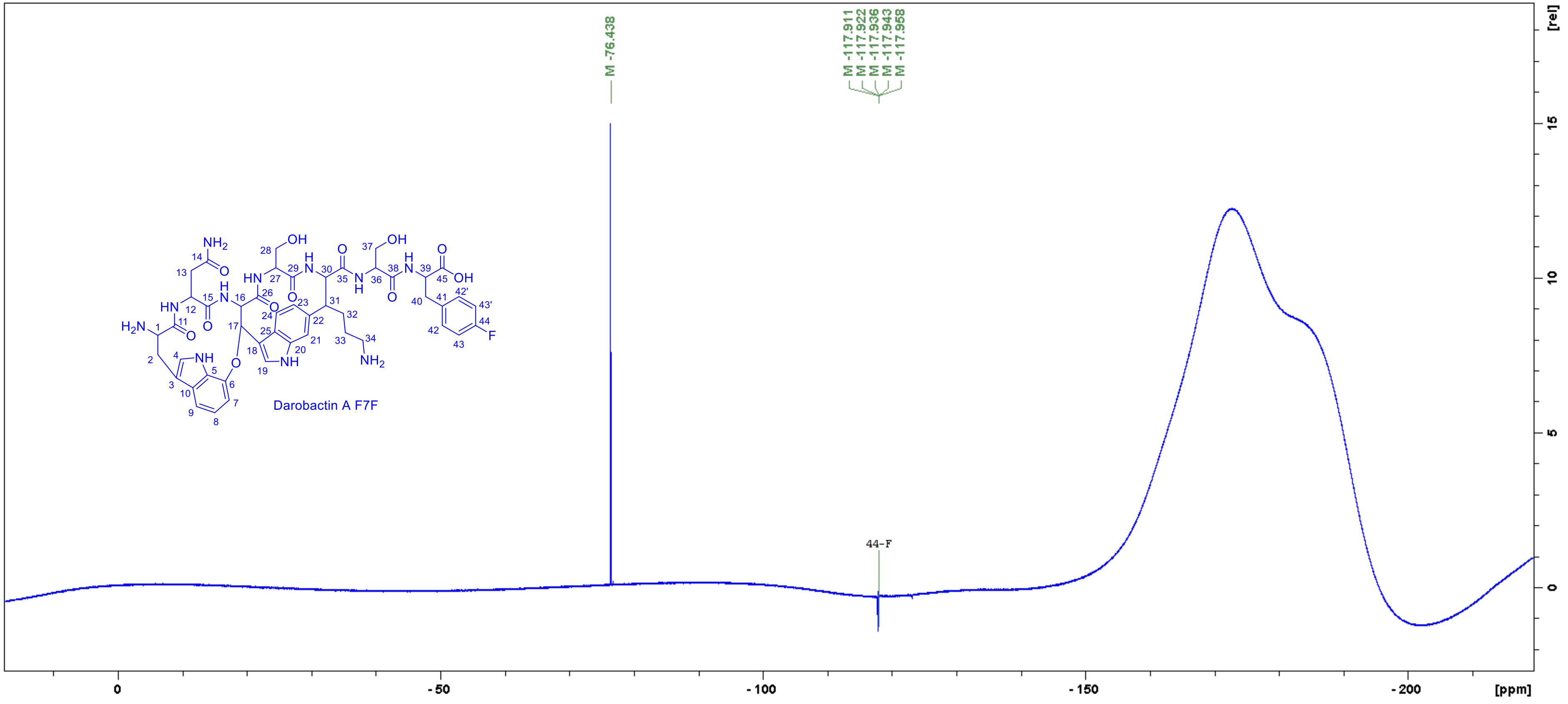

Figure S39: Proton-decoupled ^{19}F-NMR {H} spectrum of darobactin A F7F (D_2O, 658.9 MHz). For ^{19}F measurements α,α,α-trifluorotoluene served as an external standard. The signal between (-150) – (-200) ppm is an artifact caused by the employed NMR probe, which does not have a separate channel for detecting ^{19}F. The artifact is relatively intense, because the sample itself is quite dilute.

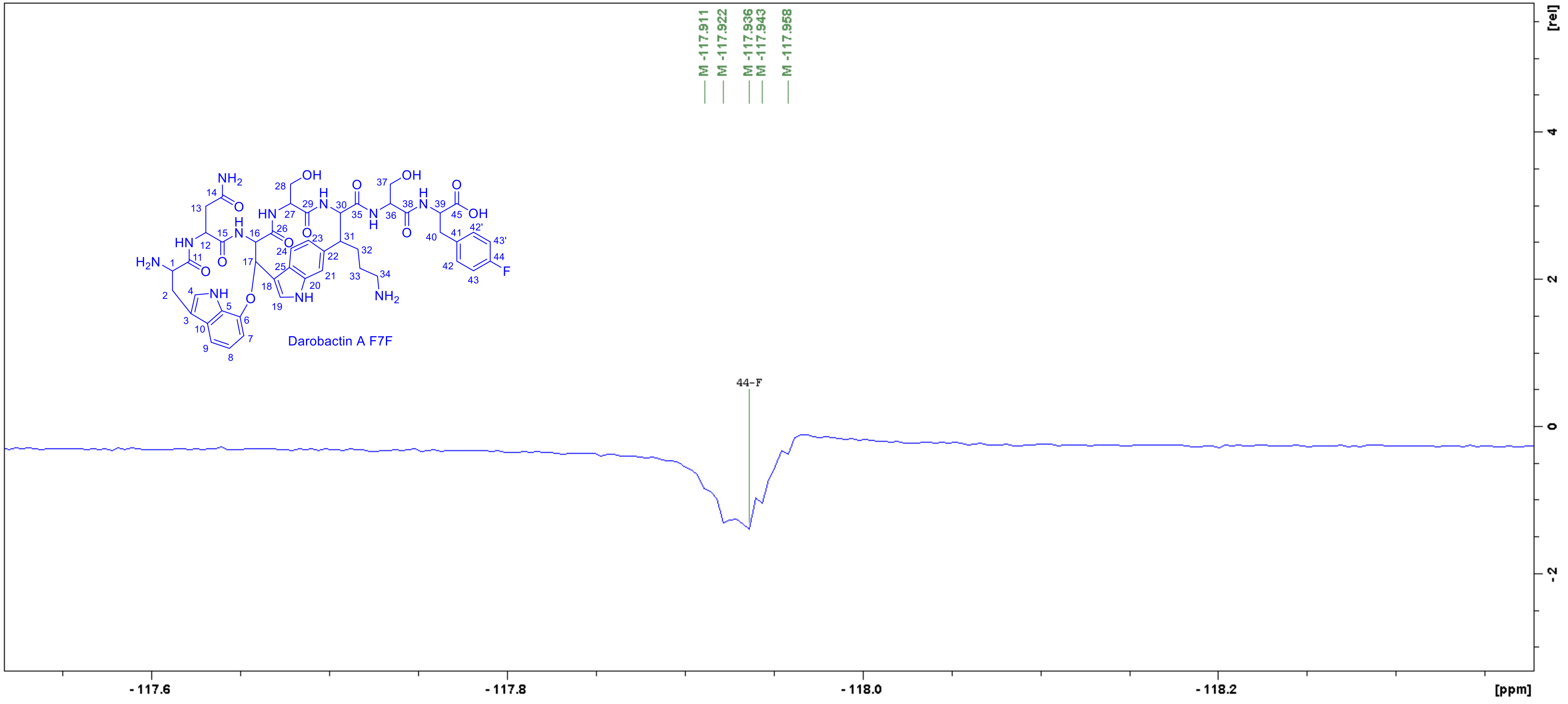

Figure S40: Proton-decoupled ^{19}F-NMR {H} spectrum of darobactin A F7F (D_2O, 658.9 MHz). For ^{19}F measurements α,α,α-trifluorotoluene served as an external standard. Close-up in the range of (-117.55) – (-118.35) ppm.

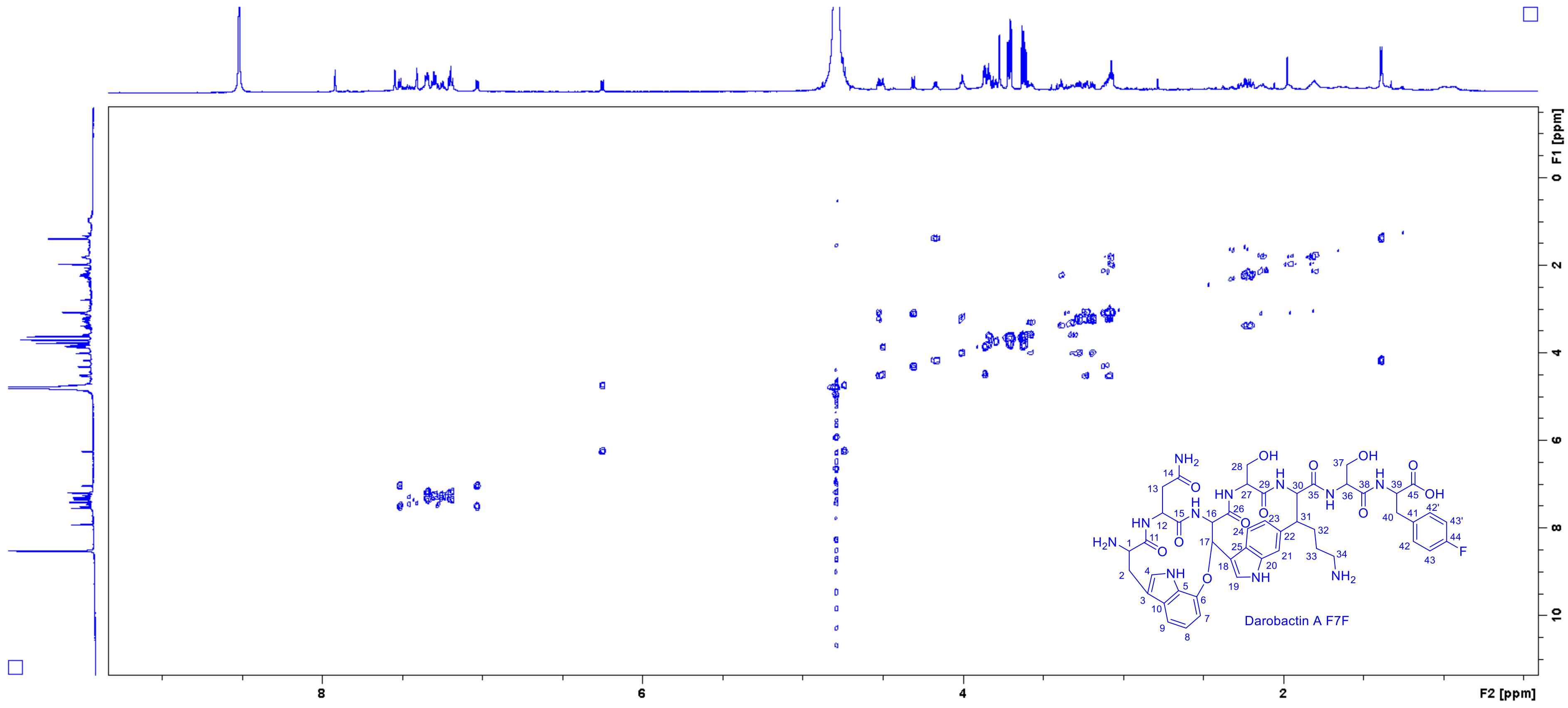

Figure S41: COSY spectrum of darobactin A F7F (D_2O, 700 MHz).

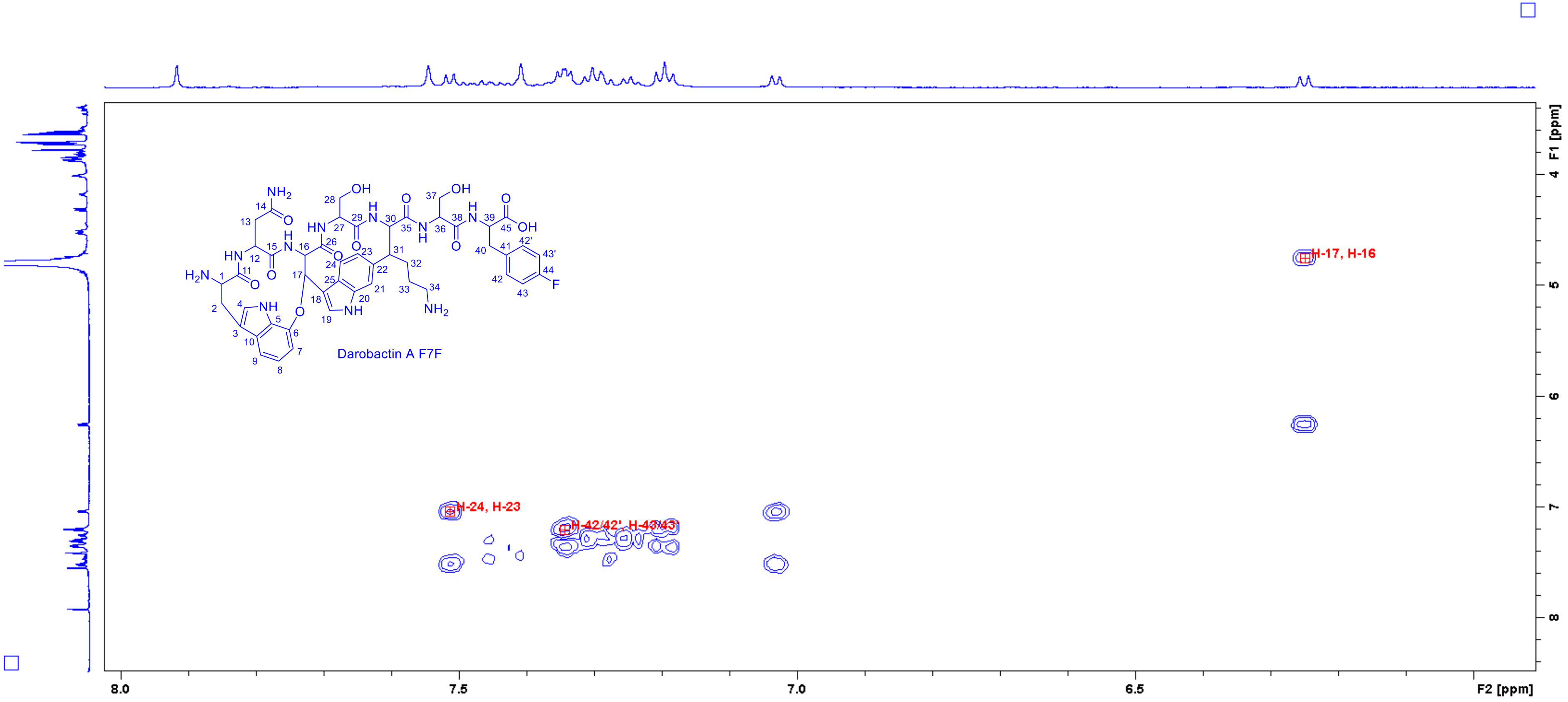

Figure S42: COSY spectrum of darobactin A F7F (D_2O, 700 MHz). Close-up in the region of 8.0 – 6.0 ppm (F2 axis) and 8.4 – 3.4 ppm (F1 axis) with peak assignments.

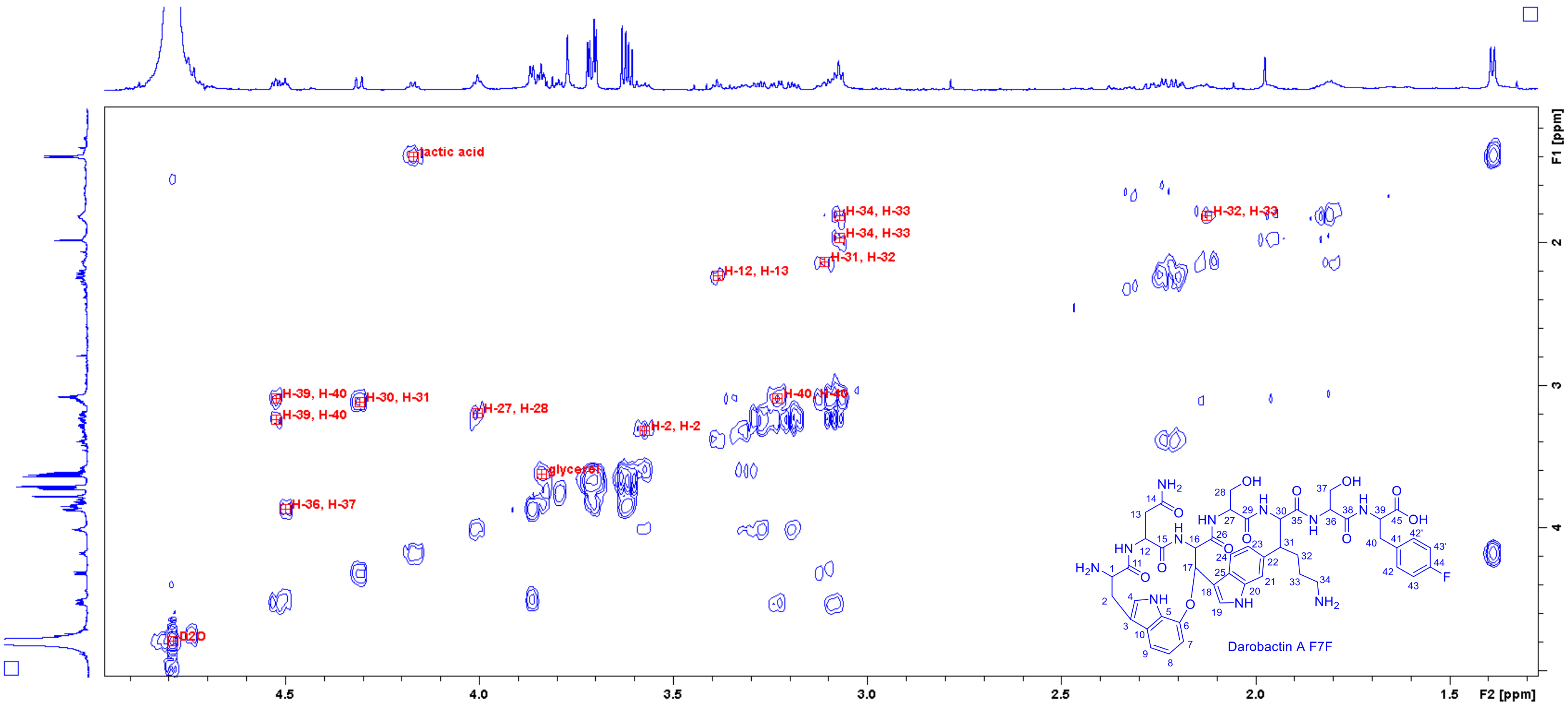

Figure S43: COSY spectrum of darobactin A F7F (D_2O, 700 MHz). Close-up in the region of 4.9 – 1.3 ppm (F2 axis) and 5.0 – 1.2 ppm (F1 axis) with peak assignments.

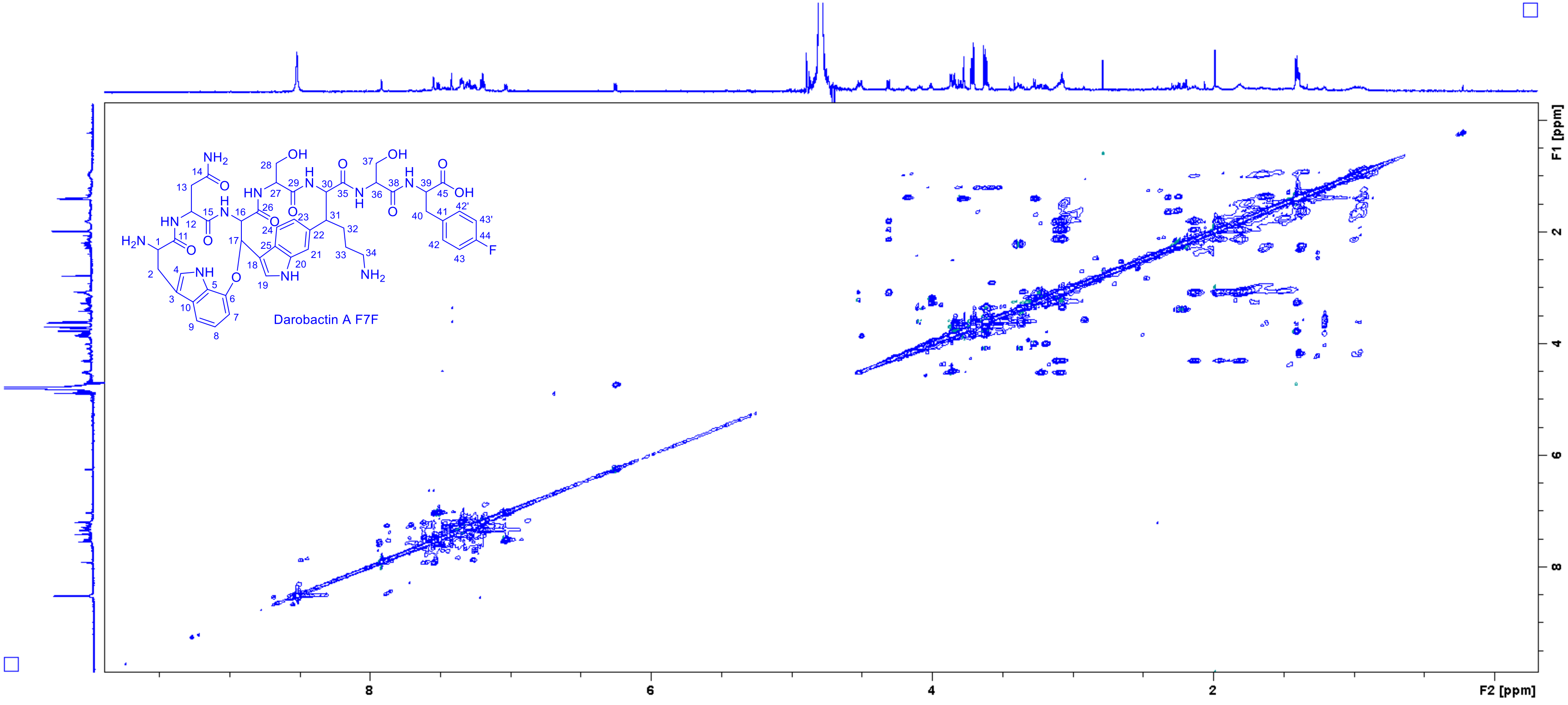

Figure S44: TOCSY spectrum of darobactin A F7F (D_2O, 700 MHz), measured with H_2O suppression.

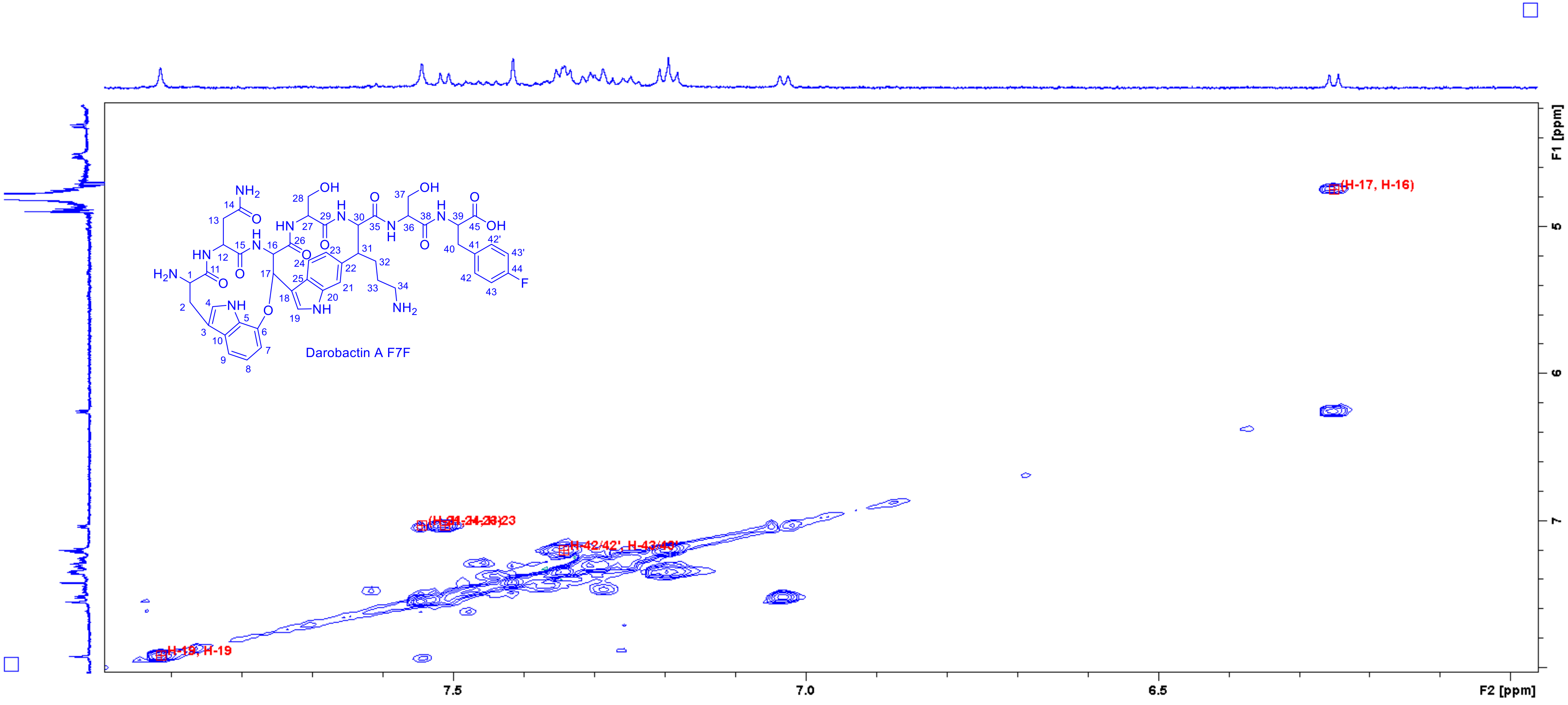

Figure S45: TOCSY spectrum of darobactin A F7F (D_2O, 700 MHz), measured with H_2O suppression. Close-up in the region of 7.9 – 6.0 ppm (F2 axis) and 8.0 – 4.2 ppm (F1 axis) with peak assignments.

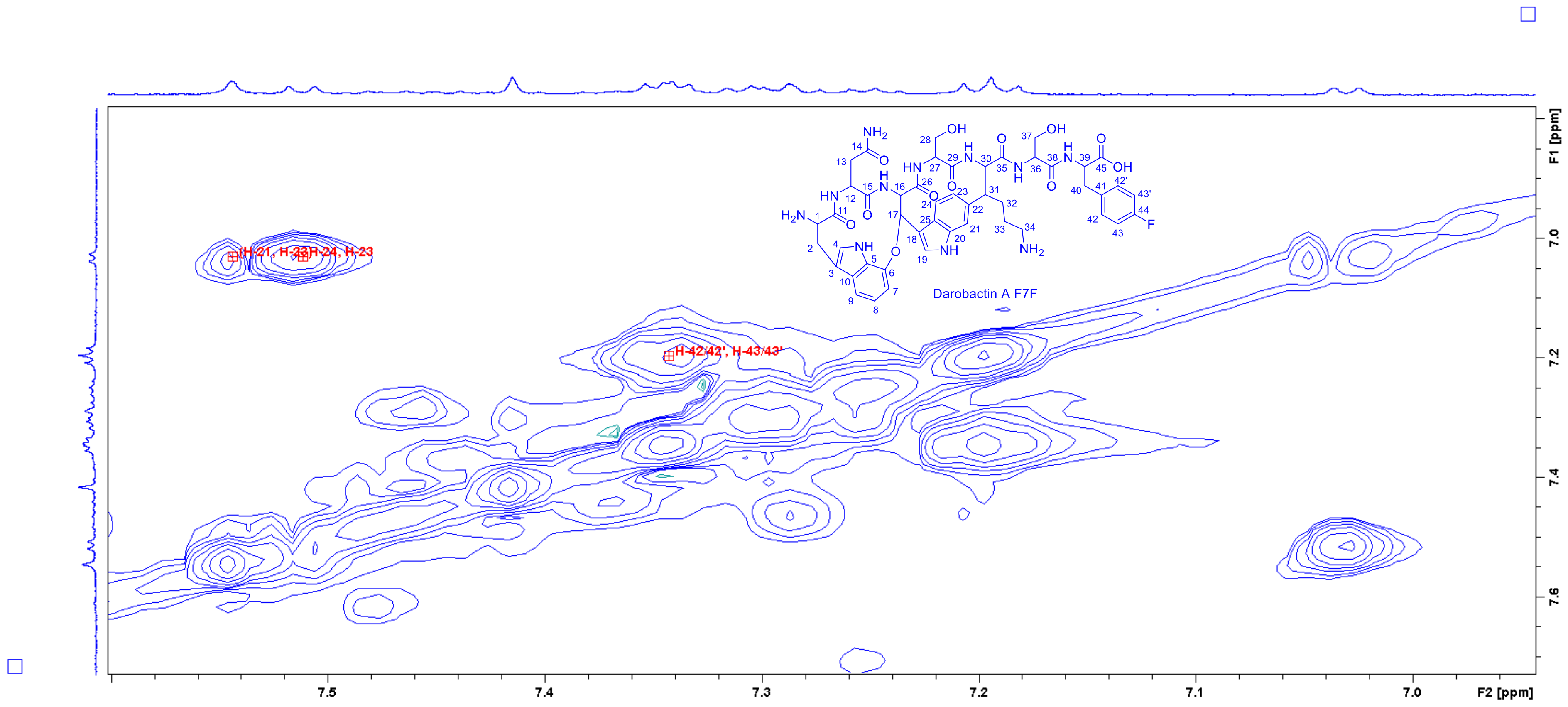

Figure S46: TOCSY spectrum of darobactin A F7F (D_2O, 700 MHz), measured with H_2O suppression. Close-up in the region of 7.60 – 6.96 ppm (F2 axis) and 7.7 – 6.8 ppm (F1 axis) with peak assignments.

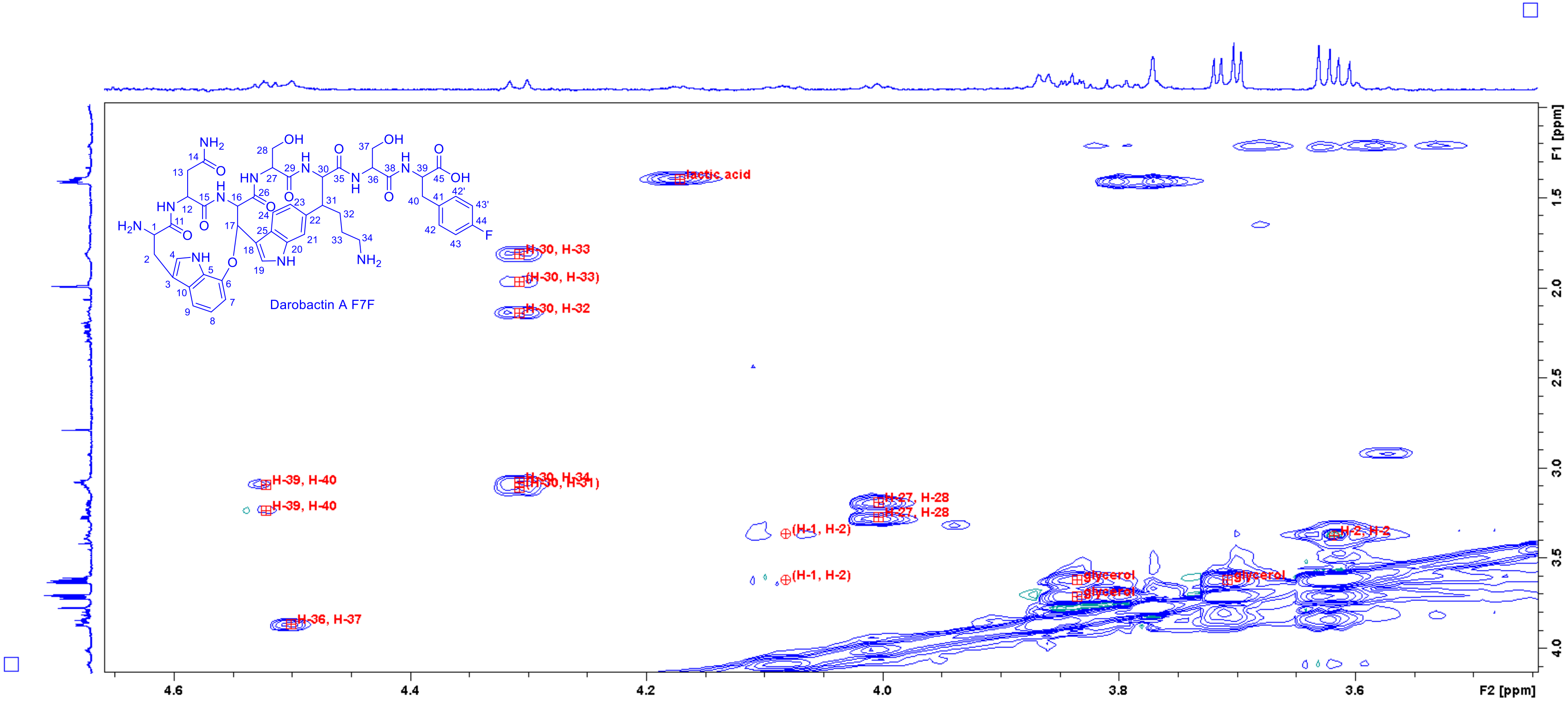

Figure S47: TOCSY spectrum of darobactin A F7F (D_2O, 700 MHz), measured with H_2O suppression. Close-up in the region of 4.65 – 3.45 ppm (F2 axis) and 4.1 – 1.0 ppm (F1 axis) with peak assignments.

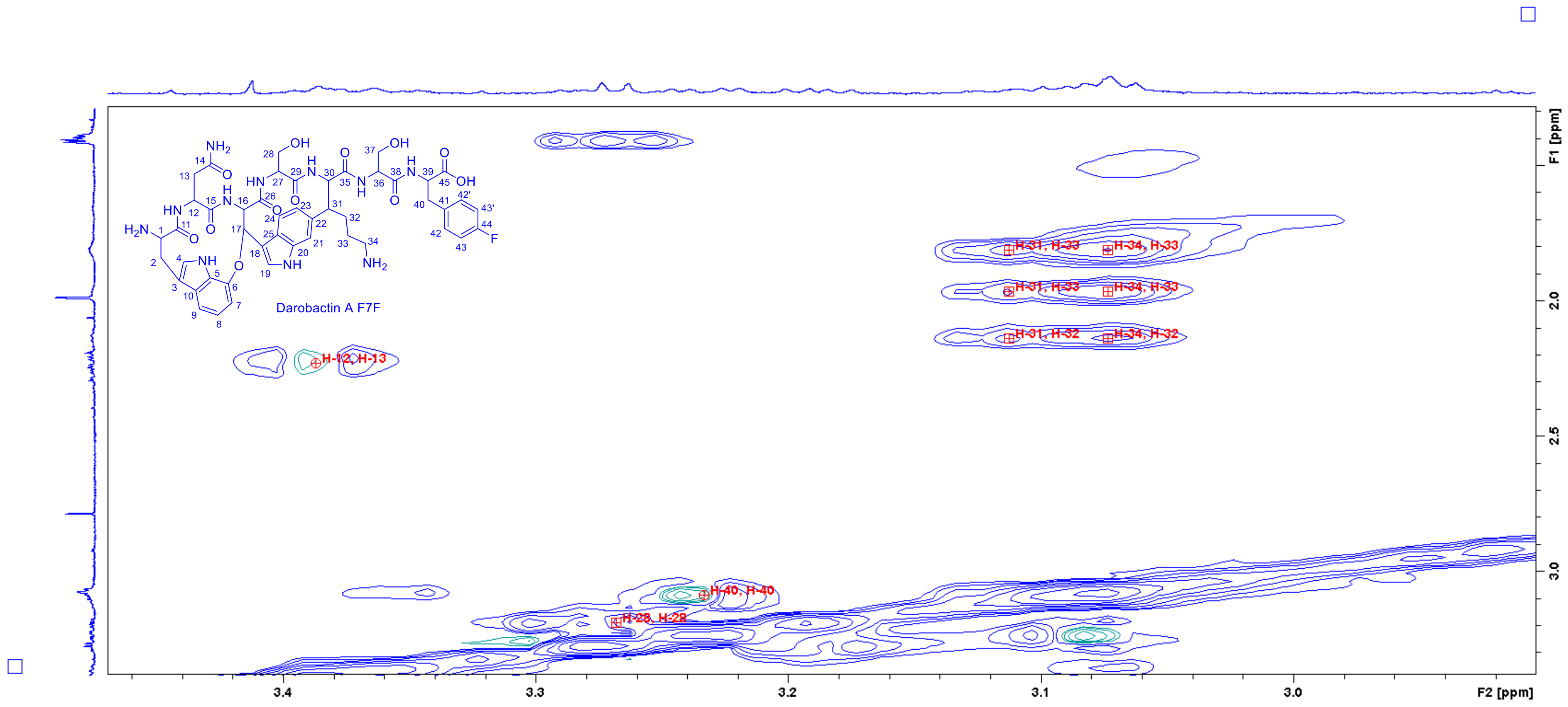

Figure S48: TOCSY spectrum of darobactin A F7F (D_2O, 700 MHz), measured with H_2O suppression. Close-up in the region of 3.46 – 2.92 ppm (F2 axis) and 3.3 – 1.3 ppm (F1 axis) with peak assignments.

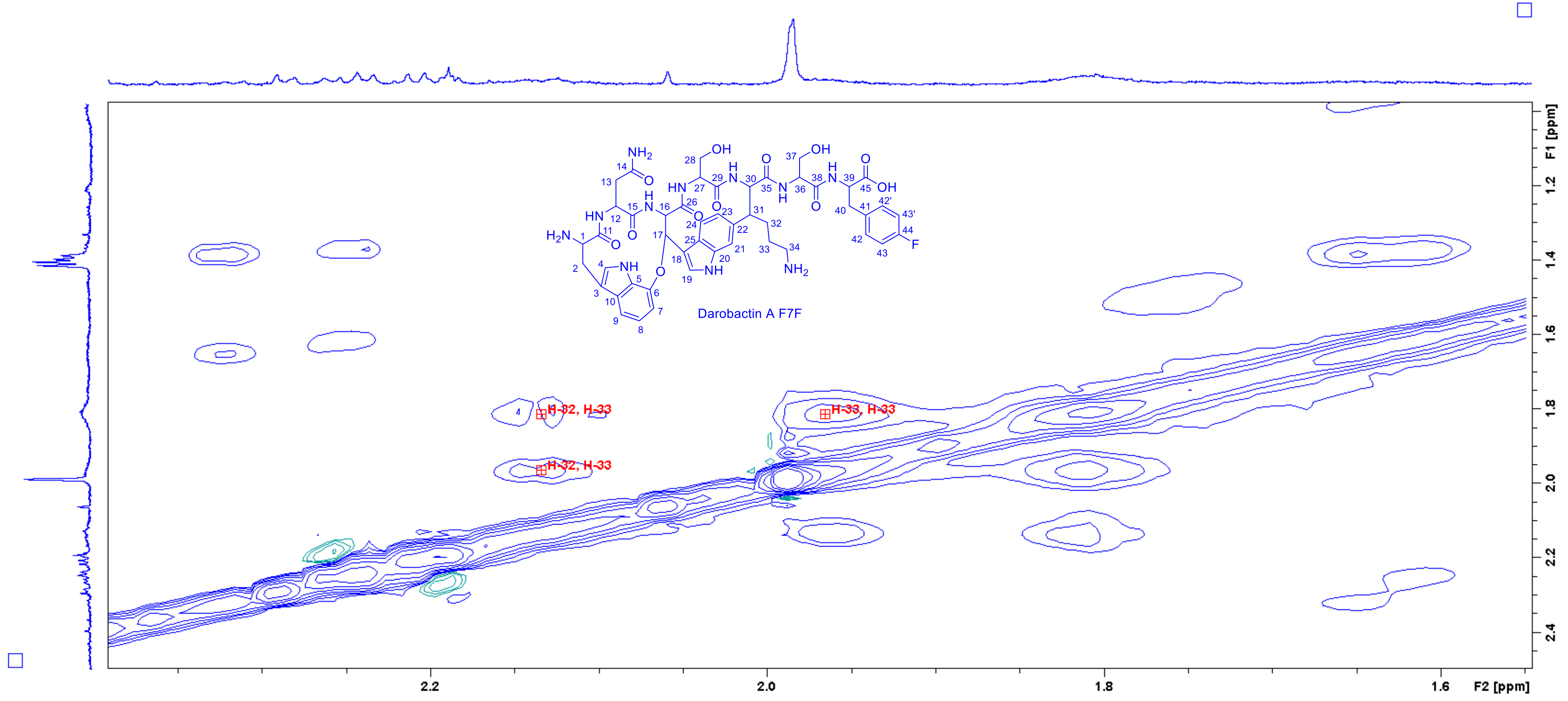

Figure S49: TOCSY spectrum of darobactin A F7F (D_2O, 700 MHz), measured with H_2O suppression. Close-up in the region of 2.35 – 1.55 ppm (F2 axis) and 2.45 – 1.00 ppm (F1 axis) with peak assignments.

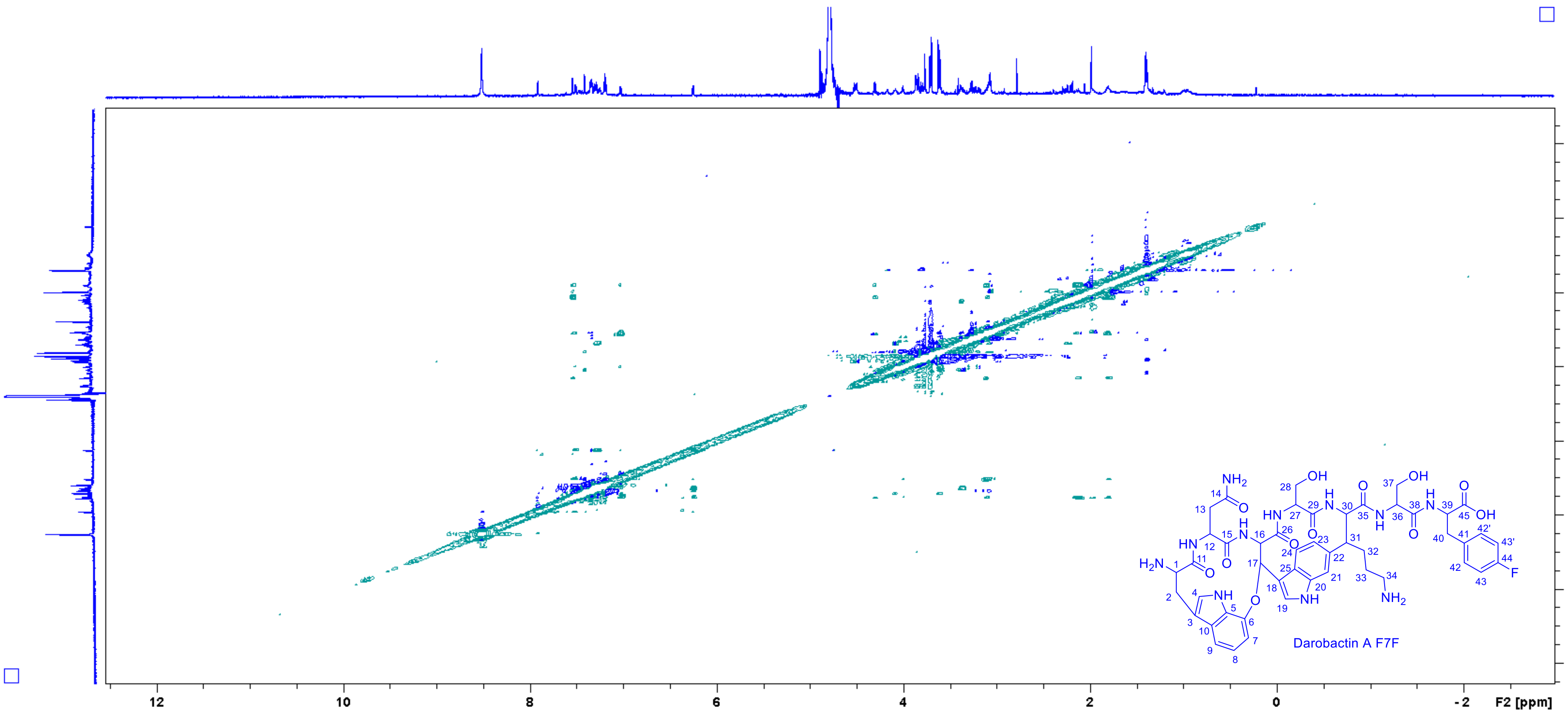

Figure S50: NOESY spectrum of darobactin A F7F (D_2O, 700 MHz), measured with H_2O suppression.

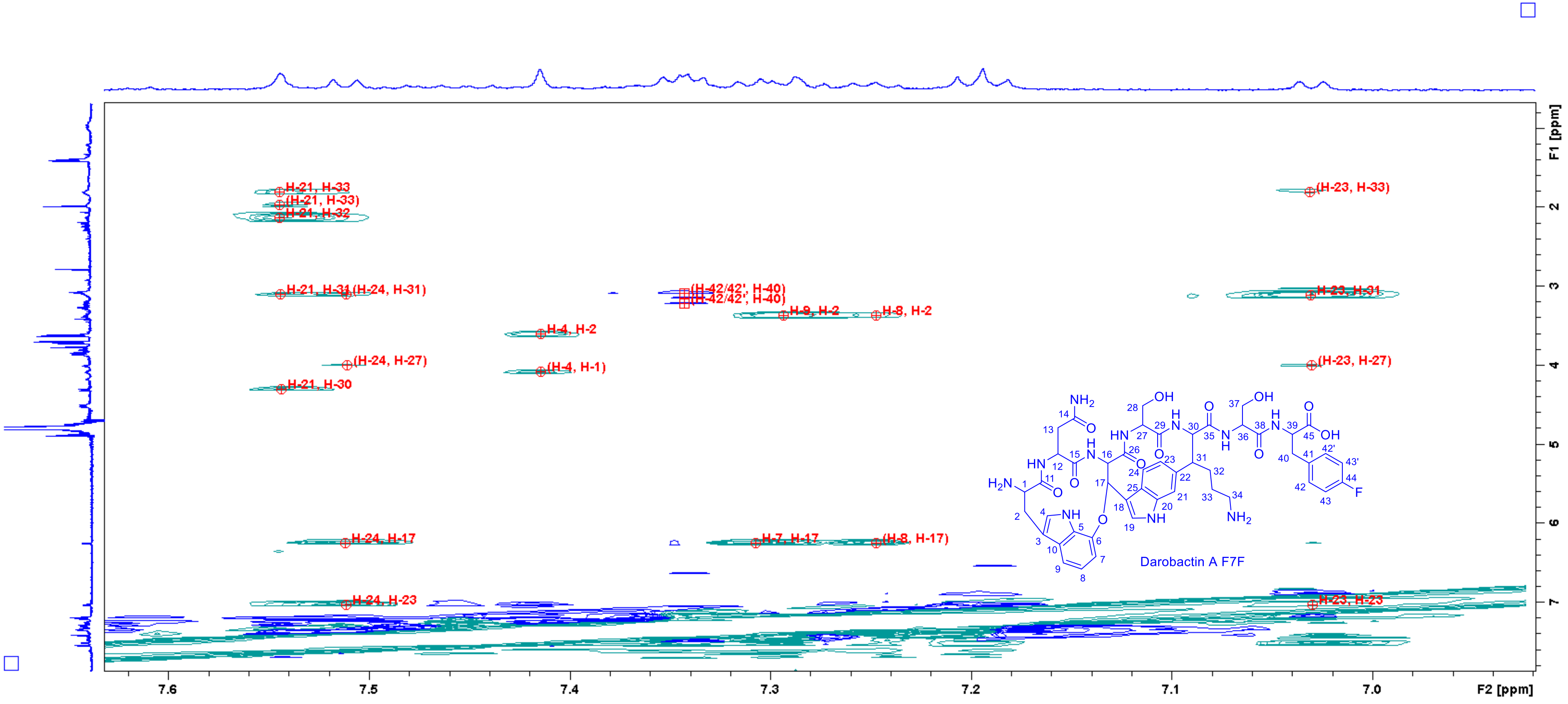

Figure S51: NOESY spectrum of darobactin A F7F (D_2O, 700 MHz), measured with H_2O suppression. Close-up in the region of 7.62 – 6.92 ppm (F2 axis) and 7.8 – 0.8 ppm (F1 axis) with peak assignments.

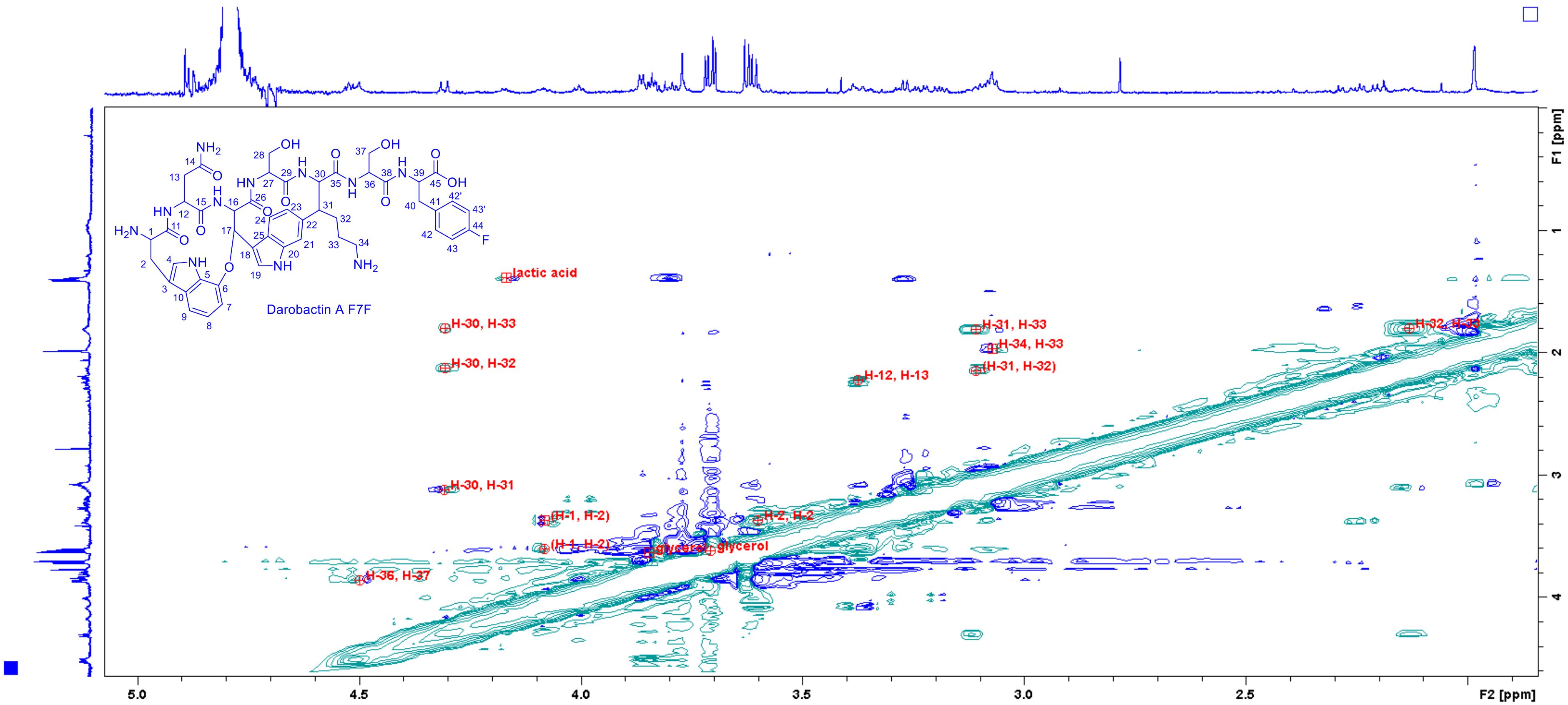

Figure S52: NOESY spectrum of darobactin A F7F (D_2O, 700 MHz), measured with H_2O suppression. Close-up in the region of 5.0 – 1.9 ppm (F2 axis) and 4.6 – 0.0 ppm (F1 axis) with peak assignments.

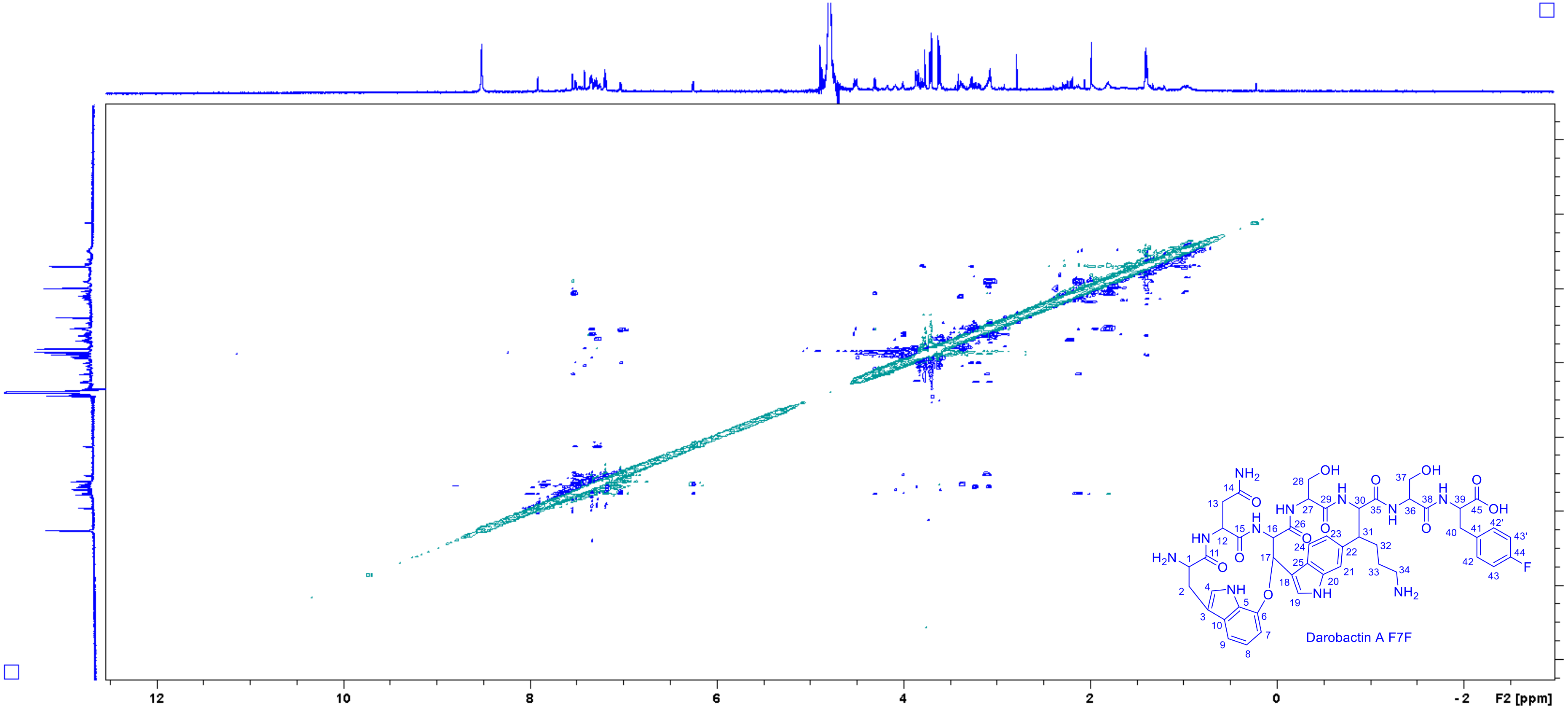

Figure S53: ROESY spectrum of darobactin A F7F (D_2O, 700 MHz), measured with H_2O suppression.

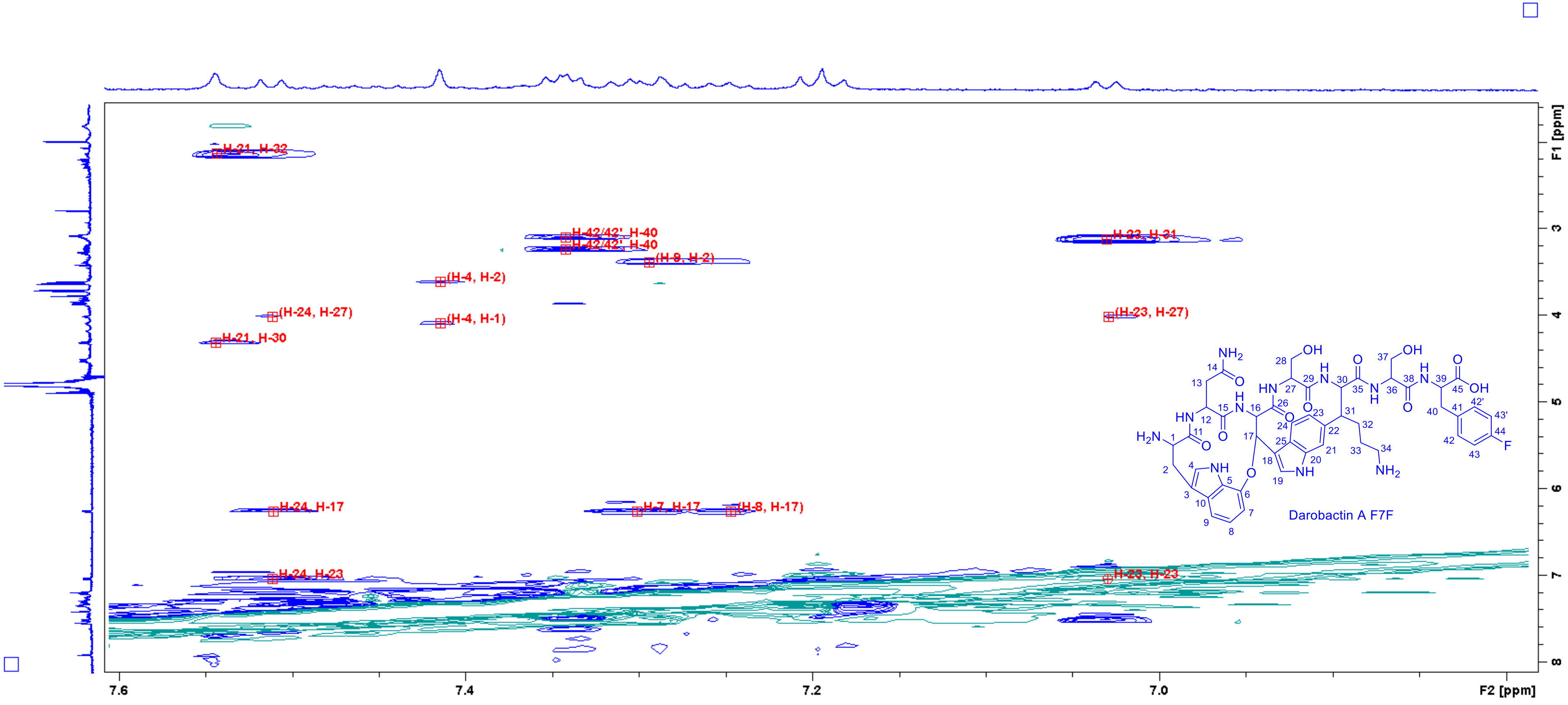

Figure S54: ROESY spectrum of darobactin A F7F (D_2O, 700 MHz), measured with H_2O suppression. Close-up in the region of 7.6 – 6.8 ppm (F2 axis) and 8.0 – 1.6 ppm (F1 axis) with peak assignments.

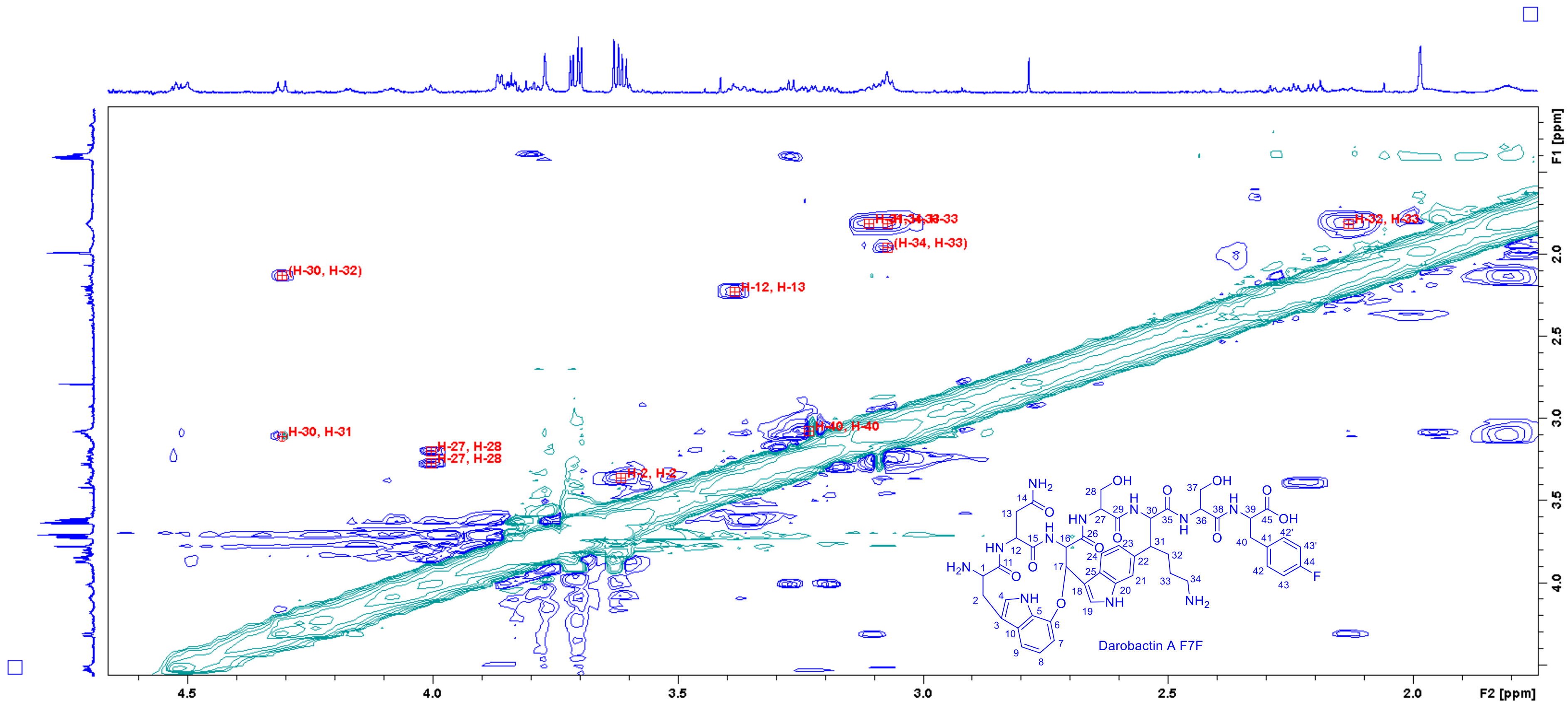

Figure S55: ROESY spectrum of darobactin A F7F (D_2O, 700 MHz), measured with H_2O suppression. Close-up in the region of 4.6 – 1.8 ppm (F2 axis) and 4.5 – 1.2 ppm (F1 axis) with peak assignments.

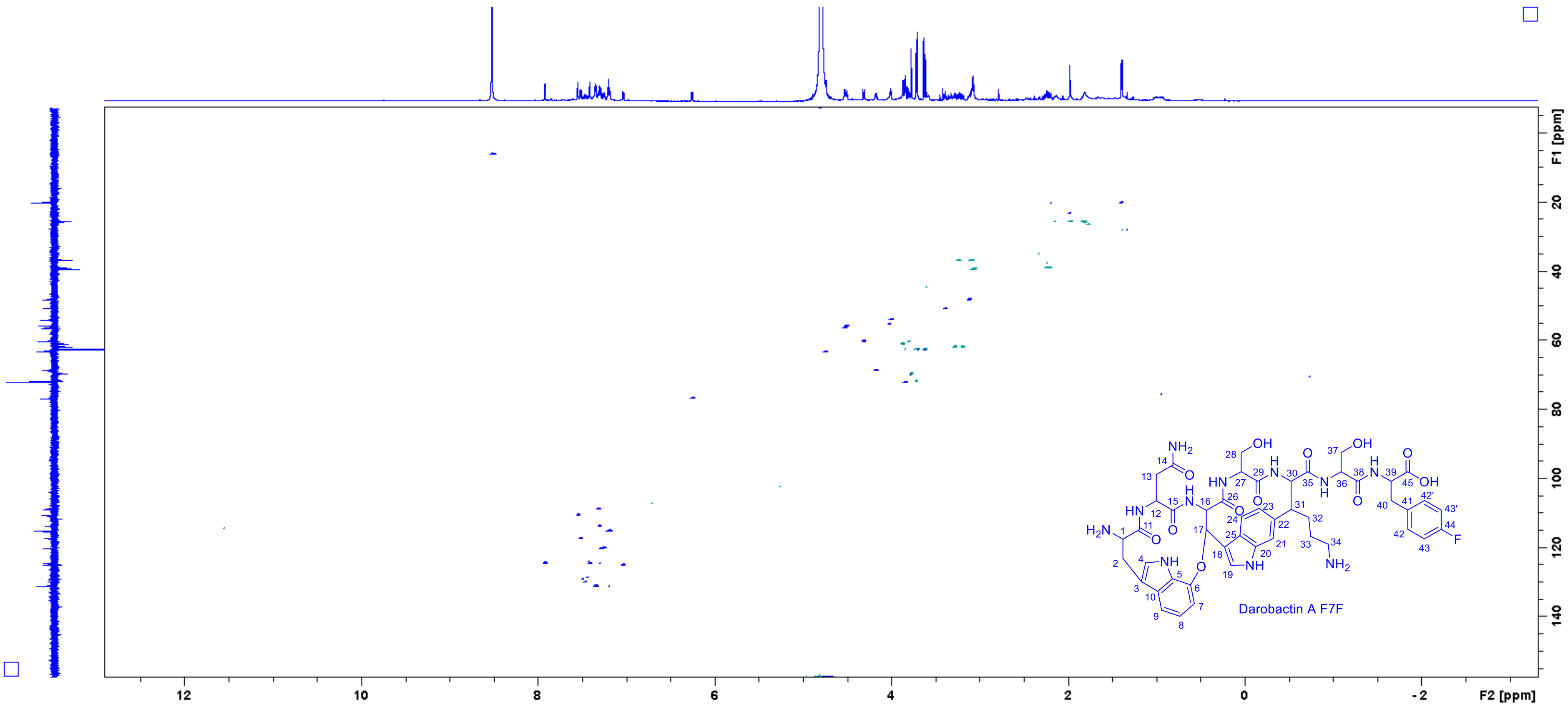

Figure S56: HSQC spectrum of darobactin A F7F (concentrated sample, D_2O, ^1H: 700 MHz, ^{13}C: 176.1 MHz), measured with non-uniform sampling. For easier peak identification the DEPTQ-135 experiment is shown on the F1 axis.

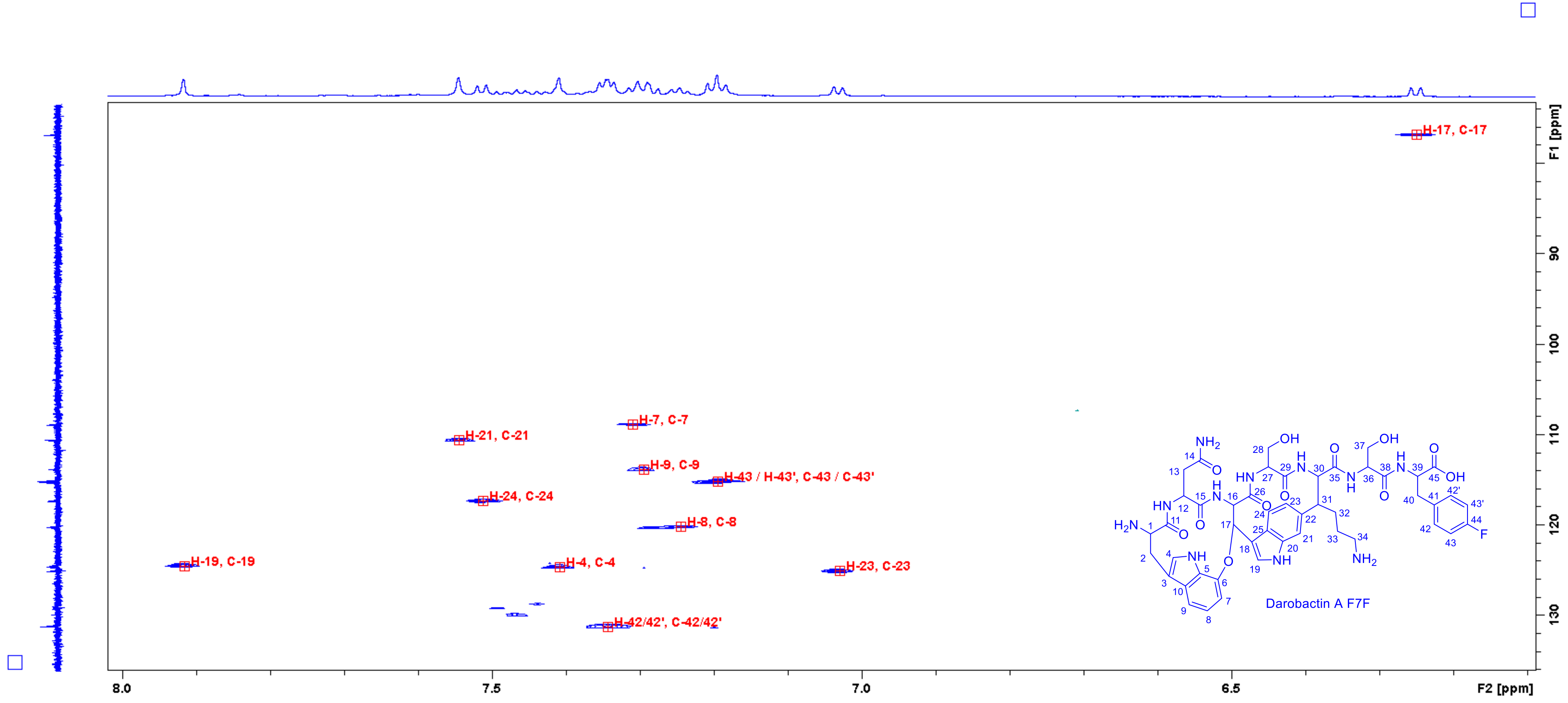

Figure S57: HSQC spectrum of darobactin A F7F (concentrated sample, D_2O, ^1H: 700 MHz, ^{13}C: 176.1 MHz), measured with non-uniform sampling. Close-up in the region of 8.0 – 6.1 ppm (F2 axis) and 136.0 – 74.0 ppm (F1 axis) with peak assignments. For easier peak identification the DEPTQ-135 experiment is shown on the F1 axis.

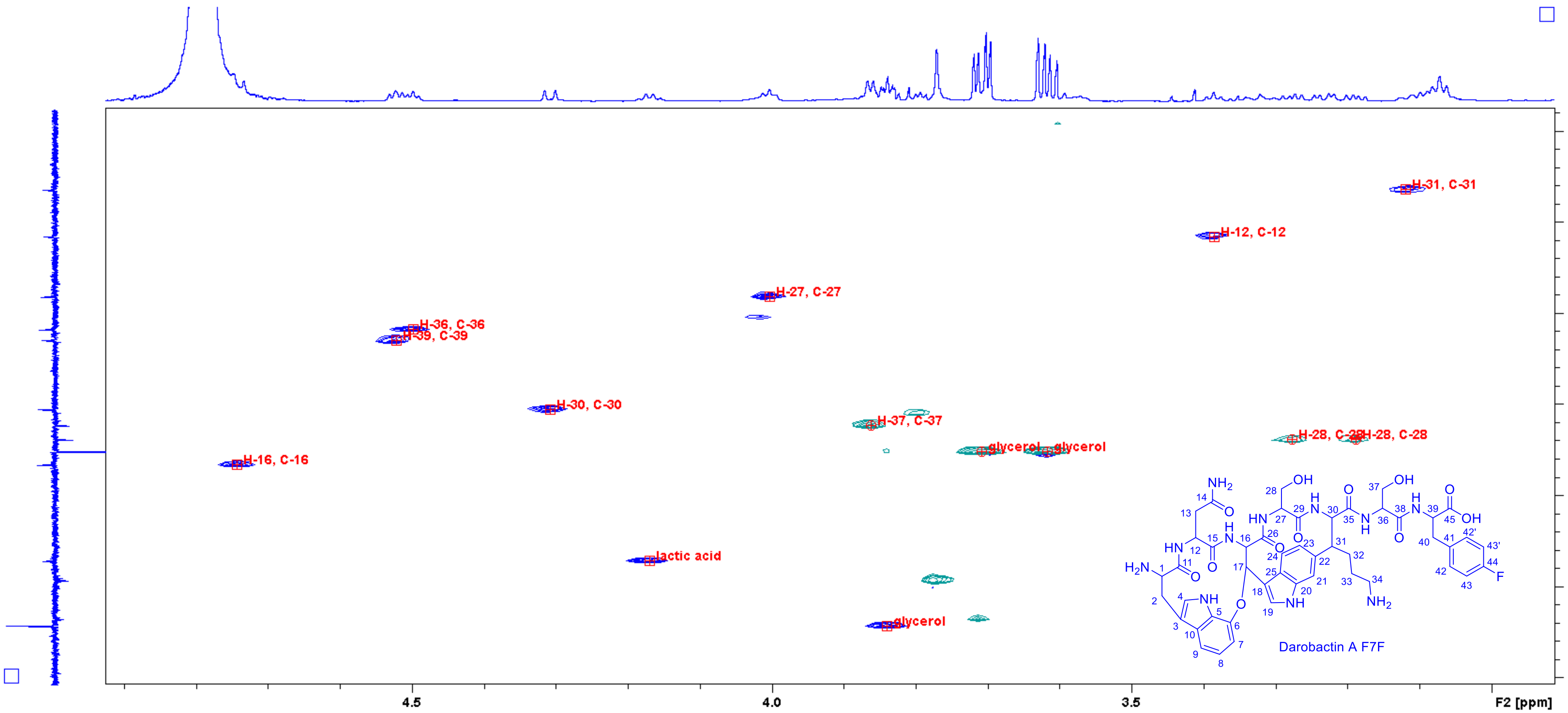

Figure S58: HSQC spectrum of darobactin A F7F (concentrated sample, D_2O, ^1H: 700 MHz, ^{13}C: 176.1 MHz), measured with non-uniform sampling. Close-up in the region of 4.9 – 3.0 ppm (F2 axis) and 75.0 – 44.0 ppm (F1 axis) with peak assignments. For easier peak identification the DEPTQ-135 experiment is shown on the F1 axis.

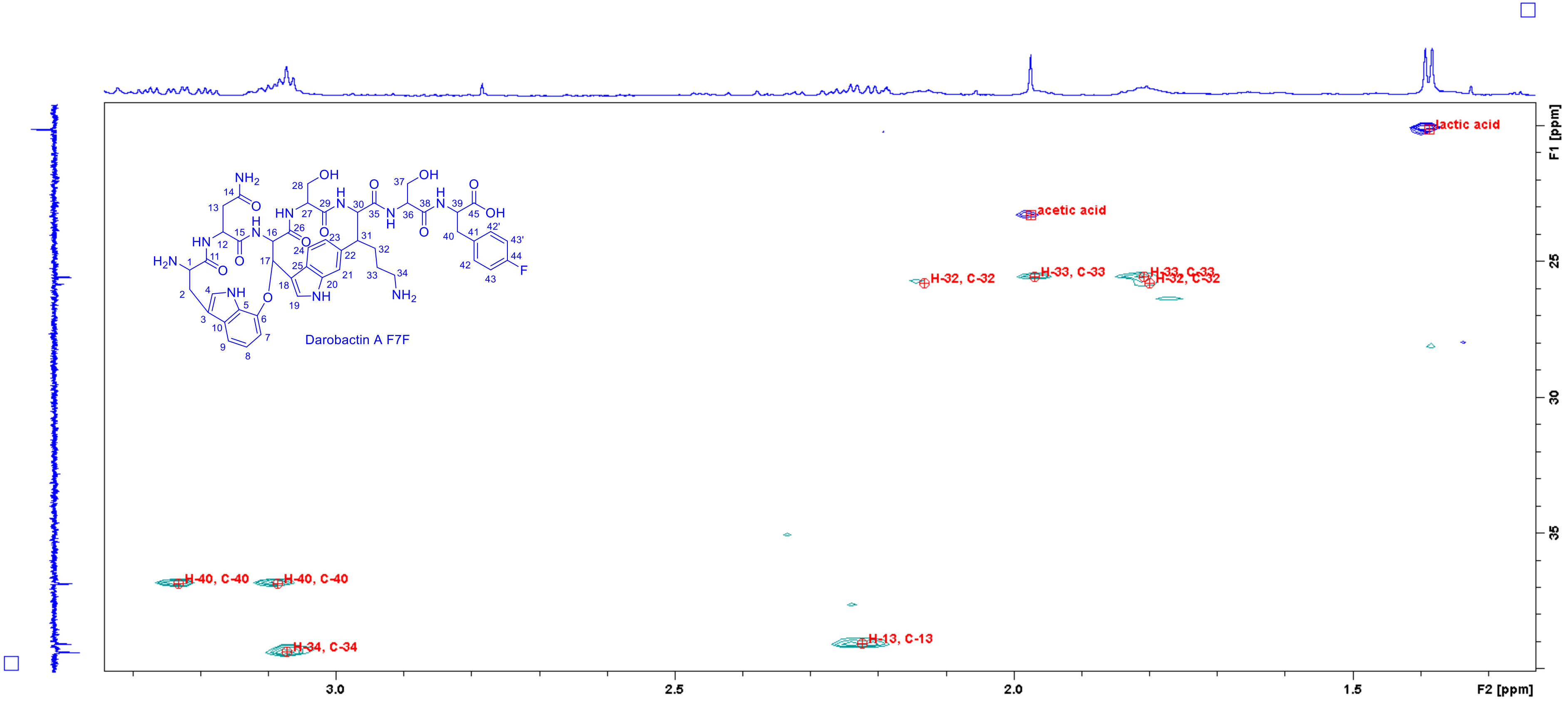

Figure S59: HSQC spectrum of darobactin A F7F (concentrated sample, D_2O, ^1H: 700 MHz, ^{13}C: 176.1 MHz), measured with non-uniform sampling. Close-up in the region of 3.3 – 1.3 ppm (F2 axis) and 40.0 – 20.0 ppm (F1 axis) with peak assignments. For easier peak identification the DEPTQ-135 experiment is shown on the F1 axis.

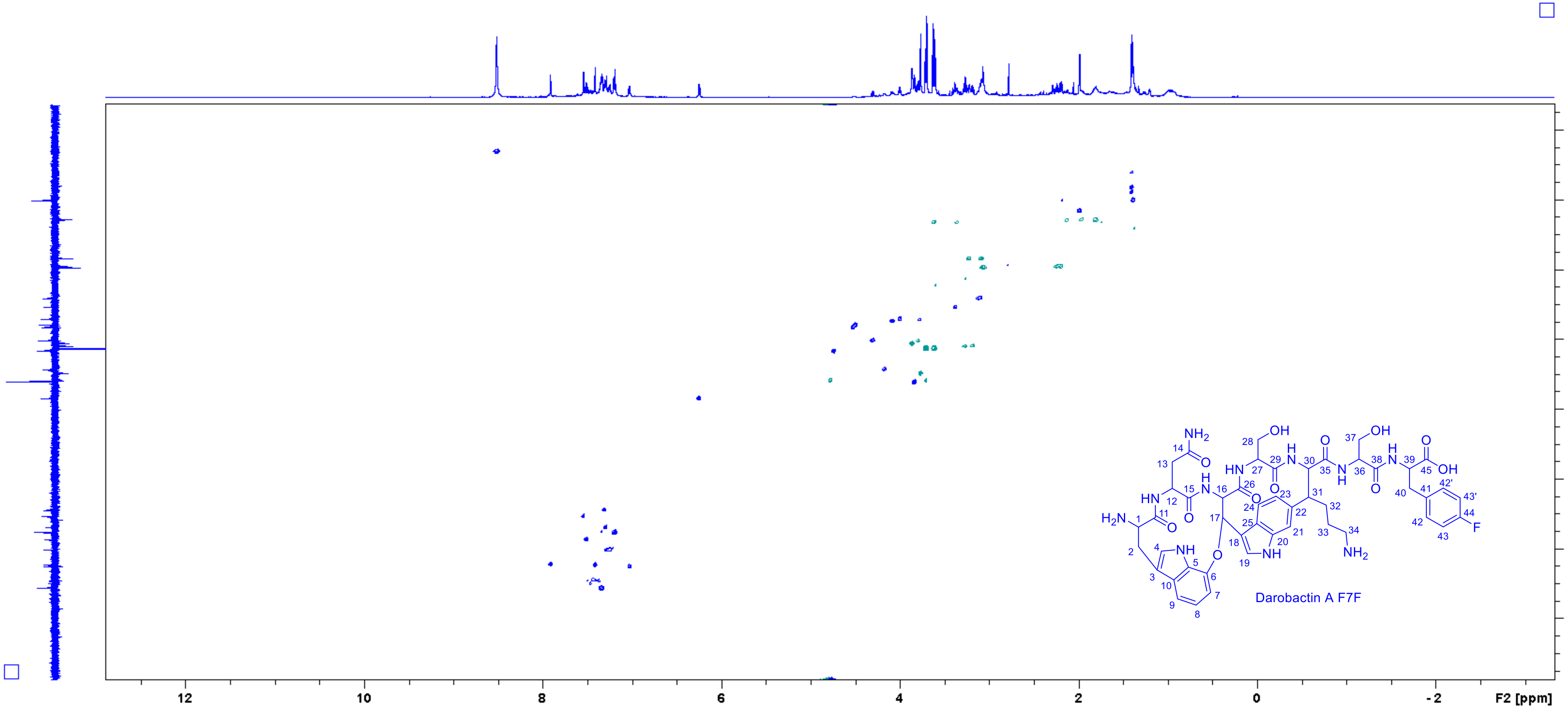

Figure S60: HSQC spectrum of darobactin A F7F (dilute sample, D_2O, ^1H: 700 MHz, ^{13}C: 176.1 MHz), measured with H_2O suppression. For easier peak identification the DEPTQ-135 experiment is shown on the F1 axis.

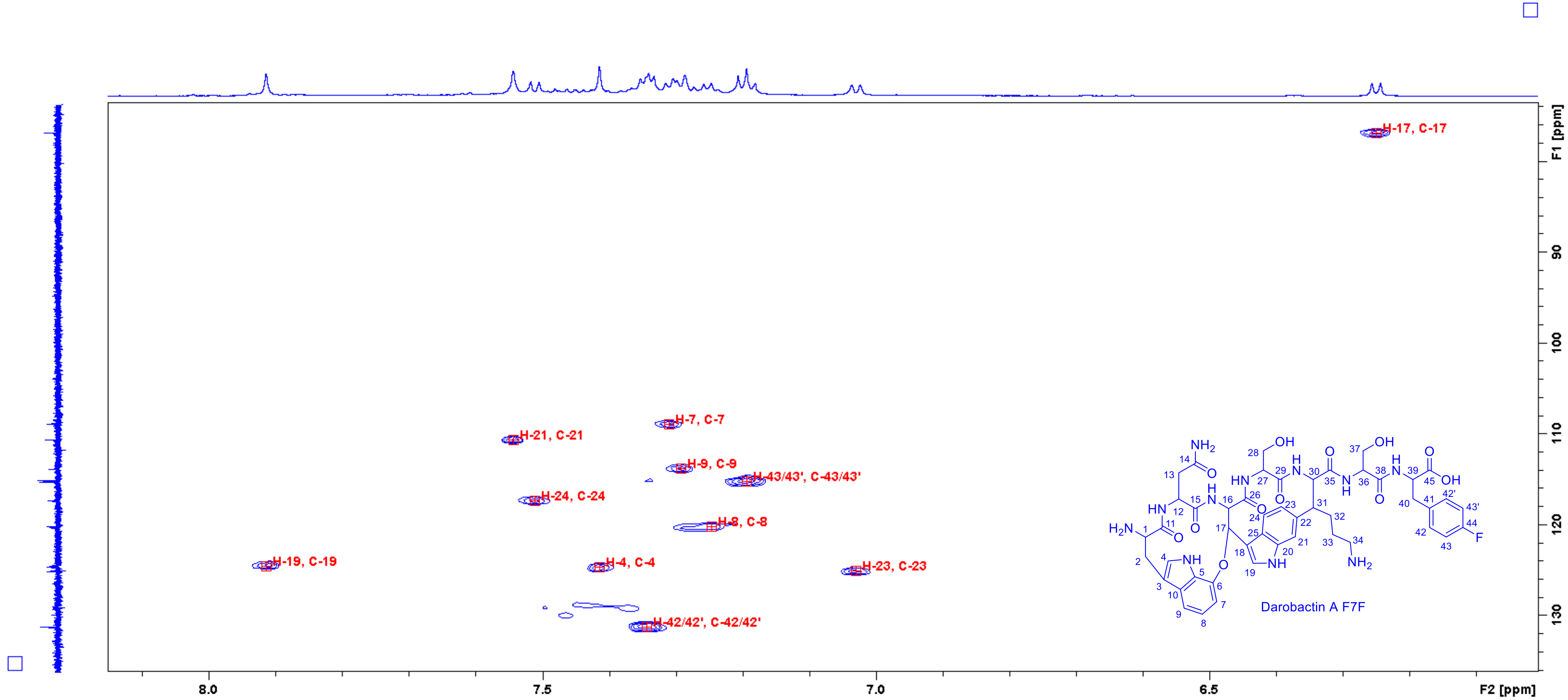

Figure S61: HSQC spectrum of darobactin A F7F (dilute sample, D_2O, ^1H: 700 MHz, ^{13}C: 176.1 MHz), measured with H_2O suppression. Close-up in the region of 8.1 – 6.1 ppm (F2 axis) and 136.0 – 74.0 ppm (F1 axis) with peak assignments. For easier peak identification the DEPTQ-135 experiment is shown on the F1 axis.

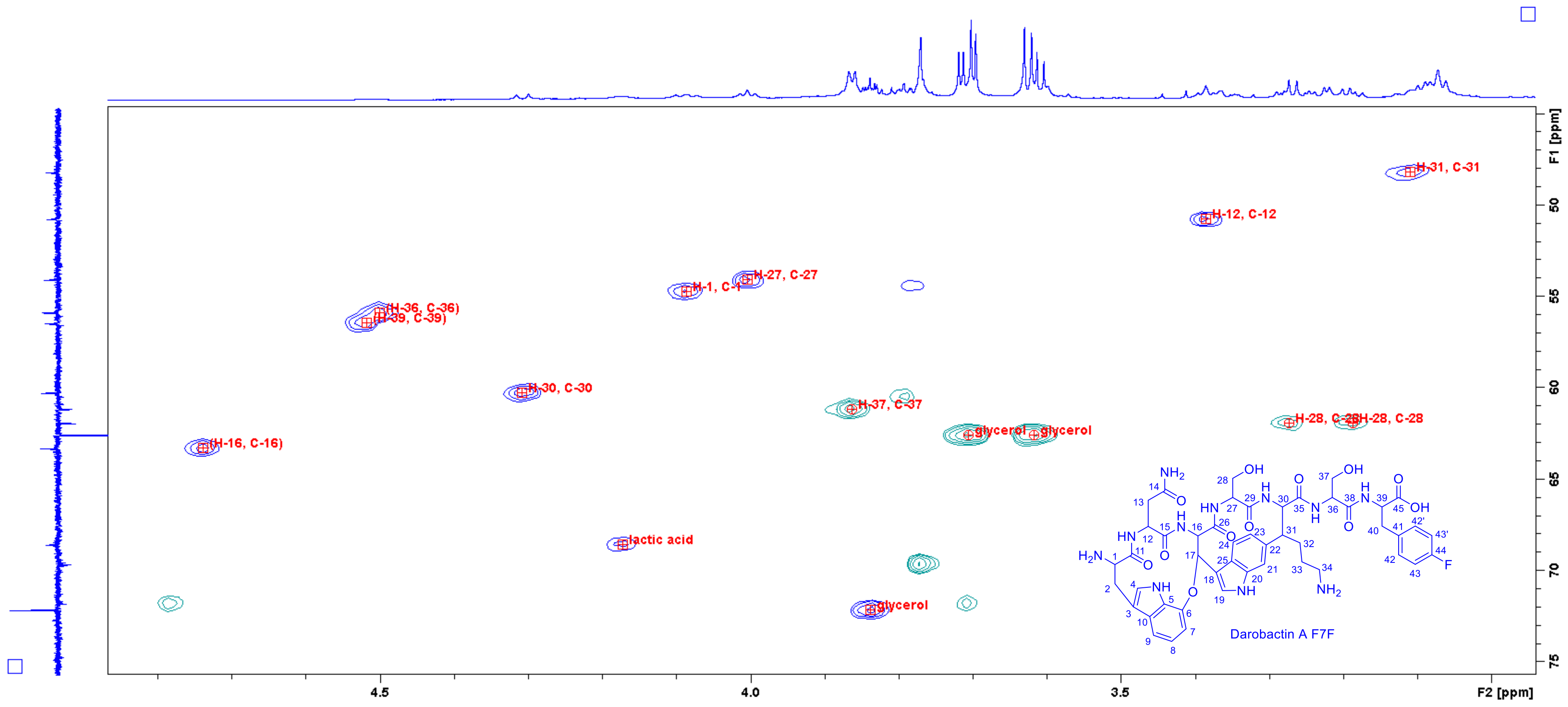

Figure S62: HSQC spectrum of darobactin A F7F (dilute sample, D_2O, ^1H: 700 MHz, ^{13}C: 176.1 MHz), measured with H_2O suppression. Close-up in the region of 4.8 – 3.0 ppm (F2 axis) and 75.0 – 45.0 ppm (F1 axis) with peak assignments. For easier peak identification the DEPTQ-135 experiment is shown on the F1 axis.

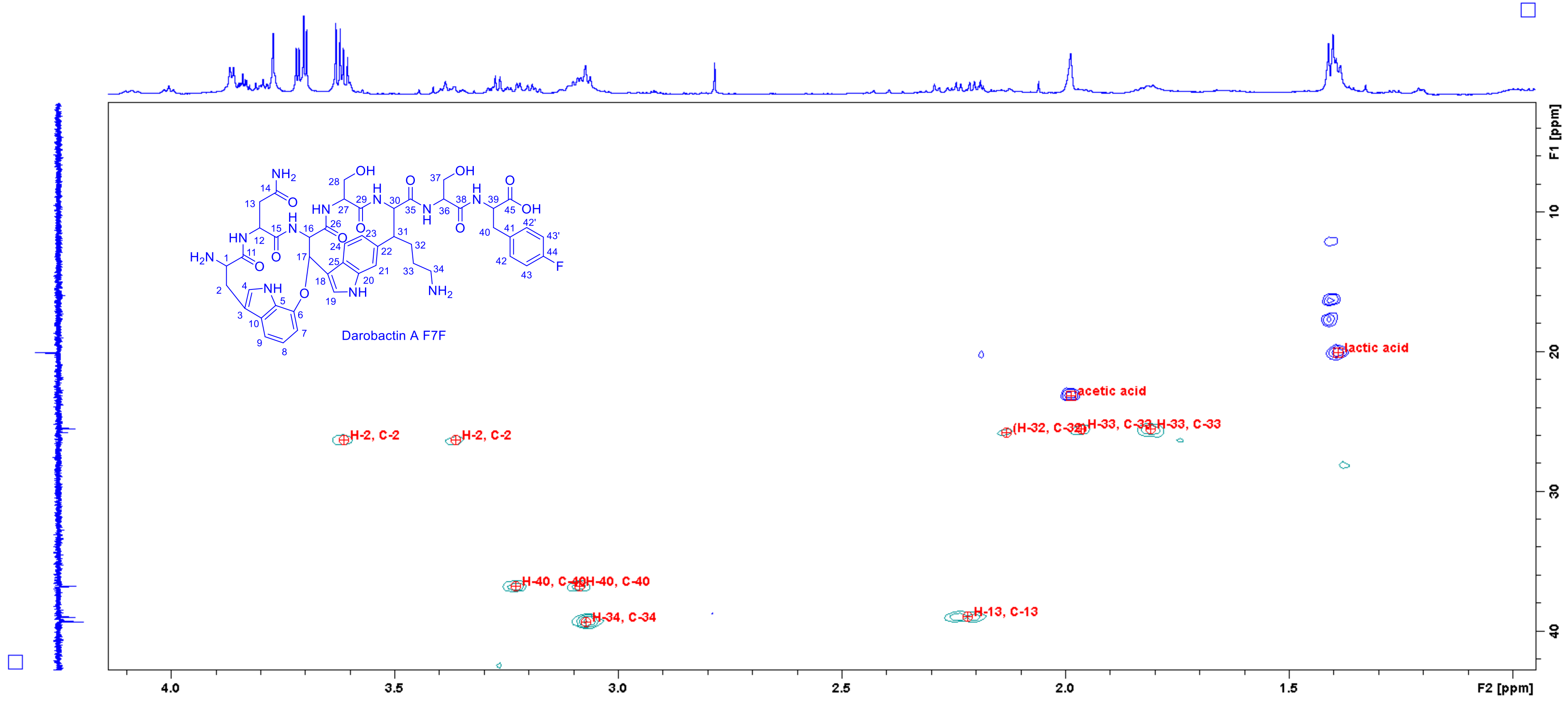

Figure S63: HSQC spectrum of darobactin A F7F (dilute sample, D_2O, ^1H: 700 MHz, ^{13}C: 176.1 MHz), measured with H_2O suppression. Close-up in the region of 4.1 – 1.0 ppm (F2 axis) and 42.0 – 4.0 ppm (F1 axis) with peak assignments. For easier peak identification the DEPTQ-135 experiment is shown on the F1 axis.

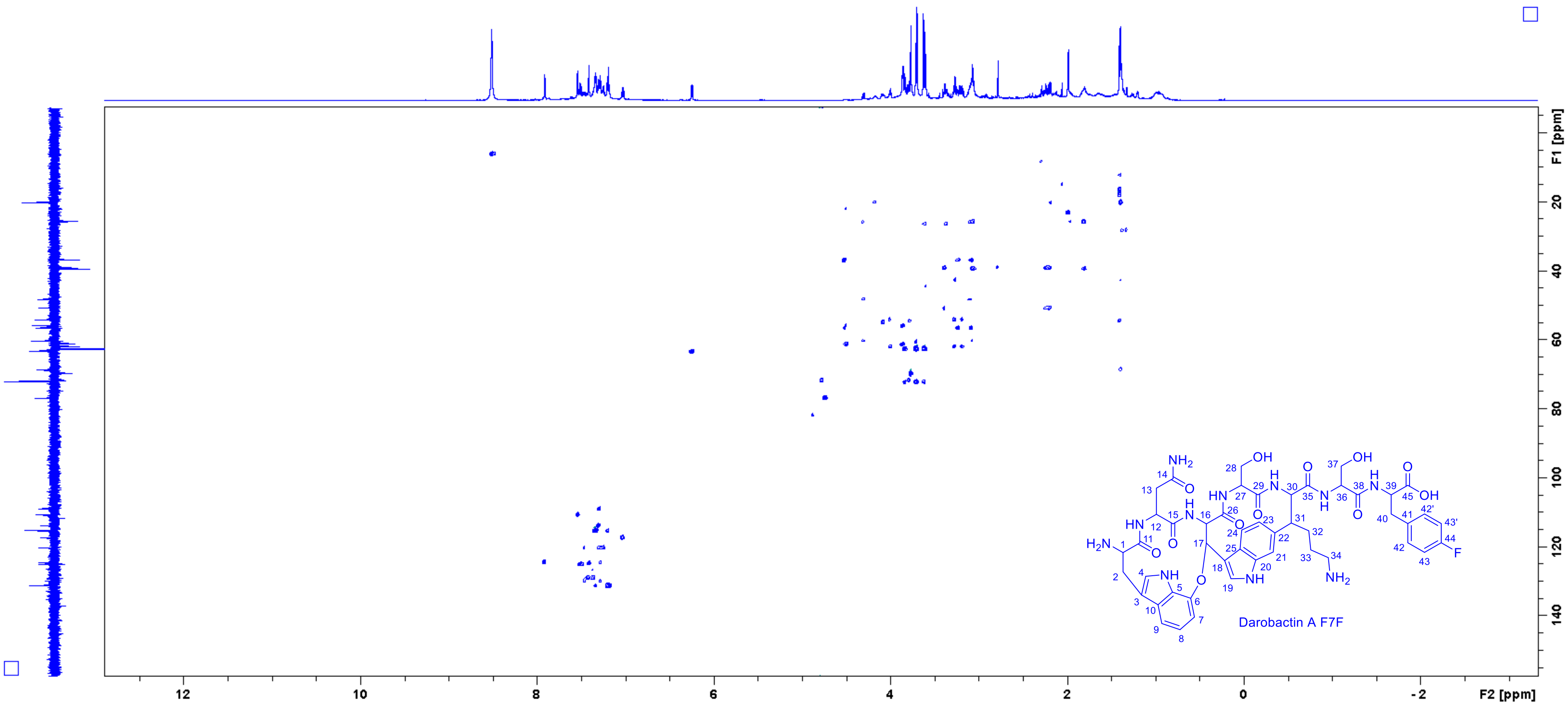

Figure S64: HSQC-TOCSY spectrum of darobactin A F7F (D_2O, ^1H: 700 MHz, ^{13}C: 176.1 MHz), measured with H_2O suppression. For easier peak identification the DEPTQ-135 experiment is shown on the F1 axis.

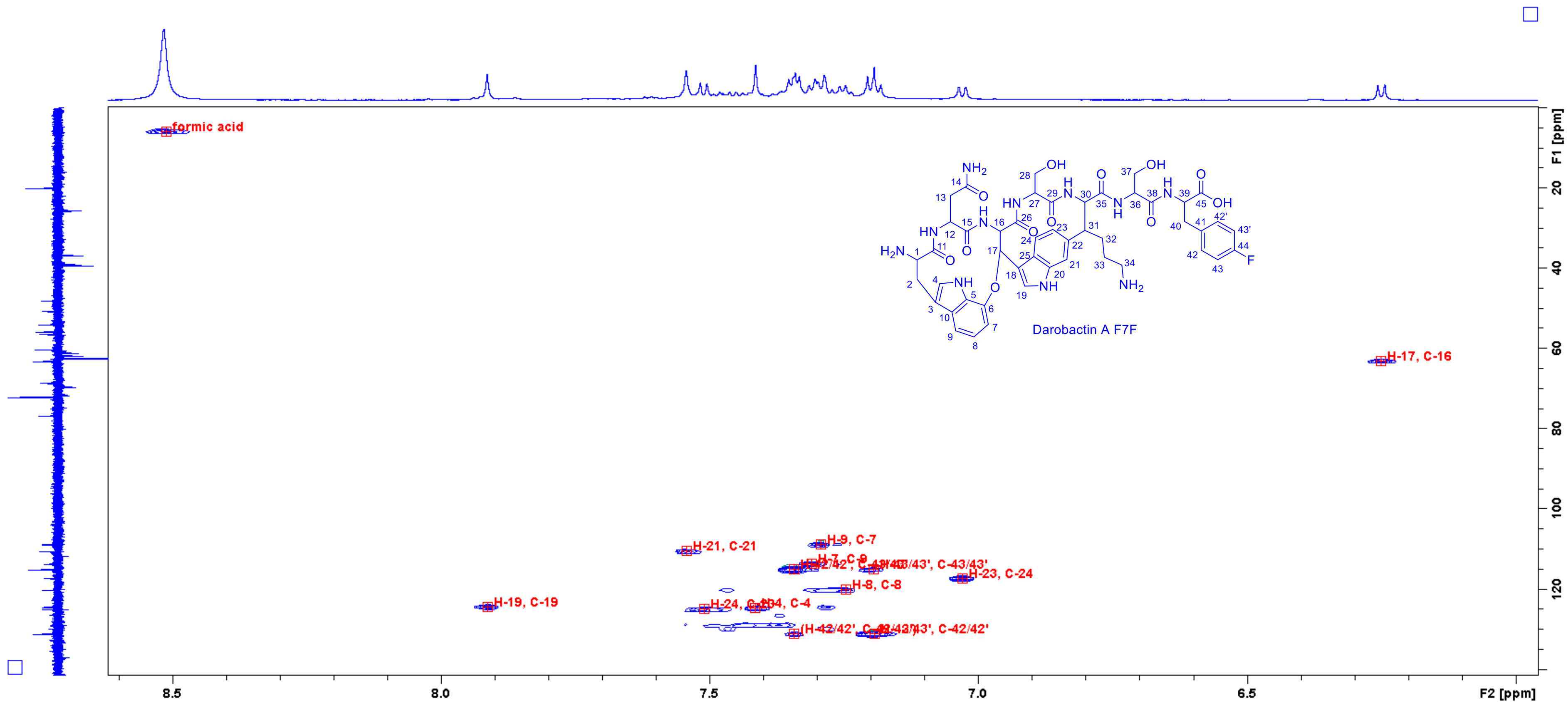

Figure S65: HSQC-TOCSY spectrum of darobactin A F7F (D_2O, ^{1}H: 700 MHz, ^{13}C: 176.1 MHz), measured with H_2O suppression. Close-up in the region of 8.6 – 6.0 ppm (F2 axis) and 140.0 – 0.0 ppm (F1 axis) with peak assignments. For easier peak identification the DEPTQ-135 experiment is shown on the F1 axis.

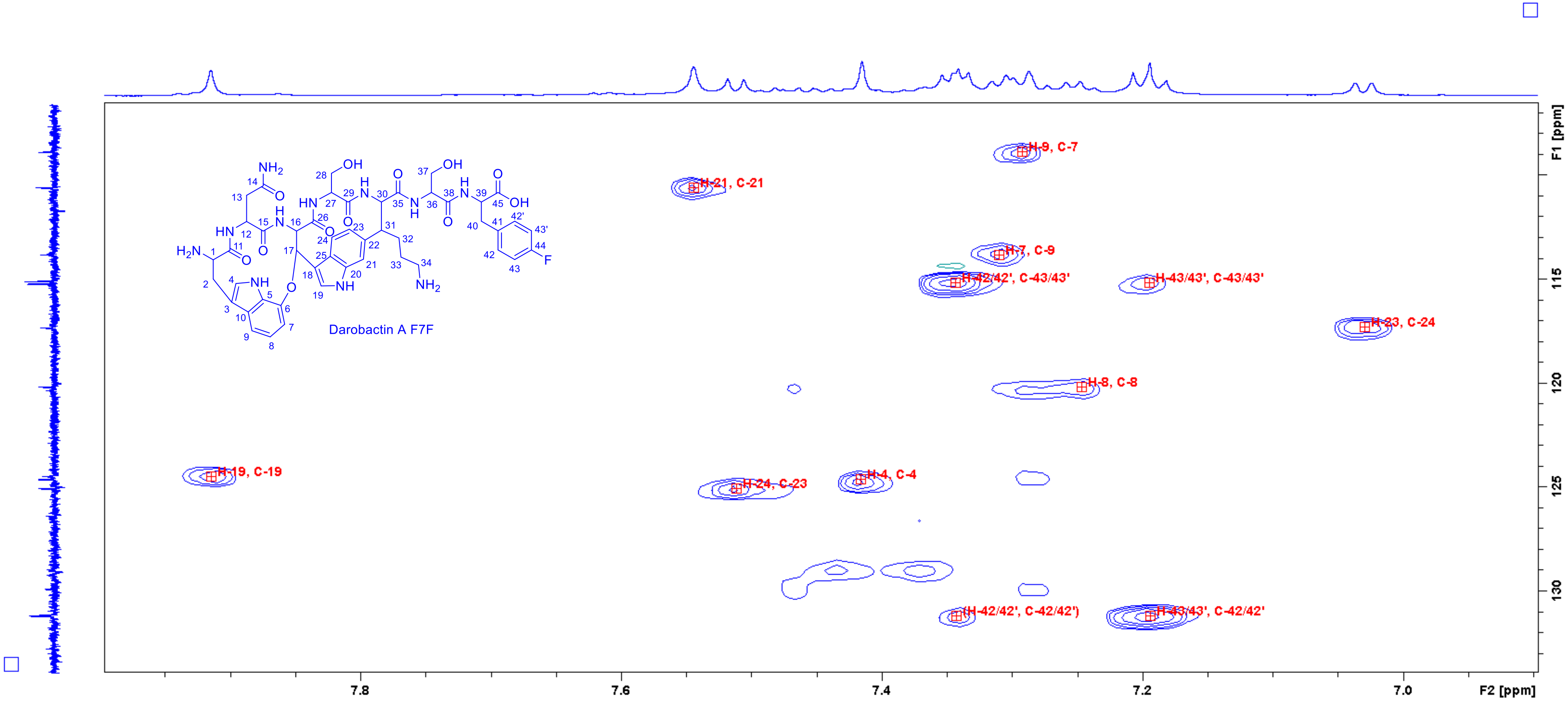

Figure S66: HSQC-TOCSY spectrum of darobactin A F7F (D_2O, ^{1}H: 700 MHz, ^{13}C: 176.1 MHz), measured with H_2O suppression. Close-up in the region of 7.95 – 6.90 ppm (F2 axis) and 133.0 – 107.0 ppm (F1 axis) with peak assignments. For easier peak identification the DEPTQ-135 experiment is shown on the F1 axis.

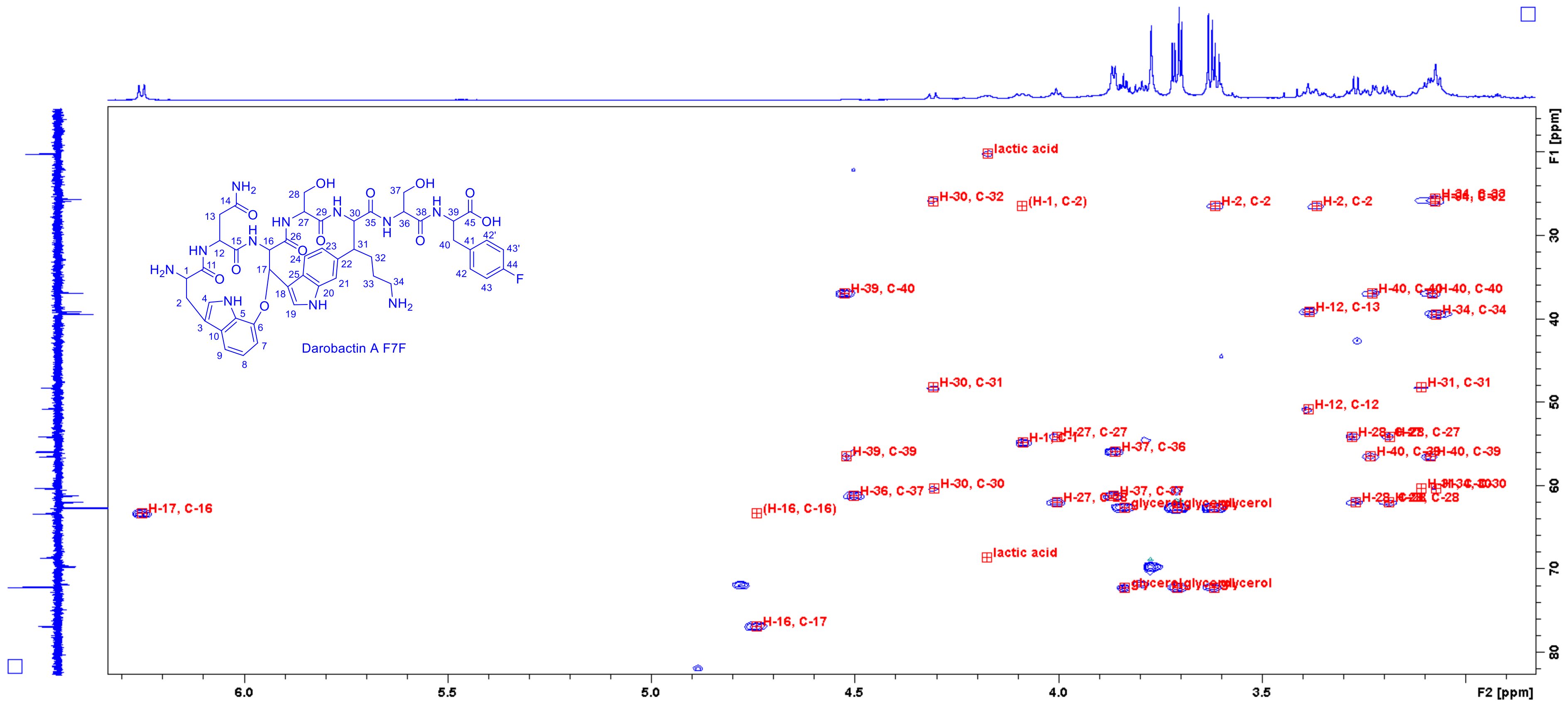

Figure S67: HSQC-TOCSY spectrum of darobactin A F7F (D_2O, ^1H: 700 MHz, ^{13}C: 176.1 MHz), measured with H_2O suppression. Close-up in the region of 6.3 – 2.9 ppm (F2 axis) and 82.0 – 16.0 ppm (F1 axis) with peak assignments. For easier peak identification the DEPTQ-135 experiment is shown on the F1 axis.

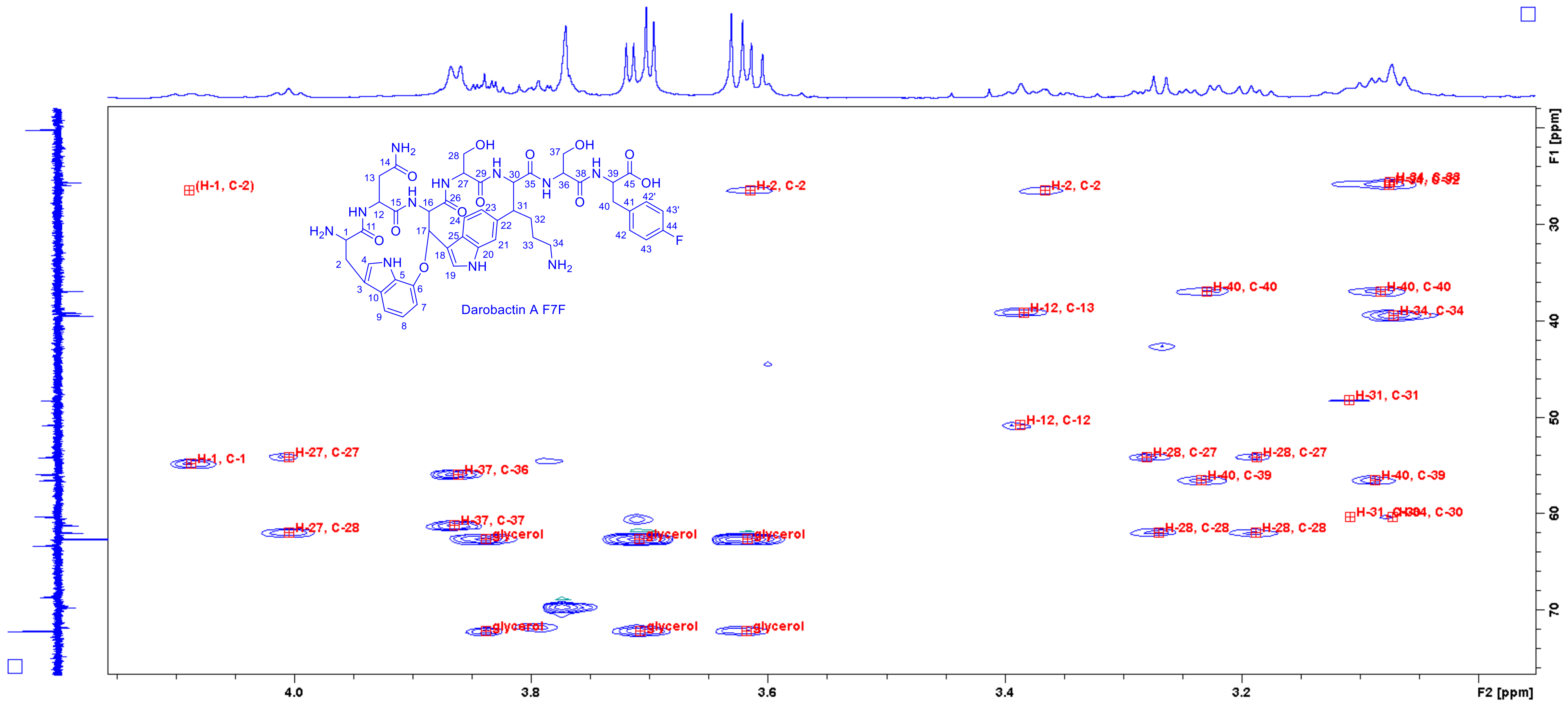

Figure S68: HSQC-TOCSY spectrum of darobactin A F7F (D_2O, ^1H: 700 MHz, ^{13}C: 176.1 MHz), measured with H_2O suppression. Close-up in the region of 4.15 – 3.00 ppm (F2 axis) and 76.0 – 18.0 ppm (F1 axis) with peak assignments. For easier peak identification the DEPTQ-135 experiment is shown on the F1 axis.

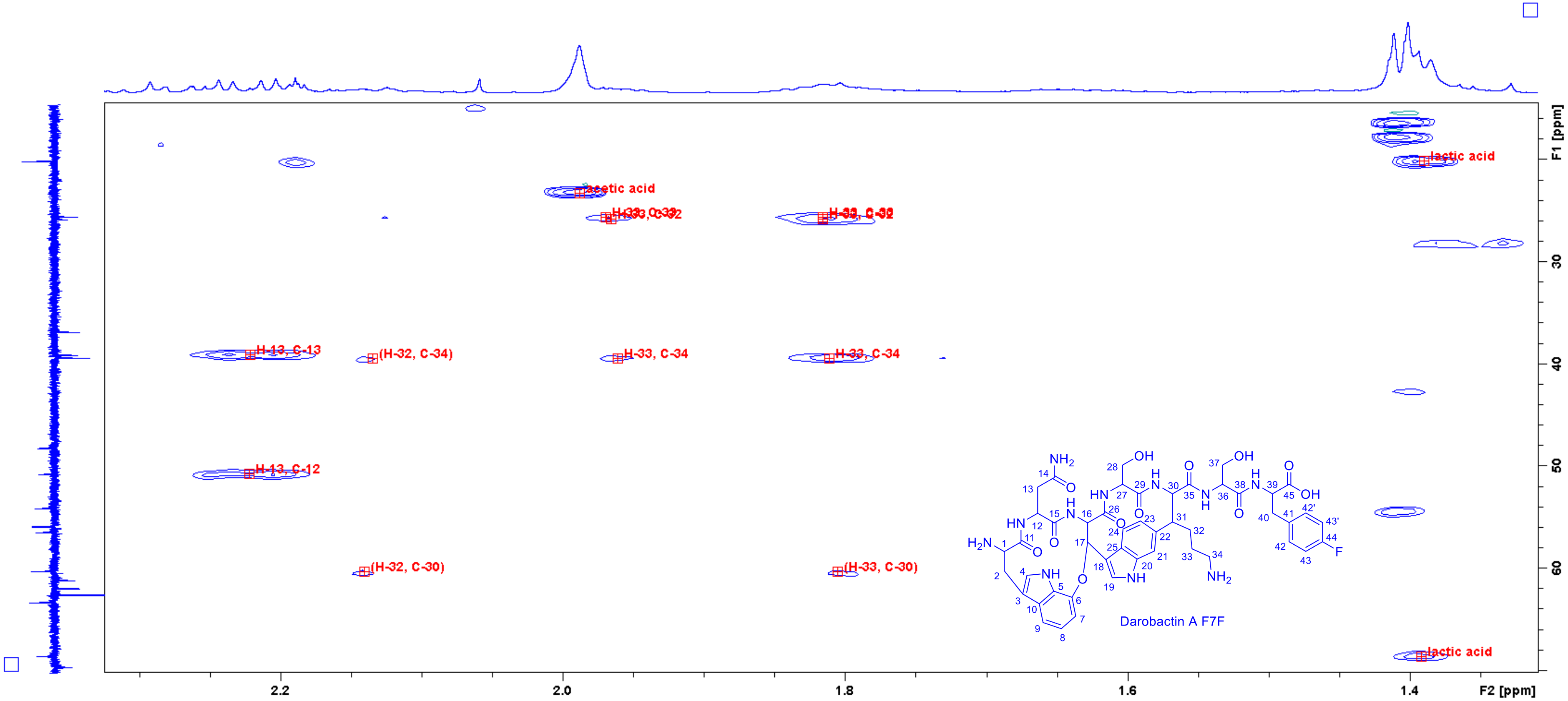

Figure S69: HSQC-TOCSY spectrum of darobactin A F7F (D_2O, ^1H: 700 MHz, ^{13}C: 176.1 MHz), measured with H_2O suppression. Close-up in the region of 2.30 – 1.35 ppm (F2 axis) and 70.0 – 16.0 ppm (F1 axis) with peak assignments. For easier peak identification the DEPTQ-135 experiment is shown on the F1 axis.

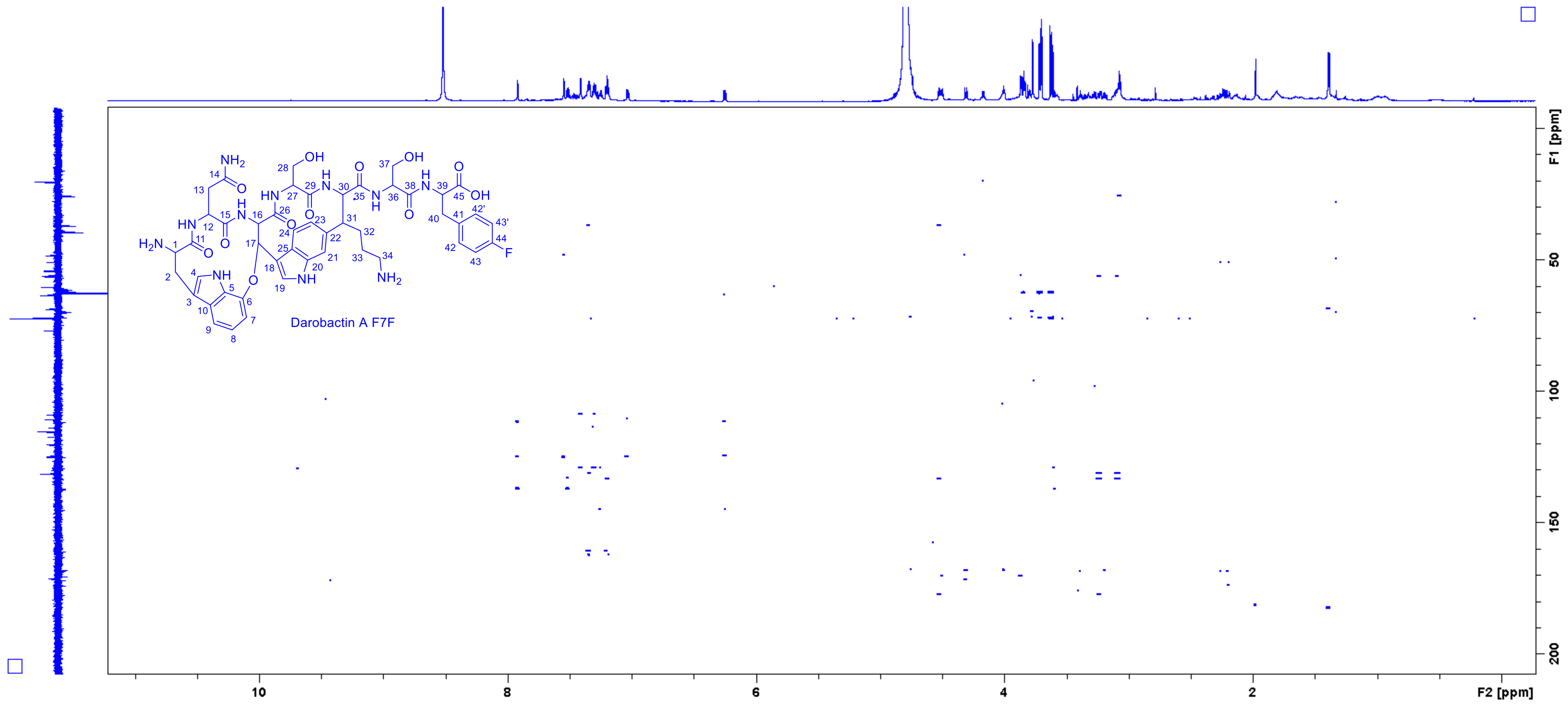

Figure S70: HMBC spectrum of darobactin A F7F (D_2O, ^1H: 700 MHz, ^{13}C: 176.1 MHz), measured with non-uniform sampling. For easier peak identification the DEPTQ-135 experiment is shown on the F1 axis.

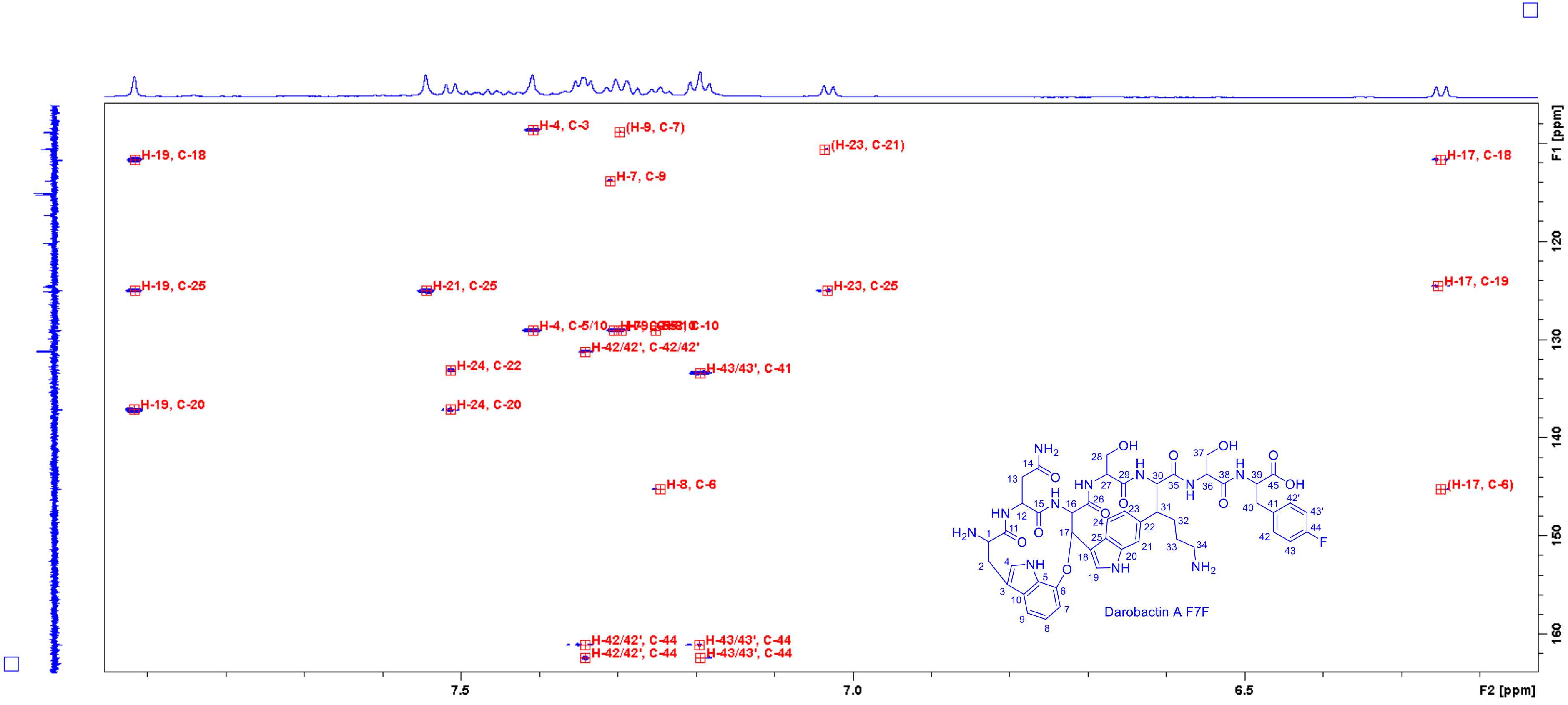

Figure S71: HMBC spectrum of darobactin A F7F (D_2O, ^1H: 700 MHz, ^{13}C: 176.1 MHz), measured with non-uniform sampling. Close-up in the region of 7.9 – 6.2 ppm (F2 axis) and 162.0 – 108.0 ppm (F1 axis) with peak assignments. For easier peak identification the DEPTQ-135 experiment is shown on the F1 axis.

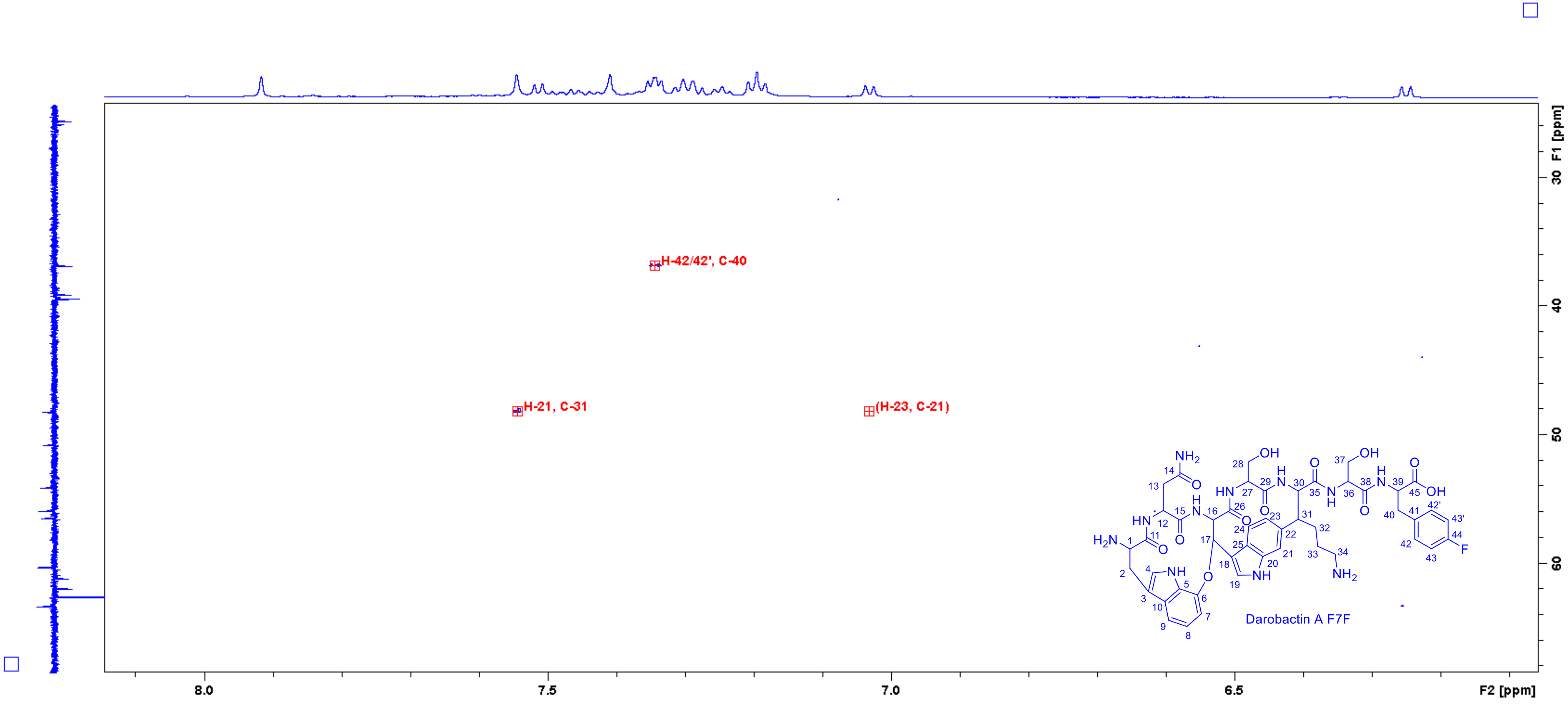

Figure S72: HMBC spectrum of darobactin A F7F (D_2O, ^1H: 700 MHz, ^{13}C: 176.1 MHz), measured with non-uniform sampling. Close-up in the region of 8.1 – 6.1 ppm (F2 axis) and 68.0 – 26.0 ppm (F1 axis) with peak assignments. For easier peak identification the DEPTQ-135 experiment is shown on the F1 axis.

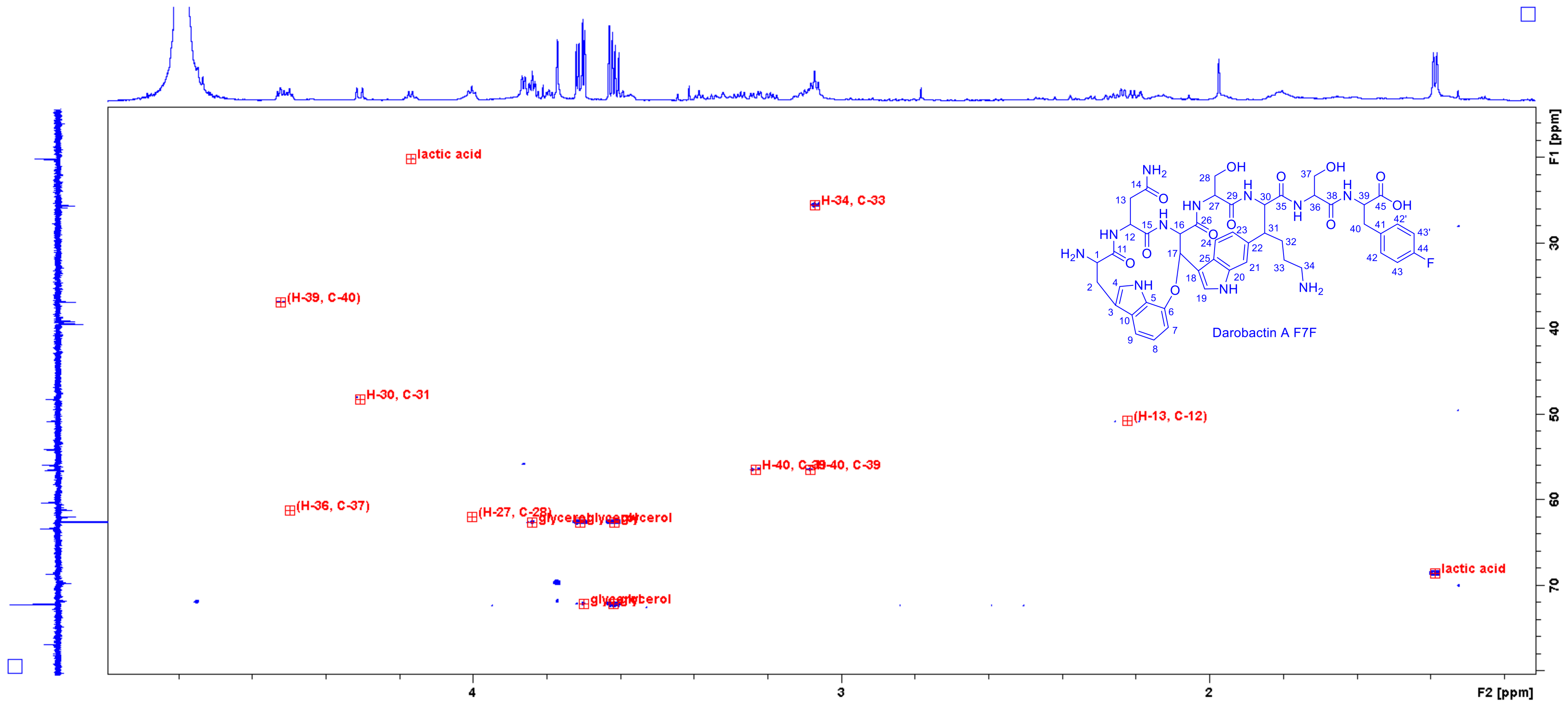

Figure S73: HMBC spectrum of darobactin A F7F (D_2O, ^{1}H: 700 MHz, ^{13}C: 176.1 MHz), measured with non-uniform sampling. Close-up in the region of 4.8 – 1.2 ppm (F2 axis) and 80.0 – 16.0 ppm (F1 axis) with peak assignments. For easier peak identification the DEPTQ-135 experiment is shown on the F1 axis.

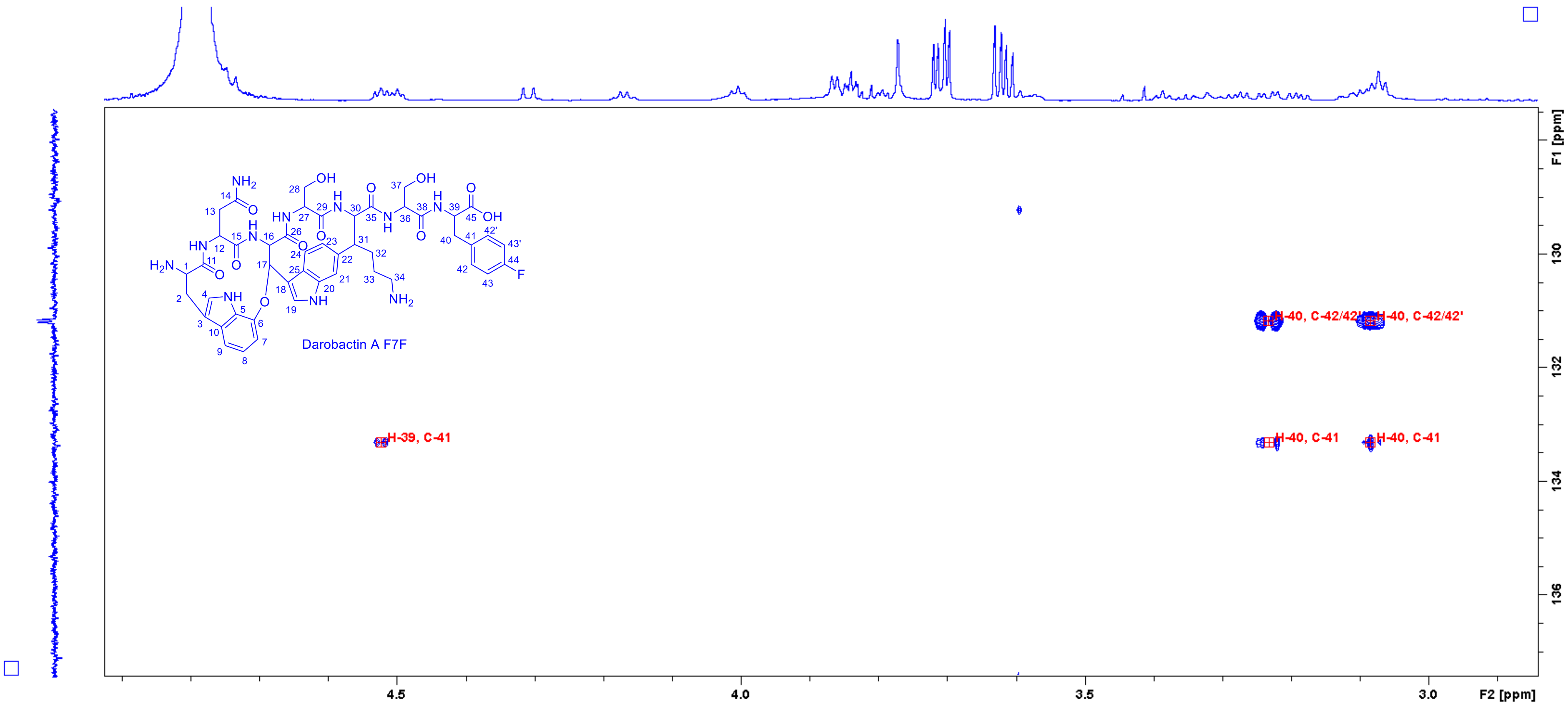

Figure S74: HMBC spectrum of darobactin A F7F (D_2O, ^1H: 700 MHz, ^{13}C: 176.1 MHz), measured with non-uniform sampling. Close-up in the region of 4.9 – 2.9 ppm (F2 axis) and 137.0 – 127.5 ppm (F1 axis) with peak assignments. For easier peak identification the DEPTQ-135 experiment is shown on the F1 axis.

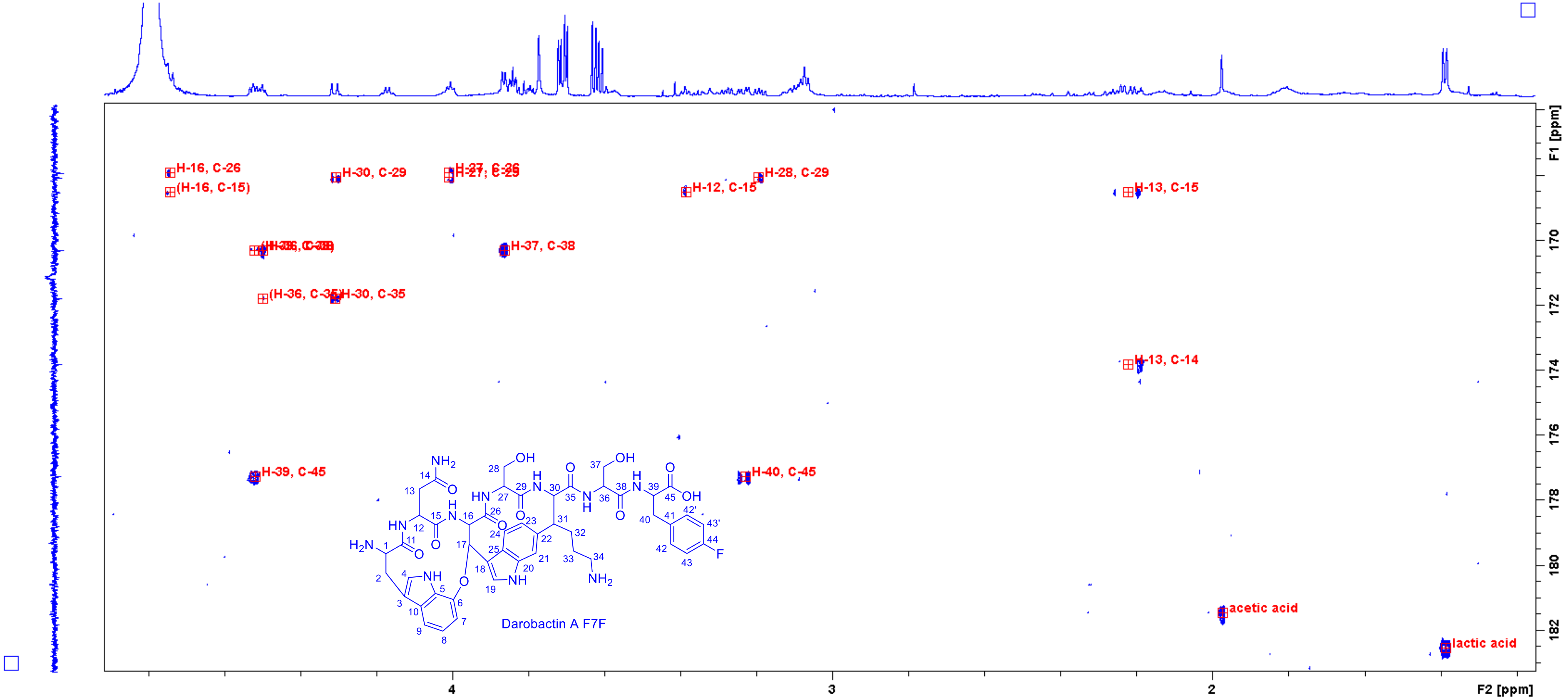

Figure S75: HMBC spectrum of darobactin A F7F (D_2O, ^{1}H: 700 MHz, ^{13}C: 176.1 MHz), measured with non-uniform sampling. Close-up in the region of 4.8 – 1.2 ppm (F2 axis) and 183.0 – 166.0 ppm (F1 axis) with peak assignments. For easier peak identification the DEPTQ-135 experiment is shown on the F1 axis.

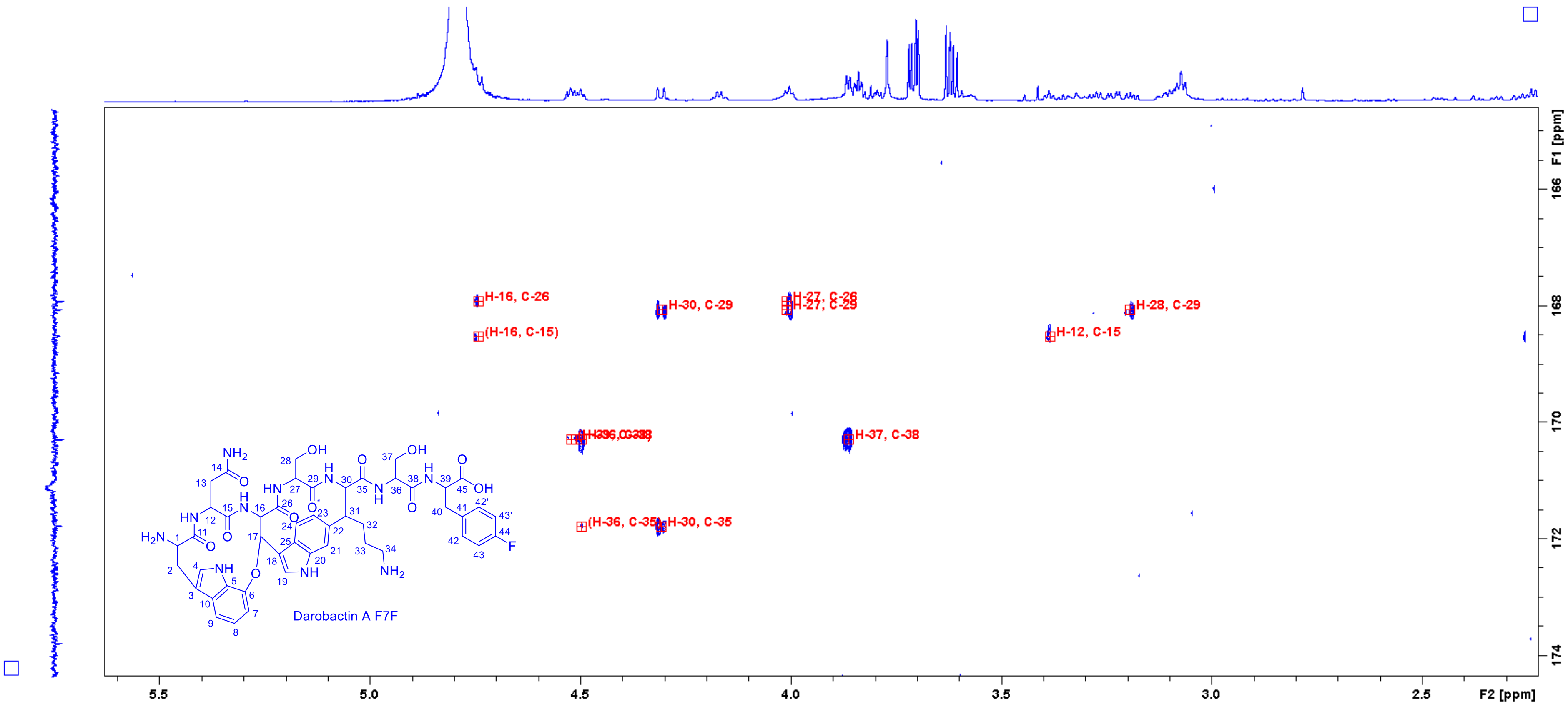

Figure S76: HMBC spectrum of darobactin A F7F (D_2O, ^1H: 700 MHz, ^{13}C: 176.1 MHz), measured with non-uniform sampling. Close-up in the region of 5.6 – 2.3 ppm (F2 axis) and 174.0 – 165.0 ppm (F1 axis) with peak assignments. For easier peak identification the DEPTQ-135 experiment is shown on the F1 axis.

Plasmid sequences

Sequence S1: Sequence of the plasmid pJK63.

5’-
GGGAATTGTGAGCGGATAACAATTCCCCTGTAGAAATAATTTTGTTTAACTTTAATAAGGAGATATACCCCGTG
TGCTTCTCAAATGCCTGAGGCCAGTTTGCTCAGGCTCTCCCCGTGGAGGTAATAATTGACGATATGATCAGTGC
ACGGCTAACTAAGCGGCCTGCTGACTTTCTCGCCGATCAAAAGGCATTTTGCTATTAAGGGATTGACGAGGGC
GTATCTGCGCAGTAAGATGCGCCCCGCATTGGAAACCTGATCATGTAGATCGAATGGACTCTAAATCCGTTCA
GCCGGGTTAGATTCCCGGGGTTTCCGCCAAATTCGAAAAGCCTGCTCAACGAGCAGGCTTTTTTGCATGTGCTT
AAGTCGAACAGAAAGTAATCGTATTGTACACGGCCGCATAATCGAAATTAATACGACTCACTATAGGGGAATT
GTGAGCGGATAACAATTCCCCATCTTAGTATATTAGTTAAGTATAAGAAGGAGATATACAATGCATAATACCTT
AAATGAAACCGTTAAAACTCAAGAAGCACTCAATTCTCTTGCTGCATCATTCAAAGAGACTGAACTCTCAATTA
CTGATAAAGCACTAAACGAATTAAGCAATAAACCTAAGATCCCTGAGATCACGGCCTGGAACTGGTCAAAAAG
CTTCCAGGAAATTTAAAGCTTATCCCATCAGGTTATTTTATTTTCCTGAAAAAACAATGTCCATTGGGATAAACA
ATCAAGTTGTTCAGTTAAAATAAATAATTTTTTGAACCTTAATCATAGGGTAATCCTATGTATTATCCGGGATTA
CCCTAAATACTTTCATAAAGTAAATATAAAAATTTGAATAATACAGATAAGTTTTTTAACCTGAATGGAAGGTT
TGTATTTTATCATTAAACACAACCACCTTTTACCACTAATATTATTGGATTTTTTCATTTCAAGATGAAAATATAA
TAGATTTCAGTAACTAAAAAGGGTAAAAATACGATAAAAAATCAATATTGGCAGAAAAAAACATTTTCGTTGG
ATCTTCATATATGTTATGTAGAATACTCAGAGGCAAGGGTTAAAAAATCCATACCCAAAAATTAATTTAAAAAT
TCGCCCCGCTAATAGCCAGCCTGTCCCAGCAGGTGTTACTGATGCAGGCAAATAAAATAGCCCTAATAGGATG
GGCTTTAAGTAAGGAACGGTAGCCATAAATGGACACAATAATCCCCATAAAATATTTAGATTCAGACGAATCA
TCGATTCTTAAGAAATCATCTAAAATTAACTACAGGCAATTAGCTTGCAGAATTATCGGTGAAATCTCCGCCGA
AAAGATATTAGATGATGATGAACTGGCTTTATATAATAAAGAAATCAGTATACATTTCAGCCCTGAAATTATTA
ATGCTAATAAATTAGTTGTGGTTGTGAAAGCCACCAGGCTTTGCAATTTAAGATGCACTTATTGTCACTCCTGG
GCAGAAGGAAAAGGAAATACCTTAACATTCTTCAATTTAATGCGTTCCATCCACCGTTTCTTATCCCTACCGAAT
ATCAAGCGATTTGAATTCGTCTGGCATGGTGGCGAAGTAACGTTGTTGAATGTTAATTACTTTAAGAAACTCAT
CTGGTTACAGGAACAATTTAAAAAACCGGATCAAGTTATCACCAATTCGGTACAGACAAATGCCGTCAATATTC
CTGAAGATTGGTTAGTGTTCCTCAAAGGTATTGGAATGGGGGTAGGAATAAGCGTTGATGGTATTCCGGAAAT
ACACGATAGCAGGAGATTAGATTACAGAGGAAGGCCAACATCCCATAAAGTCGCGGCAGGTATGAAAAAGTT
AAGAAGTTATGGCATACCTTACGGTGCGCTTATCGTCGTCGACCGCGATGTTTATGAATCAAATATAGAAAAA
ATGCTCTCTTATTTTTACGAAATCGGTTTGATGGATATTGAATTTCTGAATATTGTCCCAGATAACCGATGCCAG
CCGGGTGATGATCCTGGAGGAAGTTATATAACTTACCATAACTATATTAATTTCCTTTCTAAGGTTTTCCGTGTC
TGGTGGAATGGTTATCAAGGCAAAATCAATATTCGCTTGTTTGACGGATTTATTGACAGTATCAAATCGTCCCA
AAAGAAAATGTCAGATTGTTATTGGGCGGGTAACTGTTCTCAGGAAATAATCACATTAGAACCTAATGGTACG
GTATCAGCATGTGATAAATATGTTGGTGCTGAAGGGAATAATTATGGTTCGATTATTGATAATGATCTTGGGA
ATTTACTATCTAAATCAAATACAAATAAGGATCATCTTAAAGAGGAAATGGAATCATATGAAAAAATGCATCAA
TGTAAATGGTTTCATTTGTGTAATGGTGGATGCCCACACGATCGAGTGACCAACAGGAAGCACAATCCAAATT
ATGATGGTTCATGTTGTGGAACCGGCGGTTTGTTGGAGACAATAAAACAAACCATCGCGGCGTAAGCTGCTGC
CACCGCTGAGCAATAACTAGCATAACCCCTTGGGGCCTCTAAACGGGTCTTGAGGGGTTTTTTGCTGAAACCTC
AGGCATTTGAGAAGCACACGGTCACACTGCTTCCGGTAGTCAATAAACCGGTAAACCAGCAATAGACATAAGC
GGCTATTTAACGACCCTGCCCTGAACCGACGACAAGCTGACGACCGGGTCTCCGCAAGTGGCACTTTTCGGGG
AAATGTGCGCGGAACCCCTATTTGTTTATTTTTCTAAATACATTCAAATATGTATCCGCTCATGAATTAATTCTTA
GAAAAACTCATCGAGCATCAAATGAAACTGCAATTTATTCATATCAGGATTATCAATACCATATTTTTGAAAAA
GCCGTTTCTGTAATGAAGGAGAAAACTCACCGAGGCAGTTCCATAGGATGGCAAGATCCTGGTATCGGTCTGC
GATTCCGACTCGTCCAACATCAATACAACCTATTAATTTCCCCTCGTCAAAAATAAGGTTATCAAGTGAGAAATC
ACCATGAGTGACGACTGAATCCGGTGAGAATGGCAAAAGTTTATGCATTTCTTTCCAGACTTGTTCAACAGGCC

AGCCATTACGCTCGTCATCAAAATCACTCGCATCAACCAAACCGTTATTCATTCGTGATTGCGCCTGAGCGAGA
CGAAATACGCGGTCGCTGTTAAAAGGACAATTACAAACAGGAATCGAATGCAACCGGCGCAGGAACACTGCC
AGCGCATCAACAATATTTTCACCTGAATCAGGATATTCTTCTAATACCTGGAATGCTGTTTTCCCGGGGATCGCA
GTGGTGAGTAACCATGCATCATCAGGAGTACGGATAAAATGCTTGATGGTCGGAAGAGGCATAAATTCCGTC
AGCCAGTTTAGTCTGACCATCTCATCTGTAACATCATTGGCAACGCTACCTTTGCCATGTTTCAGAAACAACTCT
GGCGCATCGGGCTTCCCATACAATCGATAGATTGTCGCACCTGATTGCCCGACATTATCGCGAGCCCATTTATA
CCCATATAAATCAGCATCCATGTTGGAATTTAATCGCGGCCTAGAGCAAGACGTTTCCCGTTGAATATGGCTCA
TACTCTTCCTTTTTCAATATTATTGAAGCATTTATCAGGGTTATTGTCTCATGAGCGGATACATATTTGAATGTAT
TTAGAAAAATAAACAAATAGGCATGCAGCGCTCTTCCGCTTCCTCGCTCACTGACTCGCTACGCTCGGTCGTTC
GACTGCGGCGAGCGGTGTCAGCTCACTCAAAAGCGGTAATACGGTTATCCACAGAATCAGGGGATAAAGCCG
GAAAGAACATGTGAGCAAAAAGCAAAGCACCGGAAGAAGCCAACGCCGCAGGCGTTTTTCCATAGGCTCCGC
CCCCCTGACGAGCATCACAAAAATCGACGCTCAAGCCAGAGGTGGCGAAACCCGACAGGACTATAAAGATAC
CAGGCGTTTCCCCCTGGAAGCTCCCTCGTGCGCTCTCCTGTTCCGACCCTGCCGCTTACCGGATACCTGTCCGCC
TTTCTCCCTTCGGGAAGCGTGGCGCTTTCTCATAGCTCACGCTGTTGGTATCTCAGTTCGGTGTAGGTCGTTCGC
TCCAAGCTGGGCTGTGTGCACGAACCCCCCGTTCAGCCCGACCGCTGCGCCTTATCCGGTAACTATCGTCTTGA
GTCCAACCCGGTAAGACACGACTTATCGCCACTGGCAGCAGCCATTGGTAACTGATTTAGAGGACTTTGTCTTG
AAGTTATGCACCTGTTAAGGCTAAACTGAAAGAACAGATTTTGGTGAGTGCGGTCCTCCAACCCACTTACCTTG
GTTCAAAGAGTTGGTAGCTCAGCGAACCTTGAGAAAACCACCGTTGGTAGCGGTGGTTTTTCTTTATTTATGAG
ATGATGAATCAATCGGTCTATCAAGTCAACGAACAGCTATTCCGTTACTCTAGATTTCAGTGCAATTTATCTCTT
CAAATGTAGCACCTGAAGTCAGCCCCATACGATATAAGTTGTAATTCTCATGTTAGTCATGCCCCGCGCCCACC
GGAAGGAGCTGACTGGGTTGAAGGCTCTCAAGGGCATCGGTCGAGATCCCGGTGCCTAATGAGTGAGCTAAC
TTACATTAATTGCGTTGCGCTCACTGCCCGCTTTCCAGTCGGGAAACCTGTCGTGCCAGCTGCATTAATGAATC
GGCCAACGCGCGGGGAGAGGCGGTTTGCGTATTGGGCGCCAGGGTGGTTTTTCTTTTCACCAGTGAGACGGG
CAACAGCTGATTGCCCTTCACCGCCTGGCCCTGAGAGAGTTGCAGCAAGCGGTCCACGCTGGTTTGCCCCAGC
AGGCGAAAATCCTGTTTGATGGTGGTTAACGGCGGGATATAACATGAGCTGTCTTCGGTATCGTCGTATCCCA
CTACCGAGATGTCCGCACCAACGCGCAGCCCGGACTCGGTAATGGCGCGCATTGCGCCCAGCGCCATCTGATC
GTTGGCAACCAGCATCGCAGTGGGAACGATGCCCTCATTCAGCATTTGCATGGTTTGTTGAAAACCGGACATG
GCACTCCAGTCGCCTTCCCGTTCCGCTATCGGCTGAATTTGATTGCGAGTGAGATATTTATGCCAGCCAGCCAG
ACGCAGACGCGCCGAGACAGAACTTAATGGGCCCGCTAACAGCGCGATTTGCTGGTGACCCAATGCGACCAG
ATGCTCCACGCCCAGTCGCGTACCGTCTTCATGGGAGAAAATAATACTGTTGATGGGTGTCTGGTCAGAGACA
TCAAGAAATAACGCCGGAACATTAGTGCAGGCAGCTTCCACAGCAATGGCATCCTGGTCATCCAGCGGATAGT
TAATGATCAGCCCACTGACGCGTTGCGCGAGAAGATTGTGCACCGCCGCTTTACAGGCTTCGACGCCGCTTCG
TTCTACCATCGACACCACCACGCTGGCACCCAGTTGATCGGCGCGAGATTTAATCGCCGCGACAATTTGCGACG
GCGCGTGCAGGGCCAGACTGGAGGTGGCAACGCCAATCAGCAACGACTGTTTGCCCGCCAGTTGTTGTGCCA
CGCGGTTGGGAATGTAATTCAGCTCCGCCATCGCCGCTTCCACTTTTTCCCGCGTTTTCGCAGAAACGTGGCTG
GCCTGGTTCACCACGCGGGAAACGGTCTGATAAGAGACACCGGCATACTCTGCGACATCGTATAACGTTACTG
GTTTCACATTCACCACCCTGAATTGACTCTCTTCCGGGCGCTATCATGCCATACCGCGAAAGGTTTTGCGCCATT
CGATGGTGTCCGGGATCTCGACGCTCTCCCTTATGCGACTCCTGCATTAGGAAATTAATACGACTCACTATAG-
3’

Sequence S2: Sequence of the plasmid pJK64.

5'-
TCTCATGAGCGGATACATATTTGAATGTATTTAGAAAAATAAACAAATAGGCATGCAGCGCTCTTCCGCTTCCT
CGCTCACTGACTCGCTACGCTCGGTCGTTCGACTGCGGCGAGCGGTGTCAGCTCACTCAAAAGCGGTAATACG
GTTATCCACAGAATCAGGGGATAAAGCCGGAAAGAACATGTGAGCAAAAAGCAAAGCACCGGAAGAAGCCA
ACGCCGCAGGCGTTTTTCCATAGGCTCCGCCCCCCTGACGAGCATCACAAAAATCGACGCTCAAGCCAGAGGT
GGCGAAACCCGACAGGACTATAAAGATACCAGGCGTTTCCCCCTGGAAGCTCCCTCGTGCGCTCTCCTGTTCCG
ACCCTGCCGCTTACCGGATACCTGTCCGCCTTTCTCCCTTCGGGAAGCGTGGCGCTTTCTCATAGCTCACGCTGT
TGGTATCTCAGTTCGGTGTAGGTCGTTCGCTCCAAGCTGGGCTGTGTGCACGAACCCCCCGTTCAGCCCGACCG
CTGCGCCTTATCCGGTAACTATCGTCTTGAGTCCAACCCGGTAAGACACGACTTATCGCCACTGGCAGCAGCCA
TTGGTAACTGATTTAGAGGACTTTGTCTTGAAGTTATGCACCTGTTAAGGCTAAACTGAAAGAACAGATTTTGG
TGAGTGCGGTCCTCCAACCCACTTACCTTGGTTCAAAGAGTTGGTAGCTCAGCGAACCTTGAGAAAACCACCGT
TGGTAGCGGTGGTTTTTCTTTATTTATGAGATGATGAATCAATCGGTCTATCAAGTCAACGAACAGCTATTCCG
TTACTCTAGATTTCAGTGCAATTTATCTCTTCAAATGTAGCACCTGAAGTCAGCCCCATACGATATAAGTTGTAA
TTCTCATGTTAGTCATGCCCCGCGCCCACCGGAAGGAGCTGACTGGGTTGAAGGCTCTCAAGGGCATCGGTCG
AGATCCCGGTGCCTAATGAGTGAGCTAACTTACATTAATTGCGTTGCGCTCACTGCCCGCTTTCCAGTCGGGAA
ACCTGTCGTGCCAGCTGCATTAATGAATCGGCCAACGCGCGGGGAGAGGCGGTTTGCGTATTGGGCGCCAGG
GTGGTTTTTCTTTTCACCAGTGAGACGGGCAACAGCTGATTGCCCTTCACCGCCTGGCCCTGAGAGAGTTGCAG
CAAGCGGTCCACGCTGGTTTGCCCCAGCAGGCGAAAATCCTGTTTGATGGTGGTTAACGGCGGGATATAACAT
GAGCTGTCTTCGGTATCGTCGTATCCCACTACCGAGATGTCCGCACCAACGCGCAGCCCGGACTCGGTAATGG
CGCGCATTGCGCCCAGCGCCATCTGATCGTTGGCAACCAGCATCGCAGTGGGAACGATGCCCTCATTCAGCAT
TTGCATGGTTTGTTGAAAACCGGACATGGCACTCCAGTCGCCTTCCCGTTCCGCTATCGGCTGAATTTGATTGC
GAGTGAGATATTTATGCCAGCCAGCCAGACGCAGACGCGCCGAGACAGAACTTAATGGGCCCGCTAACAGCG
CGATTTGCTGGTGACCCAATGCGACCAGATGCTCCACGCCCAGTCGCGTACCGTCTTCATGGGAGAAAATAAT
ACTGTTGATGGGTGTCTGGTCAGAGACATCAAGAAATAACGCCGGAACATTAGTGCAGGCAGCTTCCACAGCA
ATGGCATCCTGGTCATCCAGCGGATAGTTAATGATCAGCCCACTGACGCGTTGCGCGAGAAGATTGTGCACCG
CCGCTTTACAGGCTTCGACGCCGCTTCGTTCTACCATCGACACCACCACGCTGGCACCCAGTTGATCGGCGCGA
GATTTAATCGCCGCGACAATTTGCGACGGCGCGTGCAGGGCCAGACTGGAGGTGGCAACGCCAATCAGCAAC
GACTGTTTGCCCGCCAGTTGTTGTGCCACGCGGTTGGGAATGTAATTCAGCTCCGCCATCGCCGCTTCCACTTT
TTCCCGCGTTTTCGCAGAAACGTGGCTGGCCTGGTTCACCACGCGGGAAACGGTCTGATAAGAGACACCGGCA
TACTCTGCGACATCGTATAACGTTACTGGTTTCACATTCACCACCCTGAATTGACTCTCTTCCGGGCGCTATCAT
GCCATACCGCGAAAGGTTTTGCGCCATTCGATGGTGTCCGGGATCTCGACGCTCTCCCTTATGCGACTCCTGCA
TTAGGAAATTAATACGACTCACTATAGGGGAATTGTGAGCGGATAACAATTCCCCTGTAGAAATAATTTTGTTT
AACTTTAATAAGGAGATATACCCCGTGTGCTTCTCAAATGCCTGAGGCCAGTTTGCTCAGGCTCTCCCCGTGGA
GGTAATAATTGACGATATGATCAGTGCACGGCTAACTAAGCGGCCTGCTGACTTTCTCGCCGATCAAAAGGCA
TTTTGCTATTAAGGGATTGACGAGGGCGTATCTGCGCAGTAAGATGCGCCCCGCATTGGAAACCTGATCATGT
AGATCGAATGGACTCTAAATCCGTTCAGCCGGGTTAGATTCCCGGGGTTTCCGCCAAATTCGAAAAGCCTGCTC
AACGAGCAGGCTTTTTTGCATGTGCTTAAGTCGAACAGAAAGTAATCGTATTGTACACGGCCGCATAATCGAA
ATTAATACGACTCACTATAGGGGAATTGTGAGCGGATAACAATTCCCCATCTTAGTATATTAGTTAAGTATAAG
AAGGAGATATACAATGCATAATACCTTAAATGAAACCGTTAAAACTCAAGAAGCACTCAATTCTCTTGCTGCAT
CATTCAAAGAGACTGAACTCTCAATTACTGATAAAGCACTAAACGAATTAAGCAATAAACCTAAGATCCCTGAG
ATCACGGCCTGGAACTGGTCAAAAAGCTAGCAGGAAATTTAAAGCTTATCCCATCAGGTTATTTTATTTTCCTG
AAAAAACAATGTCCATTGGGATAAACAATCAAGTTGTTCAGTTAAAATAAATAATTTTTTGAACCTTAATCATA
GGGTAATCCTATGTATTATCCGGGATTACCCTAAATACTTTCATAAAGTAAATATAAAAATTTGAATAATACAG
ATAAGTTTTTTTAACCTGAATGGAAGGTTTGTATTTTATCATTAAACACAACCACCTTTTACCACTAATATTATTG
GATTTTTTCATTTCAAGATGAAAATATAATAGATTTCAGTAACTAAAAAGGGTAAAAATACGATAAAAAATCAA
TATTGGCAGAAAAAAACATTTTCGTTGGATCTTCATATATGTTATGTAGAATACTCAGAGGCAAGGGTTAAAAA
ATCCATACCCAAAAATTAATTTAAAAATTCGCCCCGCTAATAGCCAGCCTGTCCCAGCAGGTGTTACTGATGCA
GGCAAATAAAATAGCCCTAATAGGATGGGCTTTAAGTAAGGAACGGTAGCCATAAATGGACACAATAATCCCC

ATAAAATATTTAGATTCAGACGAATCATCGATTCTTAAGAAATCATCTAAAATTAACTACAGGCAATTAGCTTGC
AGAATTATCGGTGAAATCTCCGCCGAAAAGATATTAGATGATGATGAACTGGCTTTATATAATAAAGAAATCA
GTATACATTTCAGCCCTGAAATTATTAATGCTAATAAATTAGTTGTGGTTGTGAAAGCCACCAGGCTTTGCAATT
TAAGATGCACTTATTGTCACTCCTGGGCAGAAGGAAAAGGAAATACCTTAACATTCTTCAATTTAATGCGTTCC
ATCCACCGTTTCTTATCCCTACCGAATATCAAGCGATTTGAATTCGTCTGGCATGGTGGCGAAGTAACGTTGTT
GAATGTTAATTACTTTAAGAAACTCATCTGGTTACAGGAACAATTTAAAAAACCGGATCAAGTTATCACCAATT
CGGTACAGACAAATGCCGTCAATATTCCTGAAGATTGGTTAGTGTTCCTCAAAGGTATTGGAATGGGGGTAGG
AATAAGCGTTGATGGTATTCCGGAAATACACGATAGCAGGAGATTAGATTACAGAGGAAGGCCAACATCCCAT
AAAGTCGCGGCAGGTATGAAAAAGTTAAGAAGTTATGGCATACCTTACGGTGCGCTTATCGTCGTCGACCGCG
ATGTTTATGAATCAAATATAGAAAAAATGCTCTCTTATTTTTACGAAATCGGTTTGATGGATATTGAATTTCTGA
ATATTGTCCCAGATAACCGATGCCAGCCGGGTGATGATCCTGGAGGAAGTTATATAACTTACCATAACTATATT
AATTTCCTTTCTAAGGTTTTCCGTGTCTGGTGGAATGGTTATCAAGGCAAAATCAATATTCGCTTGTTTGACGGA
TTTATTGACAGTATCAAATCGTCCCAAAAGAAAATGTCAGATTGTTATTGGGCGGGTAACTGTTCTCAGGAAAT
AATCACATTAGAACCTAATGGTACGGTATCAGCATGTGATAAATATGTTGGTGCTGAAGGGAATAATTATGGT
TCGATTATTGATAATGATCTTGGGAATTTACTATCTAAATCAAATACAAATAAGGATCATCTTAAAGAGGAAAT
GGAATCATATGAAAAAATGCATCAATGTAAATGGTTTCATTTGTGTAATGGTGGATGCCCACACGATCGAGTG
ACCAACAGGAAGCACAATCCAAATTATGATGGTTCATGTTGTGGAACCGGCGGTTTGTTGGAGACAATAAAAC
AAACCATCGCGGCGTAAGCTGCTGCCACCGCTGAGCAATAACTAGCATAACCCCTTGGGGCCTCTAAACGGGT
CTTGAGGGGTTTTTTGCTGAAACCTCAGGCATTTGAGAAGCACACGGTCACACTGCTTCCGGTAGTCAATAAAC
CGGTAAACCAGCAATAGACATAAGCGGCTATTTAACGACCCTGCCCTGAACCGACGACAAGCTGACGACCGG
GTCTCCGCAAGTGGCACTTTTCGGGGAAATGTGCGCGGAACCCCTATTTGTTTATTTTTCTAAATACATTCAAAT
ATGTATCCGCTCATGAATTAATTCTTAGAAAAACTCATCGAGCATCAAATGAAACTGCAATTTATTCATATCAGG
ATTATCAATACCATATTTTTGAAAAAGCCGTTTCTGTAATGAAGGAGAAAACTCACCGAGGCAGTTCCATAGGA
TGGCAAGATCCTGGTATCGGTCTGCGATTCCGACTCGTCCAACATCAATACAACCTATTAATTTCCCCTCGTCAA
AAATAAGGTTATCAAGTGAGAAATCACCATGAGTGACGACTGAATCCGGTGAGAATGGCAAAAGTTTATGCAT
TTCTTTCCAGACTTGTTCAACAGGCCAGCCATTACGCTCGTCATCAAAATCACTCGCATCAACCAAACCGTTATT
CATTCGTGATTGCGCCTGAGCGAGACGAAATACGCGGTCGCTGTTAAAAGGACAATTACAAACAGGAATCGA
ATGCAACCGGCGCAGGAACACTGCCAGCGCATCAACAATATTTTCACCTGAATCAGGATATTCTTCTAATACCT
GGAATGCTGTTTTCCCGGGGATCGCAGTGGTGAGTAACCATGCATCATCAGGAGTACGGATAAAATGCTTGAT
GGTCGGAAGAGGCATAAATTCCGTCAGCCAGTTTAGTCTGACCATCTCATCTGTAACATCATTGGCAACGCTAC
CTTTGCCATGTTTCAGAAACAACTCTGGCGCATCGGGCTTCCCATACAATCGATAGATTGTCGCACCTGATTGC
CCGACATTATCGCGAGCCCATTTATACCCATATAAATCAGCATCCATGTTGGAATTTAATCGCGGCCTAGAGCA
AGACGTTTCCCGTTGAATATGGCTCATACTCTTCCTTTTTCAATATTATTGAAGCATTTATCAGGGTTATTG-3’

References

(1) Wuisan, Z. G.; Kresna, I. D. M.; Böhringer, N.; Lewis, K.; Schäberle, T. F. Optimization of heterologous Darobactin A expression and identification of the minimal biosynthetic gene cluster. *Metabolic engineering* **2021**, *66*, 123–136.

(2) Wang, Y.-S.; Fang, X.; Wallace, A. L.; Wu, B.; Liu, W. R. A rationally designed pyrrolysyl-tRNA synthetase mutant with a broad substrate spectrum. *Journal of the American Chemical Society* **2012**, *134* (6), 2950–2953.

(3) Pham, L. B. T.; Costantino, A.; Barbieri, L.; Calderone, V.; Luchinat, E.; Banci, L. Direct Expression of Fluorinated Proteins in Human Cells for 19F In-Cell NMR Spectroscopy. *Journal of the American Chemical Society* **2023**, *145* (2), 1389–1399.
